# Steroid Transport, Local Synthesis, and Signaling within the Brain: Roles in Neurogenesis, Neuroprotection, and Sexual Behaviors

**DOI:** 10.3389/fnins.2018.00084

**Published:** 2018-02-20

**Authors:** Nicolas Diotel, Thierry D. Charlier, Christian Lefebvre d'Hellencourt, David Couret, Vance L. Trudeau, Joel C. Nicolau, Olivier Meilhac, Olivier Kah, Elisabeth Pellegrini

**Affiliations:** ^1^Université de La Réunion, Institut National de la Santé et de la Recherche Médicale, UMR 1188, Diabète athérothrombose Thérapies Réunion Océan Indien, Saint-Denis de La Réunion, France; ^2^Univ Rennes, Inserm, EHESP, Irset (Institut de recherche en santé, environnement et travail) - UMR_S 1085, Rennes, France; ^3^CHU de La Réunion, Saint-Denis, France; ^4^Department of Biology, University of Ottawa, Ottawa, ON, Canada

**Keywords:** aromatase, cholesterol, blood-brain barrier, estrogens, HDL, lipoproteins, stroke, progestins

## Abstract

Sex steroid hormones are synthesized from cholesterol and exert pleiotropic effects notably in the central nervous system. Pioneering studies from Baulieu and colleagues have suggested that steroids are also locally-synthesized in the brain. Such steroids, called neurosteroids, can rapidly modulate neuronal excitability and functions, brain plasticity, and behavior. Accumulating data obtained on a wide variety of species demonstrate that neurosteroidogenesis is an evolutionary conserved feature across fish, birds, and mammals. In this review, we will first document neurosteroidogenesis and steroid signaling for estrogens, progestagens, and androgens in the brain of teleost fish, birds, and mammals. We will next consider the effects of sex steroids in homeostatic and regenerative neurogenesis, in neuroprotection, and in sexual behaviors. In a last part, we will discuss the transport of steroids and lipoproteins from the periphery within the brain (and vice-versa) and document their effects on the blood-brain barrier (BBB) permeability and on neuroprotection. We will emphasize the potential interaction between lipoproteins and sex steroids, addressing the beneficial effects of steroids and lipoproteins, particularly HDL-cholesterol, against the breakdown of the BBB reported to occur during brain ischemic stroke. We will consequently highlight the potential anti-inflammatory, anti-oxidant, and neuroprotective properties of sex steroid and lipoproteins, these latest improving cholesterol and steroid ester transport within the brain after insults.

## Introduction

Steroid hormones display important physiological functions and exert pleiotropic effects on many target organs including among others the gonads, the liver, and the nervous system. Neurosteroids are produced in the central nervous system (CNS), either via *de novo* synthesis from cholesterol or from local metabolism of steroid intermediate produced in the periphery. The shift from systemic to local synthesis and regulation of steroid action within target tissues, such as the brain, was referred to “Balkanization” of the endocrine system, and could allow the tissue to autonomously synthesize and modulate local steroid signaling (Schmidt et al., 2008). Neurosteroids and peripherally produced steroids have pleiotropic effects and can modulate both brain homeostasis and cerebral functions. The aim of the present review is to summarize the current knowledge regarding the activity and the expression of the steroidogenic enzymes and the targets of steroids, produced in the periphery as well as locally in the brain. We will further consider the roles of androgens, estrogens, and progestagens on physiology and behavior, focusing our discussion on constitutive and regenerative neurogenesis, notably in stroke conditions, as well as the impact of these locally-produced sex steroids on sexual behavior. These points will be discussed from studies performed in fish, birds, and mammals from a comparative point of view. Furthermore, we will discuss the transport of peripheral steroids through the blood-brain barrier (BBB) and their effects on its permeability. We will emphasize the role of this transport by lipoproteins in the functioning of the BBB and during CNS insults, raising the question of the potential roles of cholesterol/steroid transport in neuroprotection and reactive neurogenesis.

## Neurosteroidogenesis in the brain: an overview in fish, birds, and mammals

### Steroidogenesis

**Figure 1:**
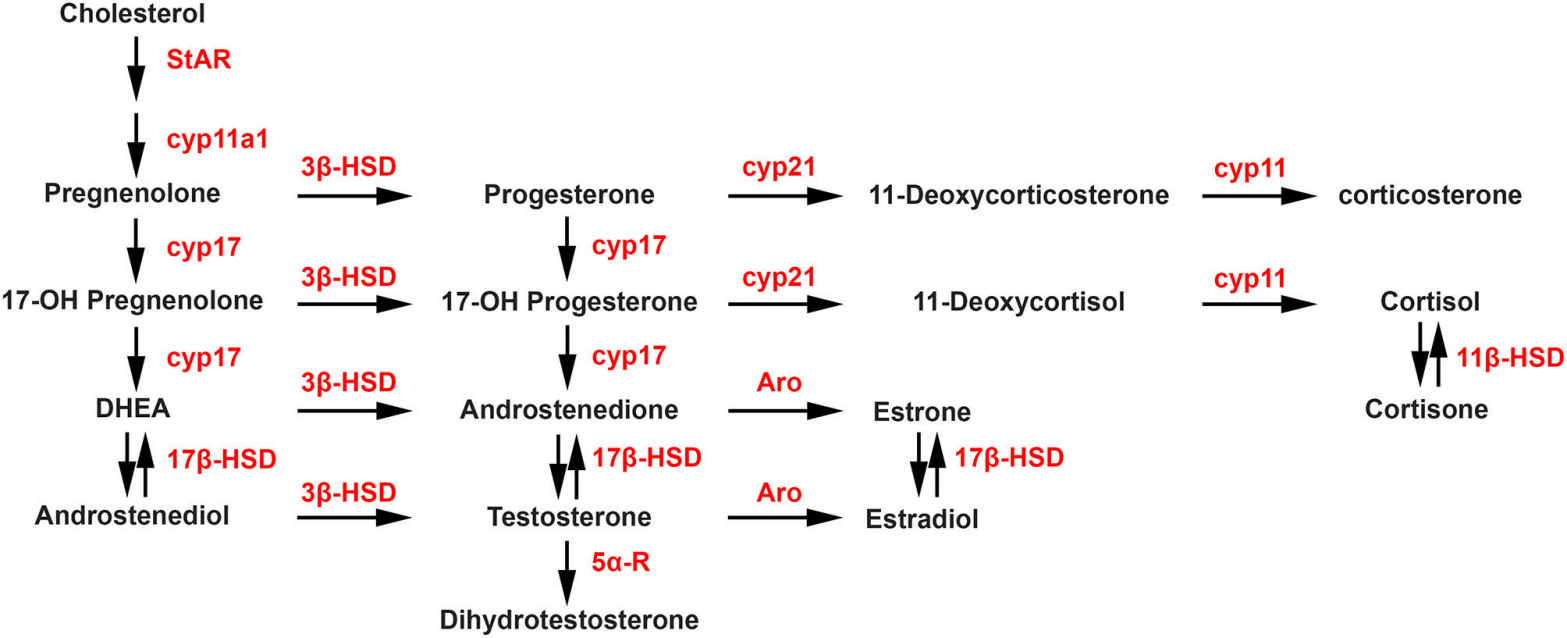
Biosynthetic pathways of neurosteroid synthesis. Cyp11a1, cytochrome P450 family 11 subfamily A member 1 (P450scc), cytochrome P450 side-chain cleavage; Cyp17, cytochrome P450 17α-hydroxylase/C17, 20-lyase; Aro, aromatase; 3β-HSD, 3β-hydroxysteroid dehydrogenase D5–D4 isomerase; 5α-R, 5α-reductase; 17β-HSD, 17β-hydroxysteroid dehydrogenase; Cyp21, cytochrome P450 21 hydroxylase; Cyp11, cytochrome P450 11β-hydroxylase B1 and/or B2. StAR, steroidogenic acute regulatory protein.

Steroidogenesis is the enzymatic process by which cholesterol is converted to biologically active steroid hormones. The steroidogenic acute regulatory protein (StAR), and translocator protein (TSPO), in a complex with various proteins including VDAC and ATAD3A, are involved in the transport of cholesterol to the inner membrane of the mitochondria. The first and rate-limiting enzymatic step of the steroidogenic process is the conversion of cholesterol into pregnenolone by P450 side chain cleavage (P450_scc_; CYP11A1). As shown in the steroidogenic pathway illustrated in Figure 1, pregnenolone can subsequently be converted into progesterone (P) by 3-beta-hydroxysteroid dehydrogenase (3β-HSD) or into 17-hydroxypregnenolone by 17-alpha-hydroxylase/17,20 lyase (CYP17). Then, CYP17 can convert progesterone into 17-hydroxyprogesterone, while 3β-HSD can convert 17-hydroxypregnenolone into 17-hydroxyprogesterone. In a next step, 17-hydroxypregnenolone and 17-hydroxyprogesterone can be converted by CYP17 into dehydroepiandrosterone (DHEA) and androstenedione, respectively. In turn, the activity of different 17β-HSD enzymes catalyzes the synthesis of androstenediol from DHEA and testosterone (T) from androstenedione. Aromatase converts androstenedione and testosterone into estrone (E1) and estradiol (E2), respectively, while 5α-reductase converts testosterone into 5α-dihydrotestosterone (DHT). Considering glucocorticoids, the synthesis of 11-deoxycortisol and 11-deoxycorticosterone from 17-hydroxyprogesterone and progesterone, respectively, are catalyzed by 21-hydroxylase (CYP21A2), followed by the P450C11 (Cyp11, cytochrome P450 11β-hydroxylase B1 and/or B2) activity leading to the synthesis of cortisol and corticosterone. Cortisone can also be converted into cortisol by 11β-HSD.

### Steroidogenic activities in the brains of fish

In the 80s, pioneering studies performed in the goldfish (*Carassius auratus*) and the toadfish (*Opsanus tau*) documented very high brain aromatase and 5α-reductase activities (Pasmanik and Callard, 1985, 1988). High aromatase expression and activity in the brain is a common feature of teleost fish (Kah et al., 2009; Diotel et al., 2010), as reported in the African catfish (*Clarias gariepinus*) (Timmers and Lambert, 1987), the Atlantic Salmon (*Salmo salar*) (Andersson et al., 1988), the three-spined stickleback (*Gasterosteus aculeatus*) (Borg et al., 1989), the European sea bass (*Dicentrarchus labrax*) (Gonzalez and Piferrer, 2003), and the zebrafish (*Danio rerio*) (Goto-Kazeto et al., 2004; Diotel et al., 2011b). The high aromatase activity in the brain of fish is due to the sustained expression of the *cyp19a1b* gene coding for the Cyp19a1b/Aromatase B protein (AroB), while the *cyp19a1a* isoform coding for Cyp19a1a/Aromatase A protein is mainly expressed in gonads. These two isoforms likely resulted from a third round of genomic duplication event occurring ~320–350 million years ago in fish (Ravi and Venkatesh, 2008).

Biochemical studies using RP-HPLC analysis showed that the brains of adult zebrafish is able to convert [^3^H]-pregnenolone into [^3^H]-progesterone, documenting subsequently 3β-HSD activity in the brain of this teleost. These data were reinforced by the absence of progesterone synthesis following treatment with trilostane, a specific inhibitor of 3β-HSD (Sakamoto et al., 2001a). Ten years later, using similar methods, it was shown that the adult zebrafish brain can convert [^3^H]-pregnenolone into a wide variety of radiolabeled steroids including 17OH-pregnenolone, progesterone (P), and tetrahydro-P, DHEA, androstenedione, testosterone (T), dihydrotestosterone (DHT), 17β-estradiol (17β-E2), and also estrone (E1) (Diotel et al., 2011b). Expression and activity studies in the brain of adult zebrafish were documented for 3α-Hsd, 3β-Hsd, 17β-Hsd, Cyp17, AroB, and 5α-reductase (Diotel et al., 2011b). The recent reanalysis of the RP-HPLC profiles previously reported by Diotel et al. (2011a) indicated that [^3^H]-pregnenolone can also be converted into cortisol and tetrahydrodeoxycorticosterone (THDOC), further highlighting Cyp21A2 and Cyp11C1 activities within the brain of adult zebrafish (Weger et al., accepted).

Taken together, these data demonstrate that the brain of adult fish is able to *de novo* synthesize a wide variety of steroids from pregnenolone, suggesting that the substrates available for steroidogenesis can originate from local synthesis within the brain, and also from the conversion of peripherally produced precursors.

### Steroidogenic enzyme expression in the brains of fish

The first attempts to localize aromatase-expressing cells in the brain of fish were performed in goldfish using antibodies raised against human placental aromatase (Gelinas and Callard, 1997). In this work, neuron-like cells were labeled in the olfactory bulb, the telencephalon, the preoptic area, and the hypothalamus. However, *cyp19a1b in situ* hybridization and double immunohistochemistry using specific AroB antibodies and glial (GFAP, S100β, and BLBP) or neuronal markers (HuC/D or NeuN) clearly demonstrated the exclusive glial nature of AroB^+^ cells in others teleost species (Forlano et al., 2001; Goto-Kazeto et al., 2004; Forlano and Bass, 2005; Strobl-Mazzulla et al., 2005; Pellegrini et al., 2007; Mouriec et al., 2008; Tong et al., 2009; März et al., 2010; Coumailleau et al., 2015; Diotel et al., 2016). These data were further reinforced by the development of a *cyp19a1b*-GFP transgenic zebrafish line, showing the exclusive GFP co-expression with glial markers (Tong et al., 2009). Interestingly, AroB^+^ cells correspond to radial glial cells (RGCs), a particular type of cells with a characteristic morphology. Radial glial cells display a soma localized in the vicinity of the ventricular layer and exhibit two cytoplasmic processes, a short one extending to the ventricle and a longer one running through the brain parenchyma toward the pial surface. In mammals, RGCs behave as neural stem cells (NSCs) during embryonic development and transform into astrocytes at the perinatal stage (Noctor et al., 2001; Weissman et al., 2003; Kriegstein and Alvarez-Buylla, 2009). In fish, they persist during adulthood and maintain neural progenitor properties (Adolf et al., 2006; Pellegrini et al., 2007, 2013, 2015; Tong et al., 2009; März et al., 2010; Rothenaigner et al., 2011; Kizil et al., 2012; Diotel et al., 2016).

Other studies performed in zebrafish documented the expression of 3β-Hsd in neuronal soma and fibers throughout the brain (i.e., dorsal telencephalon, thalamus, preoptic area, paraventricular organ), the cerebellum and the spinal cord (Sakamoto et al., 2001a). In the African lungfish (*Protopterus annectens*), 3β-Hsd was also shown to be expressed in neurons while 5α-reductase was detected in both glia and neurons (Mathieu et al., 2001). Furthermore, numerous experiments documented the expression of the steroidogenic enzymes (*P450scc, 3*β*-hsd, cyp17*, and *cyp19a1b*) in the brains of fish including the Tongue sole (*Cynoglossus semilaevis*) and the Black porgy (*Acanthopagrus schlegeli*) (Tomy et al., 2007; Chen et al., 2010b). In adult zebrafish, *in situ* hybridization experiments indicate that there is a wide and overlapping distribution of *P450scc, 3*β*-hsd, cyp17*, and *cyp19a1b* throughout the brain particularly in the telencephalon, the preoptic area, the hypothalamus, and the cerebellum (Diotel et al., 2011b). The distributions of *P450scc, 3*β*-hsd*, and *cyp17* suggest a potential expression in neurons as well as in neural progenitors, as shown by co-expression with AroB, a RGC marker. From 2011 until now, new isoforms for these steroidogenic enzymes have been identified: *3-βhsd1, 3-βhsd2, cyp11a1, cyp11a2, cyp17a1*, and *cyp17a2*. Recent work confirmed the sites of expression of these steroidogenic enzymes in the brain of adult zebrafish and further described the distribution of their respective isoforms (Weger et al., accepted). It appears that all these steroidogenic enzymes display overlapping distributions. In addition, expression of *cyp21a2, cyp11c1*, and *fdx1/fdx1b* (co-factors of glucocorticoid synthesis) was recently described in adult zebrafish brain (Weger et al., accepted), and patterns appeared similar to other steroidogenic enzymes. Furthermore, isolation and culture of goldfish RGCs was performed (Xing et al., 2014), and deep RNA sequencing of these RGCs indicates expression of steroidogenic acute regulatory protein (*star*), *cyp11a1, cyp17a1, fdx1, hsd17b10* in addition to *cyp19a1b* and *5*α*-reductase* (Da Fonte et al., 2017). Proteomic analyses also revealed the production of 20β-Hsd (Xing et al., 2016).

**Figure 2:**
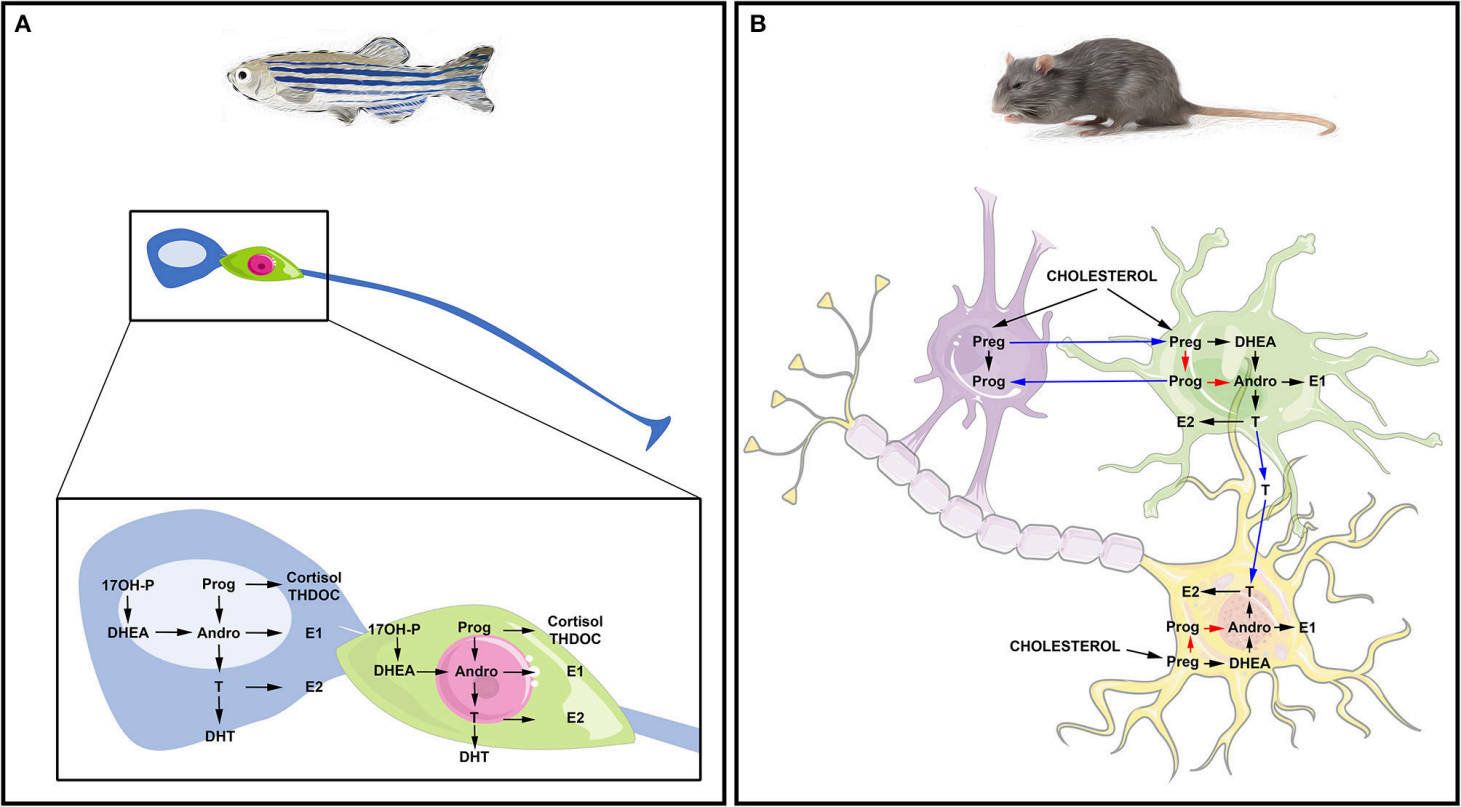
Steroidogenesis in the brain of fish and mammals. **(A)** Steroidogenesis in the brain of adult fish. The blue cell corresponds to a radial glia cell acting as neural stem cell, while the green cell corresponds to a neuron. Data obtained by *in situ* hybridization, RNA sequencing and/or proteomics show neuronal and radial glial expression of steroidogenic enzymes involved in the synthesis of 17OH-Pregnenolone (17OH-P), Progesterone (P), dehydroepiandrosterone (DHEA), Androstenedione (Andro), Estrone (E1), 17β-estradiol (17β-E2, called here E2), testosterone (T) and dihydro-testosterone (DHT), cortisol and THDOC (tetrahydrodeoxycorticosterone). **(B)** Steroidogenesis in the brain of rat. The purple cell corresponds to an oligodendrocyte that participates in the myelin sheath genesis of axons; the green cell is an astrocyte and the yellow one is a neuron whose axon is myelinated by the oligodendrocyte. This schematic view adapted from Zwain and Yen (1999) highlights potential interaction of oligodendrocytes, astrocytes, and neurons in neurosteroidogenesis. The main steroidogenic pathway is shown by black arrows, the minor ones by red arrows and the suggested ones by blue arrows referring to the work of Zwain and Yen (1999). Note that arrows do not necessarily document the direct conversion of the one steroid into another, but can include several enzymatic processes.

Altogether, these studies suggest that the brains of adult fish widely express the biologically active steroidogenic enzymes leading to sex steroid synthesis. They also suggest that RGCs in fish may be capable of *de novo* steroid production from cholesterol and could be consequently envisioned as true steroidogenic cells (Figure 2A).

### Steroidogenic enzyme activities and expression in the brains of birds

Birds are excellent models for understanding the impact of peripherally and locally produced steroids on brain functions, including behavior. Pioneering studies from the groups of Tsutsui and Schlinger have shown that the brains of the quail (*Coturnix japonica*) and zebra finch (*Taeniopygia guttata*) are capable of *de novo* steroidogenesis. They showed that the avian brain expresses the active P450scc, 3β-HSD, CYP17, 17β-HSD, and aromatase enzymes, leading to the production of a wide variety of neurosteroids including pregnenolone, progesterone, androstenedione, testosterone, and estradiol from cholesterol (Tsutsui and Yamazaki, 1995; Tsutsui et al., 1999, 2006, 2009; Matsunaga et al., 2001; London et al., 2006, 2009; London and Schlinger, 2007; Tsutsui, 2011; Schlinger and Remage-Healey, 2012). Western-Blot and immunohistochemistry experiments allow the detection of P450scc immunoreactive neurons through the preoptic area, the thalamus, the hypothalamus, and the cerebellum in Purkinje cells (Tsutsui, 2011). 3β-HSD expression was highlighted based on enzymatic activity in the brain of the adult quail, with a strong presence in the telencephalon and the diencephalon and a lower one in the mesencephalon (Ukena et al., 1999). In quail, *Cyp17* gene expression is detected in the preoptic area, the thalamus, the hypothalamus, and the optic tectum as well as in Purkinje cells (Matsunaga et al., 2001).

Aromatase expression was thoroughly studied in several brain regions particularly, in the hypothalamus and the preoptic area in quail (Balthazart and Foidart, 1993; Balthazart et al., 1996; Balthazart and Ball, 1998; Tsutsui, 2011) and in the telencephalon in zebra finches (Schlinger, 1997). Interestingly, data showed overlapping distribution of *StAR, CYP11A1, 3*β*-HSD*, and *CYP17 mRNA* in the ventricular proliferative zone of the avian brain during support (London and Schlinger, 2007), suggesting potential roles of neurosteroids in neurogenesis and CNS development. It should be noted that 3β-HSD is also present throughout the telencephalon in adult song birds (Soma et al., 2004; Schlinger et al., 2008). Furthermore, aromatase expression was also studied throughout the avian brain during support and *de novo* expression was documented in RGCs following brain injury in adult birds (Peterson et al., 2004). These data surface a potential role of aromatase and estrogen synthesis in neuroprotection and brain repair mechanisms.

These different studies highlight the capacity of the avian brain to synthesize its own steroids and suggest key roles of neurosteroids in reproductive behavior and neurogenesis.

### Steroidogenic enzyme activities and expression in the brains of mammals

*P450*_*scc*_ gene expression was detected in numerous brain regions of the rodent (i.e., cerebral cortex) and human brain (i.e., cerebral cortex, olfactory bulbs, hippocampus, cerebellum) (Pelletier, 2010). Cells immunoreactive for P450_scc_ were observed in the white matter, the pyramidal and granular neurons of the developing and adult hippocampus (a brain region known for maintaining active neurogenesis during adulthood), and also in Purkinje cells from the cerebellum (Le Goascogne et al., 1987; Tsutsui and Ukena, 1999; Kimoto et al., 2001).

In the 80s, 3β-HSD activity was reported in rat brain homogenates through the conversion of pregnenolone into progesterone (Weidenfeld et al., 1980). More generally, 3β-HSD gene and protein expressions were documented in primary cultures of rodent oligodendrocytes and neurons, and distributed especially in the olfactory bulbs, the thalamus, the cerebral cortex, the hypothalamus, and the cerebellum (Dupont et al., 1994; Guennoun et al., 1995; Meffre et al., 2007; Pelletier, 2010). In humans, *3*β*-HSD* gene expression was detected in neuronal, astroglial, and oligodendrocyte cell lines (Brown et al., 2000) and had a similar distribution compared to rodents, notably in the thalamus, the hippocampus, and the cerebellum (Yu et al., 2002).

The presence of Cyp17 in the mammalian central nervous system (CNS) is currently not clear. Indeed, some work suggested the absence of *Cyp17* gene expression while other studies reported *Cyp17* gene expression in the cerebellum and the brainstem of rat, and showed Cyp17 immuno-positive cells in the hippocampus, the hypothalamus, and in the Purkinje cells (Mellon and Deschepper, 1993; Strömstedt and Waterman, 1995; Hojo et al., 2004; Pelletier, 2010). During development, the expression of *Cyp17* in the rat brain is present but decreases postnatally (Compagnone and Mellon, 2000). Hence, the question of Cyp17 expression and activity in the mammalian brain remains open.

In rodents, aromatase expression (mRNA and protein) and activity were documented in numerous brain regions including the cerebral cortex, the preoptic nucleus, the hypothalamus as well as the hippocampus; in humans, it was also detected in the pons, the thalamus, the hypothalamus, and the hippocampus (Lephart et al., 1992; Lephart, 1996; Sasano et al., 1998; Garcia-Segura et al., 2003; Hojo et al., 2004; Yague et al., 2006; Pelletier, 2010). In rodents, aromatase expression was mainly detected in neurons and not in glial cells, except following brain injury that results in a *de novo* aromatase expression by astrocytes, suggesting a role of estrogens in neuroprotection (Garcia-Segura et al., 1999; Azcoitia et al., 2001; Garcia-Segura, 2008). In contrast to rodents, in some primates including humans, the estrogen-synthesizing enzyme expression was also reported in some subpopulations of astrocytes in addition to neurons (Yague et al., 2006; Roselli, 2007). As reviewed by Pelletier (2010), numerous studies demonstrate aromatase activity in the brain of mammals including primates.

Consequently, *de novo* steroid synthesis occurs in the brains of mammals (Figure 2B). Neurosteroidogenesis is active in numerous regions including the hippocampus, known for maintaining an intense neurogenic activity during adulthood. Interestingly, this region was shown to synthesize 17β-E2 from pregnenolone (Hojo et al., 2004), reinforcing the idea that steroids could impact neurogenesis.

In conclusion, neurosteroidogenesis appears to be a conserved process well-documented in fish, birds and mammals (Figure 2). It occurs in numerous evolutionary conserved regions such as the telencephalon, the diencephalon (i.e., preoptic area and hypothalamus), and also the cerebellum. This sustained synthesis of steroids may regulate several brain functions and participate to brain homeostasis, neurotransmission, neurogenesis, and brain plasticity by targeting different cell types.

## Steroid signaling in the brain: focus on the brains of fish, birds, and mammals

Peripherally and locally produced steroids can target different brain regions and cell-types expressing their respective and specific receptors. Here, we will describe the main sites of expression of steroid receptors, with a special focus on estrogen, progesterone, and androgen receptors. We will first discuss receptor distributions in the brain of fish and birds before highlighting the situation in mammals.

### Expression of nuclear and membrane-associated steroid receptors in the brains of fish

#### Nuclear estrogen receptors (ER)

Unique among vertebrates, fish have three nuclear estrogen receptors resulting from an ancient genome duplication: one ERα and two ERβ. The ERα and ERβ probably arose from a first duplication event prior the emergence of ray-finned species. A second duplication led to the emergence of two *ERβ* and *ERα* genes but the second copy of the *ERα* probably disappeared (Thornton, 2001; Bardet et al., 2002; Nagler et al., 2007).

Nuclear estrogen receptors genes [*ERα* (*esr1*), *ERβ1* (*esr2b*), and *ERβ2* (*esr2a*)] were identified in many teleost species, including the rainbow trout (*Oncorhynchus mykiss*), tilapia (*Oreochromis niloticus*), medaka (*Oryzias latipes*), goldfish, and zebrafish among others (Pakdel et al., 1990; Tan et al., 1995; Muñoz-Cueto et al., 1999; Tchoudakova et al., 1999; Xia et al., 1999; Hawkins et al., 2000; Kawahara et al., 2000; Ma et al., 2000; Socorro et al., 2000; Bardet et al., 2002; Lassiter et al., 2002; Menuet et al., 2002; Andreassen et al., 2003; Choi and Habibi, 2003; Halm et al., 2004; Sabo-Attwood et al., 2004; Filby and Tyler, 2005; Forlano et al., 2005; Nagler et al., 2007; Strobl-Mazzulla et al., 2008; Chakraborty et al., 2011; Zhang et al., 2012; Katsu et al., 2013).

Most studies carried out so far reported the presence of *esr* transcripts in the brain of adult fish. *In situ* hybridization and immunohistochemistry experiments used to localize ER-expressing cells in the brain revealed distinct but partly overlapping patterns of expression in many fish species including medaka, Atlantic croaker (*Micropogonias undulatus*), pejerrey (*Odontesthes argentinensis*), trout and zebrafish (Salbert et al., 1991; Anglade et al., 1994; Hawkins et al., 2000, 2005; Menuet et al., 2002; Pellegrini et al., 2005; Nagler et al., 2007; Strobl-Mazzulla et al., 2008; Zempo et al., 2013; Coumailleau et al., 2015). In general, transcripts for ERs have a broad distribution and were detected in many brain regions known to regulate sexual, reproductive, social behaviors, and sensory-motor activities such as the telencephalon, the preoptic area, the hypothalamus, and the cerebellum. Additionally, ER transcripts have been reported in cells lining the ventricles, where RGCs are localized, and also in cells localized more deeply in the brain parenchyma. Interestingly, there are clear evidences that ERα is expressed in dopaminergic, GABAergic, kisspeptin- and neuropeptide B-positive neurons in several species (Linard et al., 1996; Anglade et al., 1999; Mitani et al., 2010; Escobar et al., 2013; Zempo et al., 2013; Hiraki et al., 2014). Similarly to rodents, ERs were not reported in neurons expressing gonadotrophin-releasing hormone (Navas et al., 1995).

In midshipman (*Porichthys notatus*), pejerrey, and zebrafish, the pattern of expression of the nuclear estrogen receptors and AroB (Cyp19a1b) are closely related (Forlano et al., 2005; Strobl-Mazzulla et al., 2008; Diotel et al., 2011a). Careful examination of adjacent sections hybridized with *cyp19a1b* and *esr1* riboprobes in trout and zebrafish failed to evidence any co-localization (Menuet et al., 2003, 2005). However, *esr2b* was recently shown to be expressed in AroB-expressing RGCs (Pellegrini et al., 2015), and RT-PCR analysis performed in glial cells enriched cultures from adult trout or goldfish suggested that a weak expression of ERα could not be excluded (Menuet et al., 2003; Xing et al., 2015). A recent study designed to characterize the transcriptome of cultured goldfish RGCs and reported *esr1, esr2b*, and *esr2a* expression in these neural progenitors (Da Fonte et al., 2017).

Knowledge about estrogen signaling during early development in fish is very limited and the zebrafish is the only species in which expression of the three ERs during embryogenesis was monitored. Quantitative-PCR experiments established that *esr1, esr2a*, and especially *esr2b* mRNA were maternally inherited and expressed in eggs before dropping down to 24 h post-fertilization (hpf) (Bardet et al., 2002; Lassiter et al., 2002; Mouriec et al., 2009; Bondesson et al., 2015). Nuclear estrogen receptor expression then started to increase at 24 h, when the onset of zygotic transcription is activated (Bardet et al., 2002; Lassiter et al., 2002; Mouriec et al., 2009). Whole mount *in situ* hybridization showed that *esr2a* and *esr2b* mRNA were detectable at 48 hpf in the ventral telencephalon and in the presumptive preoptic area as well as in the hypothalamus, in line with what was described in the adult brain (Mouriec et al., 2009). A clear expression for *esr1* is described in these regions but at later stages, between 14 days post-fertilization (dpf) and 21 dpf (Kallivretaki et al., 2007; Mouriec et al., 2009). Using *cyp19a1b*-GFP zebrafish embryos, GFP expression was shown to be inducible in RGCs at 24 h after 17β-E2 treatment, an effect blocked by the ER antagonist ICI 182,780, indicating that ERs were fully functional in the brain early during development (Mouriec et al., 2009). A morpholino approach confirmed the specific role of *esr2b* in the induction of the *cyp19a1b* gene (Griffin et al., 2013). Temporary disruption of *esr2a* expression in zebrafish with the same technological approach was associated with a developmental defect of sensory hair cells (Froehlicher et al., 2009). Knockdown of both maternally inherited and zygotic *esr2a* was associated with an increase in apoptotic cells particularly in the brain leading to severe brain defects (Celeghin et al., 2011).

#### Membrane-associated estrogen receptors (mER)

Membrane receptors responding to 17β-E2 stimulation via rapid non-genomic signaling have been characterized in fish. G protein-coupled estrogen receptor 1 (GPER or GPR30) is a member of this receptor family. A partial or full-length *gper* was cloned, characterized, and detected in the brain of adult zebrafish, Atlantic croaker, and goldfish (Pang et al., 2008; Liu et al., 2009; Pang and Thomas, 2009; Mangiamele et al., 2017). In zebrafish, *gper in situ* hybridization on adult brain sections showed a specific pattern of expression in the olfactory bulbs, the telencephalon, the hypothalamus, the optic tectum, the cerebellum, and the medulla oblongata (Liu et al., 2009). In goldfish, *gper* was also expressed in the forebrain and the suprachiasmatic nucleus, the preoptic area, and the optic tectum (Mangiamele et al., 2017). Originally, it was proposed that goldfish RGCs did not express the G-protein coupled estrogen receptor because attempts to amplify a specific cDNA from cultured cells failed (Xing et al., 2015). However, whole RNA sequencing of separate primary cultures of these cells revealed expression of *gper1*, suggesting that some subpopulations of RGCs express the membrane ER (Da Fonte et al., 2017).

The spatiotemporal distribution of *gper* was investigated in zebrafish using PCR. Its expression was observed in the anterior diencephalon, the midbrain and the hindbrain (Jayasinghe and Volz, 2012; Shi et al., 2013). Both *gper* and *esr2b* mRNA are expressed in neuromasts, suggesting a role of estrogens and a close interaction between these receptors in the developmental regulation of this mecanoreceptive organ (Froehlicher et al., 2009). Knock-down of *gper* resulted in morphological defects in the zebrafish brain at 24 hpf (Shi et al., 2013). At 30 hpf, these morphants displayed an increased number of apoptotic cells, and a decreased brain cell proliferation (Shi et al., 2013). A slight reduction in *otx2* gene expression, which is required for sensory organs development and brain function, was also observed in *gper* knock-down zebrafish. Taken together, these data show the potential involvement of both nuclear and membrane-associated estrogen receptors in brain development and functions.

#### Nuclear progestin receptors (PR)

Nuclear progestin receptor (PR) genes have been characterized in a few fish species (Ikeuchi et al., 2002; Morini et al., 2017). In European eel (*Anguilla Anguilla*), two PR genes, *pgr1* and *pgr2*, were differentially expressed in the brain (olfactory bulb, telencephalon, diencephalon, cerebellum) and in the pituitary of immatures males and females (Morini et al., 2017). Two genes were also identified in the Japanese eel (*Anguilla japonica*) but only *pgr2* mRNA was detected in the brain (Ikeuchi et al., 2002). In zebrafish, a single locus encoding PR (Pgr) was identified (Chen et al., 2010a; Hanna et al., 2010). Ontogenic expression analysis determined by PCR during early embryogenesis showed that *pgr* mRNA was not maternally inherited and became detectable in embryos at 8 hpf (Chen et al., 2010a). Immunohistochemistry and *in situ* hybridization revealed a widespread distribution of zebrafish Pgr receptor (protein and mRNA) in the brain especially neuroendocrine regions (olfactory bulbs, preoptic area, telencephalon, thalamus, hypothalamus, optic tectum, torus longitudinalis, valvula cerebelli) (Hanna et al., 2010; Diotel et al., 2011c). It should be noted that Pgr-positive cells were observed along the ventricular cavities and also more deeply in the brain parenchyma. In this work, it was shown that periventricular Pgr-positive cells displayed a stronger staining compared to Pgr-positive cells located deeply in the parenchyma (Hanna et al., 2010; Diotel et al., 2011c). By performing double immunostainings, it was shown that a large portion of Pgr-positive cells located in the cerebral parenchyma correspond to acetylated-tubulin positive neurons (Diotel et al., 2011c). In contrast, immunohistochemistry on *cyp19a1b*-GFP transgenic zebrafish clearly showed Pgr-expressing cells along the ventricle correspond to AroB^+^ RGCs (Diotel et al., 2011c). These data were confirmed by a transcriptomic analysis showing *pgr* mRNA expression in cultured goldfish RGCs (Da Fonte et al., 2017). Pgr expression is consequently high in the estrogen-synthesizing RGCs. This suggests a relationship between locally-produced estrogens and Pgr expression. Indeed, treatments with the aromatase inhibitor ATD or with 17β-E2 respectively decrease or increase *prg* expression in the brains of adult zebrafish and also in larvae (Diotel et al., 2011c).

Gene knockout using the TALENs strategy allowed to generate zebrafish lines with null Pgr expression to assess the *in vivo* function of this receptor. Unfortunately, all studies performed with these models have focused at the gonadal levels and none of them looked at the effect of Pgr expression disruption in the brain (Zhu et al., 2015; Tang et al., 2016; Wang et al., 2016).

#### Membrane-associated progestin receptors (mPR)

In mammals, many progestins exert non-genomic effects through rapid activation of intracellular signaling pathways mediated by two groups of membrane progestin receptors: mPRs (belonging to the PARQ family) and PGRMC1 and PGRMC2 (Petersen et al., 2013). However, there are only scarce data on membrane-associated progestin receptors existence and sites of expression in the brain and the pituitary of fish (Thomas, 2008).

The *mPR*α was identified and detected in the brain of adult spotted seatrout (*Cynoscion nebulosus*) (Zhu et al., 2003). It was also recently cloned in medaka but no data about its brain expression are available (Roy et al., 2017). In goldfish, *mPR*α, *mPR*β, *mPR*γ*1*, and *mPR*γ*2* were also detected in the brain (Tokumoto et al., 2006, 2012). Other studies documented the expression of different mPRs and/or PGMRCs in the brain and the pituitary of the European eel (*Anguilla anguilla*), the channel catfish (*Ictalurus punctatus*) and also the rainbow trout (Kazeto et al., 2005; Mourot et al., 2006; Morini et al., 2017). To date, mPRα and mPRβ were the only form to be identified in zebrafish (Zhu et al., 2003). Using specific antibodies for zebrafish mPRα and mPRβ, Hanna and Zhu demonstrated their expression in brain samples (Hanna and Zhu, 2009). Currently, the distributions and functions of mPR in the brain of fish, if any, are completely unknown.

#### Nuclear androgen receptors (AR)

Two *ar* genes (*ar*1 and *ar*2) were isolated and characterized in several fish species including the Japanese eel (*Anguilla japonica*), the rainbow trout, the kelp bass (*Paralabrax clathratus*), the Atlantic croaker, and cichlids (Ikeuchi et al., 1999; Sperry and Thomas, 1999a,b; Takeo and Yamashita, 1999, 2000; Harbott et al., 2007). More recently, only one *ar* isoform was described in the stickleback (Olsson et al., 2005), the wrasse (*Halichoeres trimaculatus*) (Kim et al., 2002), the zebrafish (Jorgensen et al., 2007; de Waal et al., 2008; Gorelick et al., 2008; Hossain et al., 2008), and in the plainfish midshipman (Forlano et al., 2010).

The first immunohistochemistry studies carried out on the brains of adult goldfish with an heterologous antibody revealed AR-positive cells with neuronal appearance in neuroendocrine regions such as the preoptic area and the hypothalamus, as well as in the olfactory bulbs, the telencephalon and the optic tectum (Gelinas and Callard, 1997). More detailed results were obtained in the brain of the plainfin midshipman, a fish that express mating behavior triggered by auditory signal. These data showed mRNA expression in auditory-related nuclei of the telencephalon, hypothalamus, midbrain, and in the vocal prepacemaker and vocal motor nuclei (Forlano et al., 2010). Numerous studies showed AR expression in the brain of fish including among others wrasse and zebrafish (Kim et al., 2002; Harbott et al., 2007; Hossain et al., 2008; Pouso et al., 2010).

In adult zebrafish, *ar* is strongly expressed along the ventricle and within the brain parenchyma of the telencephalon, the preoptic area, and the hypothalamus (Gorelick et al., 2008). In embryos, *ar* trancripts are maternally deposited and expression levels start to increase substantially at 24 hpf (Hossain et al., 2008). The *ar* gene expression is detected at 24 hpf in the olfactory placodes and in the midbrain, at 48 hpf in the medial diencephalon and at 3 dpf in the pineal organ (Gorelick et al., 2008). All neuroanatomical studies undertaken to date show that ARs are expressed in ventricular margins, where RGC are located (Harbott et al., 2007; Gorelick et al., 2008; Forlano et al., 2010; Pouso et al., 2010). Recently, transcriptomic analysis showed the expression of *ar* in goldfish RGCs (Da Fonte et al., 2017).

As mentioned above, it was shown that goldfish RGCs express progesterone receptor (Pgr), androgen receptor (*ar*), estrogen receptor α (*esr1*), estrogen receptor β*1* (*esr2b*), and estrogen receptor β*2* (*esr2a*) (Da Fonte et al., 2017). This data supports other reports in zebrafish showing that RGCs express Pgr protein (Diotel et al., 2011c) and observations for the expression of *ar, esr1, esr2a*, and *esr2b* mRNAs within neurons and/or AroB^+^ RGCs (Diotel et al., 2011a; Pellegrini et al., 2015). All this suggests that RGCs are both a source and a target of neurosteroids (Diotel et al., 2011a; Pellegrini et al., 2015; Xing et al., 2016), arguing for key roles of steroids in RGC activity.

### Expression of nuclear and membrane-associated steroid receptors in the brains of birds

#### Nuclear and membrane-associated estrogen receptors

Early data on the distribution of sex hormone receptors in the brain of birds was extensively reviewed (Gahr, 2001). Similar to mammals, there are two different avian genes coding for ERα and ERβ (Bernard et al., 1999; Jacobs et al., 1999). In birds, ERα is expressed in the brain of all the species studies so far, including members of Apodiformes, Passeriformes, Galliformes, Columbiformes, and Psittaciformes. These ERs are distributed in various brain territories including the telencephalon, the diencephalon, and the rhombencephalon, with more noticeable expression in the preoptic area and the hippocampus (Gahr, 2001). For instance, in the brain of zebra finch, ERα mRNAs was observed in the nidopallium, the arcopallium, the hippocampus, the diencephalon, the midbrain and within the vocal control circuitry (Jacobs et al., 1999). Interestingly, ERα mRNAs expression overlap with that of aromatase (Jacobs et al., 1999). ERβ was first described in the brain of the Japanese quail, the European starling (*Sturnus vulgaris*), and the canary (Bernard et al., 1999; Foidart et al., 1999; Gahr, 2001). In general, ERβ was reported in the preoptic area, the hypothalamus, the thalamus and different midbrain nuclei. In the European starling, *in situ* hybridization experiments allowed the detection of ERβ mRNA in the nidopallium and the preoptic area in a pattern reminiscent of aromatase (Bernard et al., 1999; Axelsson et al., 2007). Consequently, ERα, ERβ and aromatase distributions strongly overlap. As reported in oscine birds, ER labeling was detected in different forebrain regions while it was not found in non-oscine ones, highlighting discrete differences in ER expression in songbird species and families (Gahr, 2001). It should be noted that, while histological studies have focused on the distribution of the nuclear isoforms, numerous functional studies suggest that estrogen receptors act at the level of the membrane, probably via interaction with other receptors such as glutamate receptors (Pawlisch and Remage-Healey, 2015; Seredynski et al., 2015).

#### Nuclear and membrane-associated progesterone receptors

Concerning PR expression, most studies in birds have focused on the hypothalamic region, because of the potential role of PR signaling in reproductive and egg-laying behaviors. Similarly to ER and AR, PR expression was investigated and documented by *in situ* hybridization and immunohistochemistry in the preoptic area, the hypothalamus, the thalamus, the hippocampus of the zebra finch, and the hen (Sterling et al., 1987; Lubischer and Arnold, 1990).

#### Androgen receptors

The first studies documenting AR distribution in the brain of birds were performed by *in vivo* autoradiography with radio-labeled steroids (Arnold, 1980; Gahr, 2001). In zebra finch, these autoradiographic studies demonstrated the binding of tritiated testosterone in the HVC (used as the proper term, but previously known as hyperstriatum ventrale pars caudalis or and high vocal center), the magnocellular nucleus of the anterior nidopallium, the robust nucleus of the arcoplallium, the nucleus intercollicularis of the midbrain, and the periventricular magnocellular nucleus of the anterior hypothalamus (Arnold, 1980). In the golden-collared manakins (*Manacus vitellinus*), *in situ* hybridization for AR revealed expression in the forebrain, in the nucleus taeniae of the amygdala and in the arcopallium (Fusani et al., 2014). In general, ARs are detected in the hypothalamus, the preoptic area, the midbrain, and hindbrain in all species studied to date.

Taken together, it appears that ER, AR, and PR display almost similar distribution in the avian brain, with higher expression in the preoptic area, hypothalamus, thalamus, and hippocampus.

### Expression of nuclear and membrane-associated steroid receptor in the brains of mammals

#### Estrogen receptors

Data concerning ERs mRNA and protein expression mostly originate from rat studies showing a strong expression of ERα and ERβ throughout the brain (Li et al., 1997; Shughrue et al., 1997). *In situ* hybridization and immunohistochemistry experiments revealed that ERα and ERβ are expressed in neurons and glia from many CNS regions including the olfactory bulbs, the preoptic area, the hypothalamus, the zona incerta, the ventral tegmentum as well as the cerebellum in Purkinje cells (Li et al., 1997; Shughrue et al., 1997; Mitra et al., 2003; Frick et al., 2015). Both ERs were also detected in the cerebral cortex and in the hippocampus, with higher ERβ expression compared to ERα (Shughrue et al., 1997). In the brain of mouse, immunohistochemistry against ERβ allows the detection of immunoreactive cells in regions similar to the rat. However, ERα expression appears stronger in the hippocampus and the hypothalamus, and lower in the cerebral cortex and cerebellum (Mitra et al., 2003), suggesting some differences in ER signaling between rat and mouse. Importantly, ERs are widely and strongly expressed in the brain of mouse at postnatal day 7, but their respective expression declines the two following days with different kinetics (Sugiyama et al., 2009). In humans, ERα is detected in the cortex at 9 gestational weeks in proliferating zones and in the cortical plate. Its expression subsequently decreases until birth before increasing gain during adulthood. At 15 gestational weeks, ERβ was also detected in proliferating zones and in the cortical plate with expression persisting in the adult cortex (González et al., 2007). Interestingly, from 15 gestational weeks to adulthood, ERα and ERβ are detected in the hippocampus including the dentate gyrus, known to be a neurogenic region (González et al., 2007). In the same line, ERs have been detected in embryonic/adult NSCs in rodents and humans, suggesting a roles of estrogen signaling in NSC activity (Brännvall et al., 2002; Kishi et al., 2005; Hajszan et al., 2007; Suzuki et al., 2007; Okada et al., 2010). The spatiotemporally regulated expression of ERs in the embryonic brain of mouse and human, notably in the cerebral cortex and the hippocampus, argue in favor of different roles of estradiol during development (González et al., 2007; Sugiyama et al., 2009). Furthermore, a plasma membrane-associated ER (ER-X, GPER, GPR30, GPER1) was identified in the brain, mediating estrogen activation of MAPK/ERK (Toran-Allerand, 2005). This receptor is functionally distinct from ERα and ERβ and is up-regulated in the adult brain after ischemic stroke (Toran-Allerand et al., 2002). Its expression in the hippocampus is likely to play a role in synaptic plasticity (Waters et al., 2015).

#### Progesterone receptors

In mammals, progesterone receptors include the classic nuclear PRA and PRB receptors, splice variants, and membrane-associated receptors mPR (mPRα, mPRβ, and mPRγ) (Brinton et al., 2008). Nuclear PRs are expressed through the brain in neurons and glial cells of the hippocampus, the cortex and the hypothalamus (Hagihara et al., 1992a,b; Brinton et al., 2008), and their expression is up-regulated by estrogens in a region specific manner (Guerra-Araiza et al., 2003). Among the three mPR subtypes described, mPRα is the best characterized pharmacologically. *In situ* hybridization and immunohistochemistry experiments have shown a wide mPRα neuronal expression in the olfactory bulbs, the cortex, the hypothalamus, the hippocampus, and the cerebellum (Meffre et al., 2013). Very interestingly, after traumatic brain injury, mPRα is *de novo* expressed in microglia, astrocytes and oligodendrocytes, highlighting a potential role of this receptor in inflammation and brain repair mechanisms (Meffre et al., 2013). In rat, mPRβ-expressing neurons are detected in the forebrain and the midbrain (Zuloaga et al., 2012). In human, mPR mRNAs are distributed in the forebrain, the hypothalamus and also the hippocampus (Pang et al., 2013).

#### Androgen receptors

In adult rodents, AR mRNA and protein are detected in neurons and glia of the medial amygdala, the preoptic area, the hypothalamus, the cerebellum, and the dentate gyrus of the hippocampus (Commins and Yahr, 1985; Sar et al., 1990; Hajszan et al., 2007; Feng et al., 2010; Pelletier, 2010; Mhaouty-Kodja, 2017). Interestingly, AR protein level is correlated with circulating levels of estradiol and testosterone across the estrous cycle, suggesting an estrogenic regulation of its expression (Feng et al., 2010).

To conclude, across vertebrate species, the distribution of ER, PR, and AR in the brain are evolutionary conserved, especially in the hypothalamus, the preoptic area, and some striatal regions. They are also expressed in neurogenic brain regions, arguing in favor of a role sex steroids in the regulation of NSC activity, neuronal differentiation, and cell survival.

## Role of steroids in the brains of fish, mammals, and birds

In this third part, we will discuss the action of estrogens, progestagens and androgens on constitutive neurogenesis and their effects on CNS following insults such as stroke and traumatic brain injury, with a focus on the impact of sex on brain injury. Then, we will discuss the impact of neurosteroids on behavior in fish, birds, and rodents, focusing only on sexual behavior.

### Constitutive and regenerative neurogenesis

The actions of steroids in the brain are complex and depend of many factors such as the strain, the species, the timing, the concentration, and the rhythm of secretion, as well as the regions studied (Duarte-Guterman et al., 2015; Heberden, 2017). Sex steroids are well-documented for impacting neuronal plasticity including synaptogenesis/spinogenesis such as shown for estrogens (Fester et al., 2012; Brandt et al., 2013; Sellers et al., 2015; Sager et al., 2017), progesterone (Sakamoto et al., 2001b, 2002; Zhao et al., 2011), and testosterone (Manolides and Baloyannis, 1984; Devoogd et al., 1985). They have been also well-characterized for their role on learning/memory processes (Frye and Walf, 2008, 2010; Frye et al., 2008; Phan et al., 2012; Celec et al., 2015; Frick et al., 2015). In addition, estrogens, progestins, and androgens display neuroprotective effects through genomic and non-genomic mechanisms (López-Rodríguez et al., 2015). These neuroprotective effects involved the upregulation of anti-apoptotic factors (i.e., Bcl2) and antioxidant enzymes (i.e., SOD and GPx), as well as the down-regulation of pro-inflammatory cytokines (López-Rodríguez et al., 2015). Due to limited space and to avoid redundancy with recently published reviews (Shahrokhi et al., 2012; Heberden, 2017; McEwen and Milner, 2017), we will mainly focus on the roles of these sex steroids on neurogenesis under homeostatic and regenerative conditions (i.e., stroke, traumatic brain injury).

#### Effects of estrogens

In the brain, estrogens modulate synaptic plasticity, NSC proliferation, newborn neuron migration, differentiation, and survival, as well as neuroprotection (McEwen and Woolley, 1994; McEwen et al., 1995; Azcoitia et al., 2001; McCullough et al., 2003; Murashov et al., 2004; Mukai et al., 2010; Frick et al., 2015).

For instance, 17β-E2 treatment of rat embryonic NSCs increases cell proliferation and neuronal differentiation (Brännvall et al., 2002), and promotes human embryonic NSC differentiation into dopaminergic neurons (Kishi et al., 2005). In addition, ERβ knock-out mice display a thinner cortex at E18.5 due to defects in neuronal migration and increased cell-death arguing for a role of ERβ in newborn neuronal differentiation and survival during the late phase of corticogenesis (Wang et al., 2003).

During adulthood, rat hippocampal cell genesis in female is modulated by ovarian hormone levels and is higher during pro-estrus, when 17β-E2 concentration is elevated (Tanapat et al., 1999, 2005). In the same line of evidence, ovariectomy leads to decreased hippocampal proliferation that is restored by 17β-E2 treatments through ER dependent mechanism as demonstrated by the use of specific ER agonists (Mazzucco et al., 2006). The impact of other estrogens such as estradiol-benzoate, 17α-E2, and estrone have been shown to also increase cell proliferation in ovariectomized rats suggesting that the positive neurogenic effects of estrogens are a general feature of female sex hormones (Frick et al., 2015). In meadow voles (*Microtus pennsylvanicus*), the reproductive status also influences dentate gyrus cell proliferation and/or surviving (higher in inactive females; higher in active males) (Ormerod and Galea, 2001, 2003). However, hippocampal cell proliferation in squirrels (*Sciurus carolinensis*) was not shown to be sexually dimorphic and to be modulated across the reproductive cycle (Lavenex et al., 2000). Furthermore, in striking contrast with most studies, an acute 17β-E2 treatment decreases cell proliferation in the subventricular zone (SVZ) and leads to a decrease in newborn cells in the olfactory bulbs (Brock et al., 2010). In addition, chronic 17β-E2 treatments in zebrafish impair NSC proliferation, cell survival, and the newborn cell migration (Diotel et al., 2013).

Estrogens also display neuroprotective properties and promote neural regeneration following traumatic brain injury and cerebral ischemia by decreasing apoptotic signaling, neuroinflammation, and oxidatative stress and by normalizing glutamate concentrations (Petrone et al., 2015). These neuroprotective effects of estrogens on brain ischemia have been well-established in ovariectomized rodents, and result in a significang decrease in the size of the lesion and in the infarct volume (Gibson et al., 2006; Petrone et al., 2015).

#### Effects of progesterone and allopregnenolone

In the brain, progesterone is known to regulate spinogenesis, synaptogenesis, neuronal survival, and dendritic growth (McEwen and Woolley, 1994; Brinton et al., 2008; Tsutsui, 2008; Zhang et al., 2010; Rossetti et al., 2016). Following cerebral ischemia in rat, progesterone promotes neurogenesis in the SVZ of the lateral ventricles and favors cell survival in the peri-infarct region several days post-stroke (Jiang et al., 2016). Interestingly, numerous studies documented estrogen and progesterone effects on learning and memory across the estrous cycle as reviewed in Duarte-Guterman et al. (2015). Allopregnanolone, a progesterone metabolite considered as one of the most important neuroactive steroid in the CNS, also plays key roles by increasing neurogenesis, neuronal cell survival, and by reducing cell-death in the hippocampus and the midbrain (Charalampopoulos et al., 2008; Zhang et al., 2015; Rossetti et al., 2016). It also exerts major neuroprotective roles in neurodegenerative diseases (Rossetti et al., 2016).

#### Effects of androgens

Concerning androgens, a pioneer study in songbird suggested that testosterone could favor neurogenesis (Louissaint et al., 2002), further corroborated by mice castration studies resulting in decreased hippocampal neurogenesis (Spritzer and Galea, 2007; Heberden, 2017). In songbirds, androgens and estrogens are well-known to induce seasonal-like growth of song nuclei in the adult (Tramontin et al., 2003; Balthazart and Ball, 2016). Additional experiments in rodents document the positive effect of testosterone on SVZ cell proliferation (Tramontin et al., 2003; Farinetti et al., 2015; Balthazart and Ball, 2016), and those of testosterone and dihydrotestosterone on newborn neuron survival (Spritzer and Galea, 2007). In the same line, testosterone increases neurogenesis in wild-type males but not in androgen-insensitive ones (Hamson et al., 2013). This effect is blocked by AR antagonist (flutamide), demonstrating that androgens act through AR and not through ER after conversion into estrogens (Hamson et al., 2013). However, the reality appears more complex as treatment with nandrolone, a synthetic androgen, decreases hippocampal neurogenesis in male and female (Brännvall et al., 2005). Experiments performed in post-ischemic mice established that endogenous androgens do not alter injury-induced neurogenesis, while supra-physiological levels of testosterone and dihydrotestosterone strongly inhibits post-stroke neurogenesis in the dentate gyrus, reducing brain repair (Zhang et al., 2014).

#### Sex difference after brain injuries

Interestingly, clinical and experimental findings are abundant and highlight important sex differences after stroke (Roof and Hall, 2000; Girijala et al., 2017). Clinical studies showed that aging women display worse outcomes following ischemic stroke than men, and higher mortality after hemorrhagic stroke (Sohrabji, 2015). In addition, ischemic stroke model in rodents document that young female have smaller infarcted area than young males (Alkayed et al., 1998), and also exhibit less severe stroke consequences during pro-estrus (high 17β-E2 concentration) than during metestrus (low 17β-E2 concentration) (Liao et al., 2001). However, these studies only correlate between levels of 17β-E2 and stroke severity without taking into consideration other steroid hormone such as progesterone. During aging, mortality is higher in female than in male, and in the case of hemorrhagic stroke, males display greater bleeding and mortality (Sohrabji, 2015). It was also shown that estrogen treatments improve outcomes in young females and males after ischemic and hemorrhagic stroke, while their effects are controversial on aging females during ischemia (Sohrabji, 2015). Other clinical and experimental findings also document the impact of gender and of sex steroids on other CNS insults. For instance, after traumatic brain injury, men displayed greater levels of injury severity as indicated by the Glasgow Coma Scale score, but such an effect is not always found (Slewa-Younan et al., 2004; Davis et al., 2006). Taken together, these data argue in favor of an impact of sex and circulating steroids on the consequences of CNS insults.

**Figure 3:**
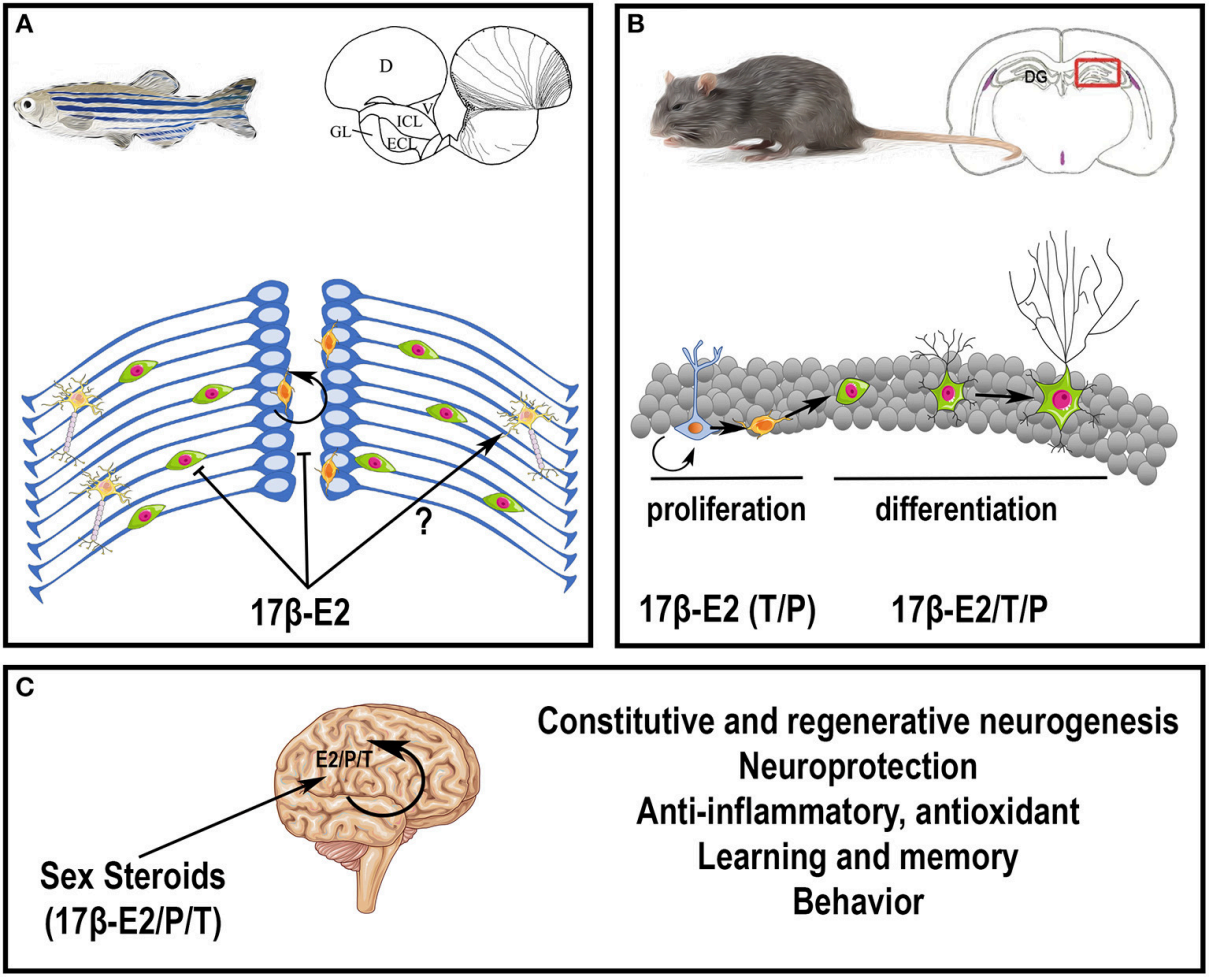
Potential roles of steroids in the brain. **(A)** Effects of 17β-estradiol on neurogenesis in adult zebrafish. At the top, the scheme represents a zebrafish and a transversal brain section through the olfactory bulbs, taken from Menuet et al. (2005). The lines on the right hemisphere correspond to radial glial cells, that behave as neural stem cells. At the bottom, the scheme illustrates neurogenesis events occurring on the zebrafish brain. Radial glial cells (blue) give rise to new neurons (green) that migrate and differentiate into mature and functional neurons (yellow). The orange cells close to the ventricle vicinity are further committed progenitors. It was shown that 17β-estradiol impairs neural progenitor proliferation and new born cell migration; the data obtained for neural differentiation and survival are not clear (Diotel et al., 2013; Pellegrini et al., 2015). **(B)** Effects of sex steroids on neurogenesis in adult rodents. At the top, the scheme represents a rodent and a transversal brain section through the dentate gyrus (DG) of the hippocampus (framed box), where neurogenesis is maintained during adulthood. At the bottom, the scheme illustrates neurogenesis events occurring in the granular cell layer of the dentate gyrus. Radial glial cells (blue light) give rise to neural progenitors (orange) that give birth to new neurons (green with a small neuritic arborescence) that migrate and differentiate into mature and functional neurons (green with a huge neuritic arborescence). In general, 17β-estradiol (17β-E2) favors neural stem cell/progenitor proliferation; to a lesser extent, testosterone (T) and progesterone (P) also exert positive role on proliferation; 17β-estradiol, testosterone and progesterone also promote neuronal migration and differentiation (Heberden, 2017). In addition, these three sex steroids promote new born cell survival in constitutive and regenerative neurogenesis. Note, that the effects of sex steroids on neurogenesis could be dependent of the timing, the concentration, the rhythm of exposure and the regions targeted. For instance, estradiol treatment has been shown to decrease neurogenesis in the SVZ (Brock et al., 2010). **(C)** Peripheral steroids and neurosteroids impact brain functions and homeostasis, by modulating neurogenesis under homeostatic and regenerative conditions, by promoting neuroprotection, learning, and memory, and by exerting anti-inflammatory and antioxidant properties. They also modulate sexual behavior.

In conclusion, sex steroids, peripherally or centrally produced, exert numerous actions on constitutive and regenerative neurogenesis, on brain plasticity, learning, and behaviors (Figure 3). For more details on sex hormone modulation of hippocampal neurogenesis, see (Galea et al., 2006; Frick et al., 2015).

### Sexual behavior

As introduced earlier, sex steroids module in a wide array of behaviors, including aggression, parental behavior, sexual behavior, but also more cognitive aspects, including attention and visual and verbal memory. Although original studies investigated the role of gonadally-produced steroids, it is now clear that the local synthesis of these steroids directly in the central nervous system play a significant role as well. The shift from systemic to local synthesis and regulation of steroid action within target tissues, such as the brain, was called “Balkanization” of the endocrine system (Schmidt et al., 2008). This process would allow the brain to produce specific steroids required for the activation of defined behavior, while leaving other tissues unaffected. The central synthesis of estrogens in the male brain is probably the best example, as high peripheral production of these steroids would have major feminizing effects. Amongst the many behavioral effects of steroids, we chose to discuss in more detail the steroid-dependent modulation of sexual behavior. Indeed, the neuroanatomy and physiology underlying this reproductive behavior have been under study for decades, and sexual behavior provide an excellent model to further investigate the respective role of locally- vs. peripherally- produced sex steroids.

#### Importance of neuroestrogens

The role for aromatase on male sexual behavior was hypothesized over 50 years ago (Clemens, 2013; Södersten, 2013) and a very large number of studies using pharmacological tools have demonstrated that testosterone action on male sexual behavior in various species requires its aromatization in the brain (Christensen and Clemens, 1975; Beyer et al., 1976; Morali et al., 1977; Balthazart and Foidart, 1993; Roselli et al., 2003). These observations were further confirmed by the use of various aromatase knock-out mice (Fisher et al., 1998; Honda et al., 1998; Matsumoto et al., 2003; Bakker et al., 2004). Furthermore, the importance of the central production of estrogens was highlighted by stereotaxic experiments affecting estrogen synthesis within the preoptic area. This region is a key brain center involved the control of male sexual behavior in most vertebrate species and, importantly, contains a very high concentration of aromatase, especially in birds (Balthazart et al., 2004; Perkins and Roselli, 2007; McCarthy, 2011). Numerous studies have shown that preoptic aromatase activity is critical for the activation of male sexual differentiation and male sexual behavior. For example, chronic inhibition of aromatase activity by specific inhibitors delivered to the preoptic area results in the complete suppression of copulatory behavior within a few days (Christensen and Clemens, 1975; Watson and Adkins-Regan, 1989a; Balthazart and Surlemont, 1990; Balthazart et al., 1990). In castrates, the male sexual behavior can be restored by the local administration of testosterone as well as estrogens (Christensen and Clemens, 1974; Watson and Adkins-Regan, 1989b). These results indicate that the expression of male sexual behavior is tied to changes in aromatase concentration and activity in the preoptic area (POA). Interestingly, these change in aromatase expression and male sexual behavior are also linked to an important local neuroplasticity (Aste et al., 1993; Panzica et al., 1998; Charlier et al., 2008). It should be noted that most of these effects were taking place after several days of steroid treatment, clearly suggesting direct genomic effects via the activation of nuclear receptors (Panzica et al., 1998; Scordalakes et al., 2002; Balthazart et al., 2004).

In addition to the long-term effects of estrogens, estrogens also display rapid effects on several aspects of male sexual behavior. For example, research in mouse and Japanese quail showed that acute modulation of estrogen concentrations rapidly affect male sexual behavior (Cornil et al., 2006; Taziaux et al., 2007; Seredynski et al., 2013, 2015). Many of these rapid effects require doses of estradiol that surpass systemic estradiol concentrations in the blood, but local estradiol levels could reach high concentrations quickly, because aromatase and other steroidogenic enzymes can be rapidly regulated in the brain (see below) and are enriched in subcellular compartments such as the presynaptic bouton (Peterson et al., 2005; Saldanha et al., 2011; Cornil et al., 2012). These rapid and local effects of steroids have led to the hypothesis that neuroestrogens act as neurotransmitters (Balthazart and Ball, 2006). Moreover, exposure to sexual stimuli will in turn affect aromatase activity in several regions. Indeed, copulation rapidly and significantly reduced aromatase activity in hypothalamic nuclei and in median preoptic nucleus or tuberal hypothalamus isolated using the Palkovits punch technique (Cornil et al., 2005; de Bournonville et al., 2013). Conversely, exposure to stress upregulates aromatase activity within 5 min in a sex- and brain region-dependent manner in quail (Dickens et al., 2011, 2013), but this increase was counteracted by copulation with a female (Dickens et al., 2012). The mechanisms leading to rapid changes of aromatase activity seem to rely on phosphorylation-dephosphorylation processes but the exact mechanisms are far from being understood (Balthazart et al., 2005, 2006; Charlier et al., 2011a).

It should be noted that the measurements of estrogens do not correlate with aromatase activity in most cases and future work should tackle this discrepancy (Charlier et al., 2011b; Dickens et al., 2014; de Bournonville et al., 2017b). Interestingly, central aromatase is not only important for male sexual behavior but was also shown recently to be involved in female sexual behavior in Japanese quail (de Bournonville et al., 2016, 2017a), see also recent discussion in Cornil (2017). The very high concentration of aromatase in birds make them a very suitable model to investigate its function in modulating sexual behavior but it is likely that rapid change in aromatase activity are also important in mammals and fish, as rapid effects of estrogens were demonstrated on sexual motivation in rat and Goldfish (Cross and Roselli, 1999). However, little is known about the behavioral role of aromatase in fish. As stated above, fish in general possess a very wide distribution of aromatase but the presence of high levels of aromatase, androgen, and estrogen receptors, in brain regions involved in the detection and processing of visual stimuli, suggests that visual processes related to reproduction may be influenced by sex steroids (Gelinas and Callard, 1993, 1997). Testosterone is necessary and sufficient for the enhancement of male approach responses toward a female stimulus to occur and androgen treatments in female goldfish also induce selective approach responses toward female visual stimuli (Thompson et al., 2004). Injections of 17β-E2 had the same behavioral effect as testosterone, and pretreatment with the aromatase inhibitor (fadrozole) 15 min prior to testosterone injections significantly reduced the male's ability to detect and/or orient toward potential mates. Local 17β-E2 synthesis in neural pathways involved in visual processing could therefore sensitize the males to some visual features of females and modulate orientation responses in the context of reproduction (Lord et al., 2009).

#### Importance of neuroprogestins: neuroprogesterone

In most species, the synchrony between ovulation and female sexual behavior is obviously fundamental for efficient reproductive output. Both processes require a sequential elevation of estradiol and progesterone (Mahesh and Brann, 1998; Stephens et al., 2015). Although preliminary observations suggested that progesterone originating from the periphery (Buckingham et al., 1978; Putnam et al., 1991; Mahesh and Brann, 1992), ovariectomized and adrenalectomized rats continue to produce a LH surge in response to estradiol priming, suggesting a role for the central production of progesterone (Ferin et al., 1969; Micevych et al., 2003). This central production of neuroprogesterone is linked to astrocytic activity as astrocytes possess transport proteins (TSPO, StAR) and steroidogenic enzymes (P450ssc, 3βHSD, etc.) required to transform cholesterol into progesterone. Importantly, the synthesis of progesterone in female hypothalamic astrocytes is regulated by estradiol. This estradiol, from gonadal origin will likely affect the transcription and/or activity of multiple transporters and enzymes, including StAR, TSPO, P450ssc, and 3β-HSD, as observed *in vitro* and *in vivo* (Sinchak et al., 2003; Soma et al., 2005; Micevych et al., 2007; Micevych and Sinchak, 2008a; Kuo et al., 2010; Chen et al., 2014). To our knowledge, the role of neuroprogesterone on the display of sexual behavior is currently not known, although the very fast effect of progesterone receptor antisense oligonucleotides on lordosis behavior could suggest a local synthesis of progesterone (Mani et al., 1994). Although the role of neuroprogesterone on female sexual behavior has not been extensively studied, the function of its metabolite allopregnanolone has been investigated in more detail (Frye, 2011).

#### Importance of neuroprogestins: allopregnanolone

Allopregnanolone, also known as 3α-hydroxy-5α-pregnan-20-one (3α,5α-THP), is a neurosteroid produced in several regions of the central nervous system by local conversion of progesterone via 5α-reductase. After paced mating 3α,5α-THP rapidly increases in the midbrain ventral tegmental area (VTA), a dopaminergic region involved in reproductive behaviors of female rodents. The rapidity of the rise in midbrain 3α,5α-THP levels, and independence of ovaries and/or adrenals as sources of steroids, suggest that biosynthesis underlies mating-induced increases (Frye and Vongher, 1999). Paced mating also increases 3α,5α-THP concentrations in other structures of the mesocorticolimbic circuit, such as hippocampus, and cortex (Frye et al., 2013). Central infusions with an inhibitor of TSPO (PK-11195), or with an inhibitor of 3α-HSD within the midbrain VTA significantly attenuated 3α,5α-THP levels of pro-estrus rats and led to a significant reduction in sexual behavior (Frye and Paris, 2011). In order to assess whether central 3α,5α-THP is necessary and sufficient for these effects, pro-estrus rats were infused with 3α,5α-THP subsequent to inhibitor infusions and results indicated a reinstatement of sexual behavior (Frye and Paris, 2011). In hamsters, infusions of a TSPO agonist, to the VTA increased sexual responsiveness and lordosis of cycling, estradiol and progesterone-primed hamsters, compared to vehicle. In contrast, and similarly to what was observed in rat, the TSPO antagonist (PK-11195) injection to the VTA attenuated sexual responsiveness of naturally receptive or estradiol benzoate (EB) + progesterone -primed hamsters compared to vehicle. In parallel, these treatments respectively led to increased and decreased midbrain levels of allopregnenolone (Petralia and Frye, 2005). Future work should define in more detail the precise relationship between localized allopregnanolone synthesis and specific sexual behaviors.

Neurosteroid synthesis is pronounced during early development. Numerous steroidogenic enzymes are expressed at very high levels in the developing avian brain (Goodson et al., 2005; Tsutsui et al., 2006; London and Schlinger, 2007). In rodent brain, most steroidogenic enzymes are typically expressed at much lower levels compared to fish and birds and thus more difficult to quantify but the developmental expression pattern was described for a few steroidogenic enzymes. For example, in rats, brain expression of P450c17, the enzyme responsible for the transformation of pregnenolone to DHEA, is elevated in the embryo and decreases postnatally (Compagnone and Mellon, 2000). Brain 3β-HSD mRNA in rodents is highest during early development (Ibanez et al., 2003). Although steroids from gonadal origin are known to be a significant parameter in sex differentiation in bird, neurosteroids may regulate sexual differentiation in songbird species. In nestling European starlings, neural metabolism of DHEA and 17β-E2 can be greater in males than females at specific ages (Chin et al., 2008; Shah et al., 2011). In zebra finches, 17β-E2 synthesized *de novo* in cultured male brain slices triggers development of an important projection in the song control circuit (Holloway and Clayton, 2001). Female brain slices developed this projection only when co-cultured with male slices, suggesting a sex difference in neural 17β-E2 synthesis during development (Holloway and Clayton, 2001; Schlinger et al., 2001). Progesterone is also synthesized in the brain during development (Zwain and Yen, 1999; Micevych and Sinchak, 2008a,b) and promotes myelination (Koenig et al., 1995; Schumacher et al., 2007).

## Steroids and lipoproteins: from periphery to the brain

### Transport and actions of steroids on the blood-brain barrier

Although neurosteroids play key roles on brain functions, peripheral steroid hormones have the main effects on the CNS, either by crossing the BBB or by targeting the BBB cells, which in turn impact the brain parenchyma by modulating inflammatory and oxidative signals. Some of these possibilities were recently discussed in a review for other hormones called adipokines (Parimisetty et al., 2016). In this part, we will first describe the structure of the BBB in fish, birds, and mammals, then document the transport of steroid through the BBB before discussing the potential roles of steroids on the BBB.

#### Blood-brain barrier in fish, bird, and mammals

The BBB is a specialized layer of cells that controls molecular trafficking between blood and brain, and contributes to the regulation (homeostasis) of the brain microenvironment. In mammalian species, BBB is composed of adjacent endothelial cells bound to each other by tight junctions whose major components are transmembrane and cytoplasmic anchoring protein such as occludin, claudin, and zona occludens (ZO-1). To complete the barrier, endothelial cells are wrapped by pericytes and surrounded by astrocytic cytoplasmic processes called end-feet. This cellular association is important for proper brain homeostasis, neuronal activity but also for protecting nervous tissue pathogens and harmful molecules transported by blood flow. The BBB, by separating the peripheral blood circulation from the brain parenchyma, filters the entrance of many molecules but also the removal of molecules from the brain (Banks, 2012).

**Figure 4:**
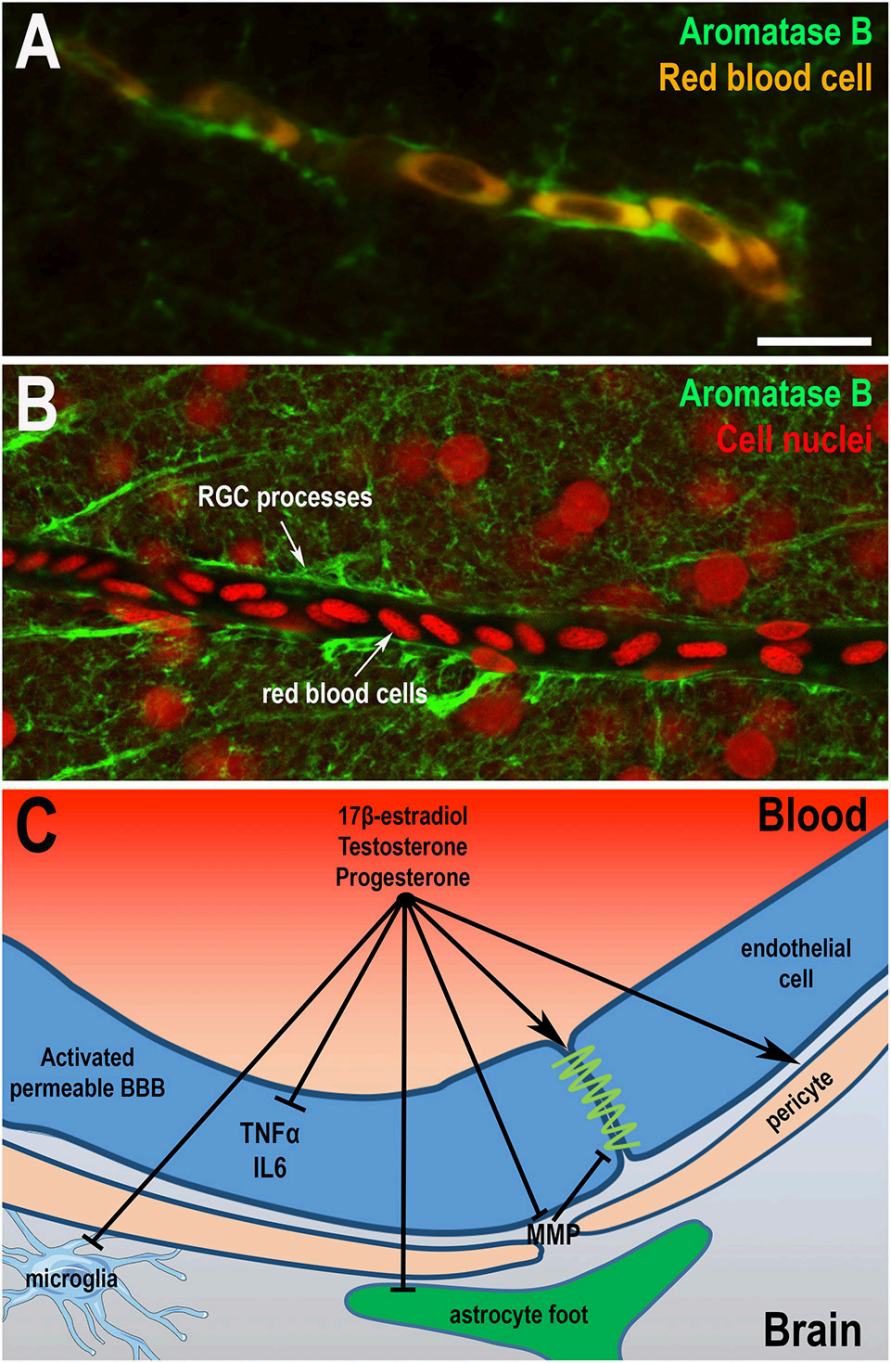
Sex hormones restore blood-brain barrier physiology and integrity. **(A,B)** Aromatase B immunohistochemistry (green) on zebrafish brain highlights that radial glial processes envelop blood vessels as shown by the auto-fluorescence of red blood cells **(A)** or by nuclear staining **(B)**. These data suggest that radial glial cells could endorse the role of astrocytes in the establishment of the BBB, given the absence of astrocytic cell-type in the brain of fish. It also raises the question of the potential role of locally-produced estrogens on the BBB physiology. Scale bar: 7 μm. **(C)** Sex steroids (17β-E2, progesterone, and testosterone) display direct and indirect effects on the BBB through the restauration of thigh junctions, the inhibition of inflammatory cytokine expression and metalloproteinases production, the regulation of pericytes contraction, and consequently the modulation of cerebral blood flow. They also limit reactive gliosis through the inhibition of glial activation under pathological conditions (astrocytes and microglia). Sex steroids participate in the maintenance of a functional BBB, reducing neuroinflammation and promoting neuroprotection.

Very few studies have focused on anatomical and functional organization of the BBB in birds and fish. Electron microscopy performed in adult and embryonic brains of quail and chicken showed that cerebral endothelial cells of both species are bound by tight junctions and that BBB is probably functional very early during embryonic development (Wakai and Hirokawa, 1978; Stewart and Wiley, 1981; Roncali et al., 1986). In embryonic chick, aquaporin 4, a molecular water channels identified in mammalian BBB, was described in astroglia (Nico et al., 2001). In zebrafish, anatomical analysis and transmission electron microscopy revealed that adult endothelial cells display physical barrier properties and express Claudin-5 and ZO-1 proteins (Jeong et al., 2008; Eliceiri et al., 2011; Li et al., 2017). Complete maturation of the zebrafish BBB occurs between 3 and 10 days when expression of Claudin-5 and ZO-1 proteins are detected in cerebral endothelial vascular cells (Jeong et al., 2008; Eliceiri et al., 2011; Fleming et al., 2013; Li et al., 2017). Although some studies have highlighted the presence of stellate astrocytes in some fish (Kawai et al., 2001; Alunni et al., 2005; Strobl-Mazzulla et al., 2010), immunohistochemistry against GFAP or S100β protein has generally failed to demonstrate the presence of star-shaped cells resembling mammalian astrocytes in brain of many fish, including trout, carp, and zebrafish (Arochena et al., 2004; Pellegrini et al., 2005; Grupp et al., 2010; März et al., 2010). These findings raise the possibility that specialized functions of mammalian astrocytes could be supported by the GFAP, BLBP, and S100β-positive RGCs that remains numerous in adults. RNA sequencing analysis performed on goldfish RGC revealed the presence of many receptors and signaling molecules known to be expressed by astrocytes in mammals, a finding that strongly suggests that RGCs share functional similarities with mammalian astrocytes (Grupp et al., 2010; Fleming et al., 2013; Da Fonte et al., 2017). Very interestingly, in the zebrafish brain, blood vessels are wrapped by AroB^+^ processes, in a way similar to astrocytic end-feet in mammals, further reinforcing the idea that RGCs in fish perform some, if not all, functions of astrocytes (Figures 4A,B).

#### Steroid transport through the blood-brain barrier

Steroid hormones are lipophilic and thus may cross the BBB by simple diffusion (Witt and Sandoval, 2014). Hormones can also enter the brain via specific transporters (Banks, 2012). In mammals, the transporters that help the passage from the blood circulation to the brain parenchyma are named influx transporters and include organic anion transporting polypeptides (OATPs), proton-coupled monocarboxylate transporters (MCTs), and peptide transporter. In the case of simple diffusion through the BBB, steroid concentrations will tend to equilibrate between the plasma and the brain. However, most steroid hormones are transported in the blood by albumin, sex hormone-binding globulin, or by corticosteroid-binding globulin, changing their availability and the possibility of passive diffusion through the BBB, as shown in most vertebrates, including fish, birds, and mammals (Hammond, 2011, 2016; Rosner, 2015). In rats, radiolabeled steroids (i.e., progesterone, testosterone, estradiol and corticosterone, and cortisol), intravenously injected have been shown to diffuse through the BBB, but this diffusion was significantly slowed down by the presence of binding globulin (Pardridge and Mietus, 1979). In addition, one must take into account that the concentration of carrier proteins may be different in the peripheral circulation vs. the cerebrospinal fluid (CSF). For example, the albumin concentration is one hundred time less important in the CSF than in the plasma in humans (Alafuzoff et al., 1983), potentially modulating availability and stability of the steroids.

In addition, hormones may be transported back into the blood circulation by efflux transporters. In mammals, these transporters, belonging to the ABC (ATP Binding Cassette) family, play a key role in brain homeostasis and mediate active efflux of many potential toxicants including lipophilic compounds. They include the P-glycoprotein, also known as MDR1, ABCB1), multidrug resistance-associated protein, and breast cancer-related protein (Witt and Sandoval, 2014). Organic ion transporting polypeptides (OATPs) and the organic ion transporters, which belong to the solute-linked carrier (SLC) class, could be involved in uptake and/or efflux (Kusuhara and Sugiyama, 2005). For example, metabolites of estrogens can be transported back in the blood circulation from the brain such as demonstrated in male rats (Sugiyama et al., 2001). The same was also shown in rats for the neuroactive androgenic steroid dehydroepiandrosterone sulfate (DHEAS) that is transported back through a member of the SLC class (Asaba et al., 2000). However, the role of efflux transporter in the regulation of the concentration of steroids in the brain is not well-known and remains controversial.

#### Effects of steroids on the blood-brain barrier

Steroids could act directly or indirectly on the BBB, notably by binding to receptors present in the cells composing the BBB. The effects of glucocorticoids on the BBB are well-described and notably include decrease in inflammation (cytokines, chemokines, metalloproteinases) and increase in protective or reparative effects on tight junctions (Witt and Sandoval, 2014). Sex steroids were also shown to exert protective effects on the BBB, using microvessel endothelial bEnd.3 cells (Na et al., 2015). Considering estrogens, treatment with 17β-E2 restores the BBB integrity and its permeability in a model of stroke or in a lipopolysaccharides-induced inflammation in rodents (Maggioli et al., 2016; Xiao et al., 2017). Of interest, the BBB permeability is increased when circulating estrogens decrease with aging (Bake and Sohrabji, 2004). With respect to androgens, when gonadal testosterone is depleted from the body, glial cells (microglia and astrocytes) are activated and BBB permeability and inflammation are increased. Testosterone treatments restore these parameters (Atallah et al., 2017). Finally, it also seems that progesterone exerts positive effects on the BBB physiology after stroke and TBI (Ishrat et al., 2010; Si et al., 2014). The effects (direct and indirect) of steroids on the BBB is described in Figure 4C.

Interestingly, it was also demonstrated that sex steroids (17β-E2, testosterone, and progesterone) regulate both cerebrovascular tone, endothelial function, oxidative stress, and inflammation as well as brain functions under normal and/or pathological conditions (i.e., middle cerebral artery occlusion: MCAO) (Krause et al., 2011; Gonzales, 2013; Si et al., 2014).

### Lipoproteins in the brain

Lipid metabolism, high density lipoprotein (HDL)-, and low density lipoprotein (LDL)-cholesterol were studied in a wide variety of groups including rodents, lagomorph, birds, and fish (Liu and Wu, 2004). Lipoprotein metabolism, HDL-, and LDL-cholesterol in zebrafish appear similar to humans, making zebrafish an appropriate model for studying lipoproteins, given that zebrafish express the main set of lipid transporters, apolipoproteins, and enzymes involved in lipoprotein metabolism (Fang et al., 2014). However, to our knowledge, except in rodents, not much is known concerning the effects of HDL-cholesterol in the transport of steroids, in the maintenance of the BBB integrity and in neuroprotection. We consequently emphasize these points in this last part.

The brain is a cholesterol-rich organ, accounting for about 25% of the total amount present in humans. In the CNS, cholesterol is mainly synthesized by astrocytes, oligodendrocytes, microglia and to a lesser extent by neurons, where it is essentially present in its unesterified form (Björkhem and Meaney, 2004; Dietschy and Turley, 2004; Zhang and Liu, 2015). Brain cholesterol is involved in myelin sheath genesis, in synaptogenesis, and neurotransmission as well as in neurosteroidogenesis (Mauch et al., 2001; Do Rego et al., 2009; Linetti et al., 2010; Liu et al., 2010; Zhang and Liu, 2015). In adults, sterols are excreted from the brain to the plasma, and cholesterol is converted into 24S-hydroxycholesterol, which is more soluble and may diffuse across the BBB to reach the plasma where it is metabolized to bile acids after being picked up by circulating lipoproteins (Quan et al., 2003; Mahley, 2016). Almost no cholesterol enters the brain from the peripheral circulation. However, small high density lipoprotein (HDL) particles may cross the BBB and transport cholesterol within the brain (Ladu et al., 2000; Koch et al., 2001).

Spherical lipoproteins similar to HDLs have been isolated from the CSF. They are composed of ApoE and ApoJ for the largest ones whereas smaller particles contain ApoAI and ApoAII. Astrocytes are the main producers of both ApoE (with microglial cells) and ApoJ (with neurons and ependymal cells) (Vance and Hayashi, 2010). Different isoforms of ApoE have been described in brain HDL particles (ApoE2, ApoE3, and ApoE4). ApoE is the main cholesterol carrier in the brain and is associated with Alzheimer's disease and other neurodegenerative disorders (Liu et al., 2013). Interestingly, cultured neurons and astrocytes expressing the ApoE4 isoforms display a reduced secretion of cholesterol and phospholipids, as well as a blunted lipid-binding capacity (Mahley, 2016). In addition, estrogens were shown to promote ApoE expression in microglia and astrocytes (Stone et al., 1997). Other brain apolipoproteins (i.e., ApoA-I, Apo-J, and Apo-D) may participate in cholesterol distribution via different receptors including LDL receptor, ABC (ATP-binding cassette receptor) -A1 and -A2 transporters, LDL Receptor-related protein), VLDL receptor, ApoE receptor 2, and megalin (Herz and Bock, 2002; Björkhem and Meaney, 2004).

**Figure 5:**
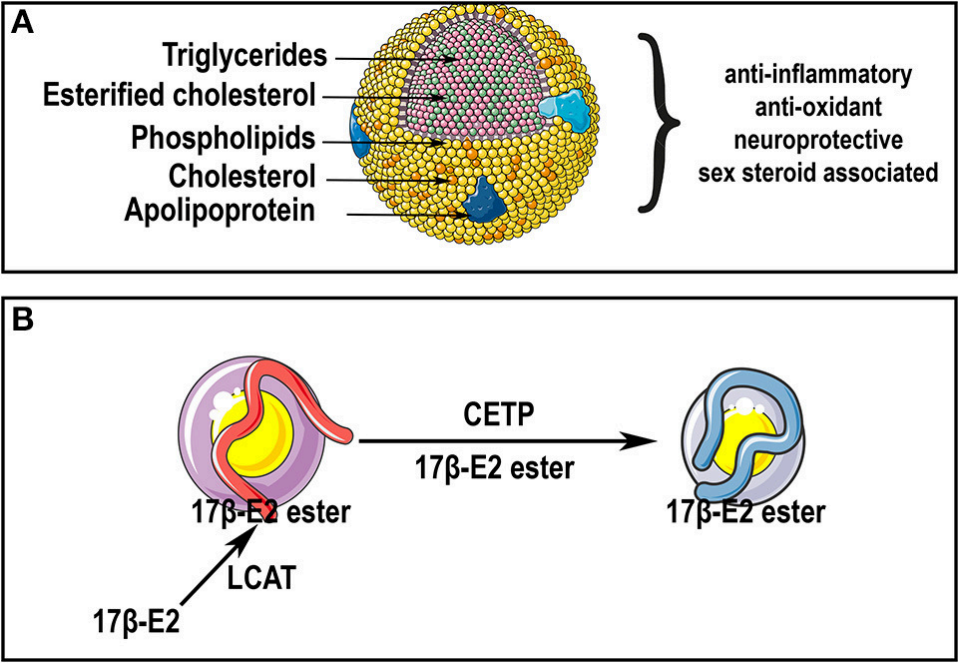
HDL particles and sex steroids. **(A)** Lipoprotein particles display a central core containing cholesterol, cholesterol esters and triglycerides, that is enveloped by free cholesterol, phospholipids, and apolipoproteins. HDL particles display pleiotropic effects partly supported by their anti-inflammatory, anti-oxidant and neuroprotective properties. HDLs have been shown to display metabolic interaction with sex steroids (i.e., androstenediol, estradiol, DHEA, dihydrotestosterone, pregnenolone, and progesterone). **(B)** As suggested by Tikkanen et al. (2002), 17β-E2 could be esterified with fatty acids by lecithin:cholesterol acyltransferase (LCAT) in HDL. Esterified 17β-E2 could be subsequently transferred from HDLs to LDLs by cholesteryl ester transfer protein (CETP). Thus, HDL particles could be a source of cholesterol and steroids in the brain notably after CNS insults, and could be used as a carrier for improving steroids delivery in therapeutically approaches.

In pathological conditions that lead to BBB loss of integrity, such as ischemic stroke, exogenous lipoproteins may enter the cerebral parenchyma and produce different effects. Functional HDL particles (HDLs displaying anti-inflammatory and antioxidant properties, see Figure 5A) are taken up by endothelial cells of the BBB in a thrombo-embolic stroke model of rat. Injection of fluorescent lipoproteins post-stroke are taken up by endothelial cells and astrocytes, but not by neurons (Lapergue et al., 2010). In this model and in the MCAO (middle-cerebral artery occlusion), one intravenous injection of HDLs isolated from healthy subjects reduced the mortality, the infarct volume and the hemorrhagic transformation associated with rtPA (recombinant tissue plasminogen activator) treatment (Lapergue et al., 2013). It is not known whether the cholesterol or steroids contained within these HDL particles are involved in the protective effects. It is indeed possible that either ester of steroids or cholesterol delivery for local steroid synthesis could participate in neurogenesis and post-stroke remodeling. In this line, there is no study showing any beneficial effect of reconstituted HDL particles in CNS disorders. Noteworthy, these HDLs are composed of ApoA-I and phospholipids, but are devoid of cholesterol (Darabi et al., 2016). Ortiz-Munoz et al. recently demonstrated that at the acute phase of stroke, HDL particles are dysfunctional (HDLs displaying defective anti-inflammatory and antioxidant properties) and larger than in controls (Ortiz-Muñoz et al., 2016). These qualitative abnormalities blunted their ability to protect the BBB during acute brain injury. In fact, in addition to their action on reverse transport of cholesterol, HDLs also display anti-oxidant, anti-inflammatory, and anti-protease activities (Tran-Dinh et al., 2013). These pleiotropic effects could be due to different proteins associated with HDL particles such as paraoxonase-1 (PON-1), but also to the lipids that they transport, and to steroids. In type 2 diabetes mellitus conditions (T2DM), HDL anti-inflammatory capacity is impaired due to decreased PON-1 activity (Ebtehaj et al., 2017). Another study investigated the association between plasma cholesterol/lipoproteins and BBB permeability in a condition of CNS inflammation such as multiple sclerosis (Fellows et al., 2015). HDL-Cholesterol was associated with lower levels of BBB injury and low CSF total protein concentration. The authors suggest that the BBB structural integrity is associated with plasma membrane subdomains involved in cholesterol homeostasis. HDL particles are larger in stroke patients than in controls and they display a reduced protective effect for the BBB (Ortiz-Muñoz et al., 2016). Small HDL particles enter the CNS via SR-B1-mediated uptake and transcytosis (Balazs et al., 2004; Vitali et al., 2014). Morphological abnormality of HDLs in brain injury condition like stroke could be responsible for their reduced capacity to cross the blood-cerebrospinal fluid barrier. HDL supplementation could represent an interesting and innovative protective therapy for brain injury.

Interestingly, Leszczynski and Schafer (1991) demonstrated the interaction between lipoproteins and steroid hormones (androstenediol, 17β-E2, DHEA, DHT, pregnenolone, and progesterone), highlighting a new pathway in steroid hormone processing in plasma and/or steroid hormone delivery to cells (Leszczynski and Schafer, 1991). Other reports also suggest that 17β-E2 could be incorporated into HDL particles by lecithin:cholesterol acyltransferase under the form of 17β-E2 ester, that could be in turn transferred into LDL particles by cholesteryl ester transfer protein LDL particles as reviewed in Tikkanen et al. (2002) and Höckerstedt et al. (2004). The Figure 5B illustrates such a transport of 17β-E2 ester in HDLs and LDLs. These data would argue in favor of potential antioxidant, anti-inflammatory, and neuroprotective effects of HDLs implying cholesterol and other steroids (or their esterified forms) associated to lipoprotein particles. Indeed, estrogen derivatives with an unsubstituted A-ring phenolic hydroxyl group confers stronger antioxidant protection to LDL and HDL (Badeau et al., 2005). Such data could suggest a potential therapeutic use of HDL particles in order to improve their neuroprotective properties through their enrichment with steroids.

## Conclusion

In this review, we reported that the brains of fish, birds, and mammals are able to *de novo* synthesize a wide variety of sex steroids demonstrating that neurosteroidogenesis is an evolutionary conserved feature shared by common ancestors. We also emphasize that neurogenic regions are closely related to neurosteroid production. In particular, RGCs, acting as neural progenitors in the brain of adult fish, strongly express Aromatase B and some other steroidogenic enzymes, suggesting that RGCs are true steroidogenic cells. Such an expression of aromatase in RGCs is not restricted to fish as it was also observed in mammals during embryonic development (Martínez-Cerdeño et al., 2006) and in birds after mechanical lesion of the brain (Peterson et al., 2001).

Peripheral, we also highlighted the fact that the brains of fish, birds, and mammals are targeted by peripheral and locally-produced steroids namely estrogens, progestins, and androgens. It appears that nuclear and membrane-associated sex steroid receptors are widely expressed in the brains of vertebrates particularly in neuroendocrine regions (i.e., preoptic area and hypothalamus) as well as in neurogenic niches and neural progenitors, suggesting key roles of steroids in sexual behaviors and neurogenesis. Indeed, we notably depicted the roles of estrogens, progestins and androgens in constitutive and regenerative neurogenesis and in sexual behaviors. Due to their anti-inflammatory, antioxidant, and anti-apoptotic properties, sex steroids also exert neuroprotective properties in the brain, particularly after CNS insults such as stroke and traumatic brain injury. However, the effects of steroids are not trivial to study and understand given that their actions are largely dependent on numerous variables (i.e., the timing, the concentration, the rhythm of exposure,…).

Peripheral steroids may diffuse or cross the BBB through transporters, raising the question of the respective roles of peripherally vs. centrally-produced steroids in brain homeostasis and functions. Peripheral sex steroid and neurosteroids can also act on the BBB permeability by modulating inflammatory and oxidative signals and consequently regulate brain homeostasis.

Of interest, in this review, we also discussed the potential hypothesis that the protective effects of HDL particles on stroke could be partly attributed to their interactions with sex-steroids. Thus, we raised the question of the contribution of cholesterol and sex-steroids transported by lipoproteins in the neuroprotection at the acute phase of stroke. Steroids are considered as good therapeutic candidates for CNS insults (i.e., stroke, traumatic brain injury), aging, and neurodegenerative diseases. It opens a door for therapeutic research through the use of steroids alone, in cocktail, or transported to the injured site by HDLs.

## Author contributions

ND and EP designed the manuscript and supervised the work. ND, EP, TC, OM, CL, DC, VT, JN, and OK participated in the writing of the review. ND and CL made the figures.

### Conflict of interest statement

The authors declare that the research was conducted in the absence of any commercial or financial relationships that could be construed as a potential conflict of interest.

## References

[B1] Adolf B. Chapouton P. Lam C. S. Topp S. Tannhäuser B. Strähle U.. (2006). Conserved and acquired features of adult neurogenesis in the zebrafish telencephalon. Dev. Biol. 295, 278–293. 10.1016/j.ydbio.2006.03.023 16828638

[B2] Alafuzoff I. Adolfsson R. Bucht G. Winblad B. (1983). Albumin and immunoglobulin in plasma and cerebrospinal fluid, and blood-cerebrospinal fluid barrier function in patients with dementia of Alzheimer type and multi-infarct dementia. J. Neurol. Sci. 60, 465–472. 10.1016/0022-510X(83)90157-0 6631444

[B3] Alkayed N. J. Harukuni I. Kimes A. S. London E. D. Traystman R. J. Hurn P. D. (1998). Gender-linked brain injury in experimental stroke. Stroke 29, 159–165: discussion 166. 9445346 10.1161/01.str.29.1.159

[B4] Alunni A. Vaccari S. Torcia S. Meomartini M. E. Nicotra A. Alfei L. (2005). Characterization of glial fibrillary acidic protein and astroglial architecture in the brain of a continuously growing fish, the rainbow trout. Eur. J. Histochem. 49, 157–166. 15967744

[B5] Andersson E. Borg B. Lambert J. G. (1988). Aromatase activity in brain and pituitary of immature and mature Atlantic salmon (*Salmo salar* L.) parr. Gen. Comp. Endocrinol. 72, 394–401. 10.1016/0016-6480(88)90161-X 3240849

[B6] Andreassen T. K. Skjoedt K. Anglade I. Kah O. Korsgaard B. (2003). Molecular cloning, characterisation, and tissue distribution of oestrogen receptor alpha in eelpout (*Zoarces viviparus*). Gen. Comp. Endocrinol. 132, 356–368. 10.1016/S0016-6480(03)00101-1 12849958

[B7] Anglade I. Mazurais D. Douard V. Le Jossic-Corcos C. Mañanos E. L. Michel D.. (1999). Distribution of glutamic acid decarboxylase mRNA in the forebrain of the rainbow trout as studied by *in situ* hybridization. J. Comp. Neurol. 410, 277–289. 10.1002/(SICI)1096-9861(19990726)410:2<277::AID-CNE9>3.0.CO;2-V 10414533

[B8] Anglade I. Pakdel F. Bailhache T. Petit F. Salbert G. Jego P.. (1994). Distribution of estrogen receptor-immunoreactive cells in the brain of the rainbow trout (*Oncorhynchus mykiss*). J. Neuroendocrinol. 6, 573–583. 10.1111/j.1365-2826.1994.tb00621.x 7827628

[B9] Arnold A. P. (1980). Quantitative analysis of sex differences in hormone accumulation in the zebra finch brain: methodological and theoretical issues. J. Comp. Neurol. 189, 421–436. 10.1002/cne.901890302 7372856

[B10] Arochena M. Anadón R. Diaz-Regueira S. M. (2004). Development of vimentin and glial fibrillary acidic protein immunoreactivities in the brain of gray mullet (*Chelon labrosus*), an advanced teleost. J. Comp. Neurol. 469, 413–436. 10.1002/cne.11021 14730591

[B11] Asaba H. Hosoya K. Takanaga H. Ohtsuki S. Tamura E. Takizawa T.. (2000). Blood-brain barrier is involved in the efflux transport of a neuroactive steroid, dehydroepiandrosterone sulfate, via organic anion transporting polypeptide 2. J. Neurochem. 75, 1907–1916. 10.1046/j.1471-4159.2000.0751907.x 11032880

[B12] Aste N. Panzica G. C. Aimar P. Viglietti-Panzica C. Foidart A. Balthazart J. (1993). Implication of testosterone metabolism in the control of the sexually dimorphic nucleus of the quail preoptic area. Brain Res. Bull. 31, 601–611. 10.1016/0361-9230(93)90129-Y 8495382

[B13] Atallah A. Mhaouty-Kodja S. Grange-Messent V. (2017). Chronic depletion of gonadal testosterone leads to blood-brain barrier dysfunction and inflammation in male mice. J. Cereb. Blood Flow Metab. 37, 3161–3175. 10.1177/0271678X16683961 28256950 PMC5584691

[B14] Axelsson J. Mattsson A. Brunström B. Halldin K. (2007). Expression of estrogen receptor-alpha and -beta mRNA in the brain of Japanese quail embryos. Dev. Neurobiol. 67, 1742–1750. 10.1002/dneu.20544 17638389

[B15] Azcoitia I. Sierra A. Veiga S. Honda S. Harada N. Garcia-Segura L. M. (2001). Brain aromatase is neuroprotective. J. Neurobiol. 47, 318–329. 10.1002/neu.1038 11351342

[B16] Badeau M. Adlercreutz H. Kaihovaara P. Tikkanen M. J. (2005). Estrogen A-ring structure and antioxidative effect on lipoproteins. J. Steroid Biochem. Mol. Biol. 96, 271–278. 10.1016/j.jsbmb.2005.04.034 15993048

[B17] Bake S. Sohrabji F. (2004). 17beta-estradiol differentially regulates blood-brain barrier permeability in young and aging female rats. Endocrinology 145, 5471–5475. 10.1210/en.2004-0984 15471968

[B18] Bakker J. Honda S. Harada N. Balthazart J. (2004). Restoration of male sexual behavior by adult exogenous estrogens in male aromatase knockout mice. Horm. Behav. 46, 1–10. 10.1016/j.yhbeh.2004.02.003 15215036

[B19] Balazs Z. Panzenboeck U. Hammer A. Sovic A. Quehenberger O. Malle E.. (2004). Uptake and transport of high-density lipoprotein (HDL) and HDL-associated alpha-tocopherol by an *in vitro* blood-brain barrier model. J. Neurochem. 89, 939–950. 10.1111/j.1471-4159.2004.02373.x 15140193

[B20] Balthazart J. Absil P. Foidart A. Houbart M. Harada N. Ball G. F. (1996). Distribution of aromatase-immunoreactive cells in the forebrain of zebra finches (*Taeniopygia guttata*): implications for the neural action of steroids and nuclear definition in the avian hypothalamus. J. Neurobiol. 31, 129–148. 10.1002/(SICI)1097-4695(199610)31:2<129::AID-NEU1>3.0.CO;2-D 8885196

[B21] Balthazart J. Ball G. F. (1998). New insights into the regulation and function of brain estrogen synthase (aromatase). Trends Neurosci. 21, 243–249. 10.1016/S0166-2236(97)01221-6 9641536

[B22] Balthazart J. Ball G. F. (2006). Is brain estradiol a hormone or a neurotransmitter? Trends Neurosci. 29, 241–249. 10.1016/j.tins.2006.03.004 16580076

[B23] Balthazart J. Ball G. F. (2016). Endocrine and social regulation of adult neurogenesis in songbirds. Front. Neuroendocrinol. 41, 3–22. 10.1016/j.yfrne.2016.03.003 26996818

[B24] Balthazart J. Foidart A. (1993). Brain aromatase and the control of male sexual behavior. J. Steroid Biochem. Mol. Biol. 44, 521–540. 10.1016/0960-0760(93)90256-V 8476766

[B25] Balthazart J. Surlemont C. (1990). Androgen and estrogen action in the preoptic area and activation of copulatory behavior in quail. Physiol. Behav. 48, 599–609. 10.1016/0031-9384(90)90198-D 2082358

[B26] Balthazart J. Baillien M. Ball G. F. (2005). Interactions between kinases and phosphatases in the rapid control of brain aromatase. J. Neuroendocrinol. 17, 553–559. 10.1111/j.1365-2826.2005.01344.x 16101893 PMC2040223

[B27] Balthazart J. Baillien M. Ball G. F. (2006). Rapid control of brain aromatase activity by glutamatergic inputs. Endocrinology 147, 359–366. 10.1210/en.2005-0845 16195408

[B28] Balthazart J. Baillien M. Cornil C. A. Ball G. F. (2004). Preoptic aromatase modulates male sexual behavior: slow and fast mechanisms of action. Physiol. Behav. 83, 247–270. 10.1016/j.physbeh.2004.08.025 15488543

[B29] Balthazart J. Evrard L. Surlemont C. (1990). Effects of the nonsteroidal inhibitor R76713 on testosterone-induced sexual behavior in the Japanese quail (*Coturnix coturnix japonica*). Horm. Behav. 24, 510–531. 10.1016/0018-506X(90)90039-Z 2286366

[B30] Banks W. A. (2012). Brain meets body: the blood-brain barrier as an endocrine interface. Endocrinology 153, 4111–4119. 10.1210/en.2012-1435 22778219 PMC3423627

[B31] Bardet P. L. Horard B. Robinson-Rechavi M. Laudet V. Vanacker J. M. (2002). Characterization of oestrogen receptors in zebrafish (*Danio rerio*). J. Mol. Endocrinol. 28, 153–163. 10.1677/jme.0.0280153 12063182

[B32] Bernard D. J. Bentley G. E. Balthazart J. Turek F. W. Ball G. F. (1999). Androgen receptor, estrogen receptor alpha, and estrogen receptor beta show distinct patterns of expression in forebrain song control nuclei of European starlings. Endocrinology 140, 4633–4643. 10.1210/endo.140.10.7024 10499520

[B33] Beyer C. Moralí G. Naftolin F. Larsson K. Pérez-Palacios G. (1976). Effect of some antiestrogens and aromatase inhibitors on androgen induced sexual behavior in castrated male rats. Horm. Behav. 7, 353–363. 10.1016/0018-506X(76)90040-4 992589

[B34] Björkhem I. Meaney S. (2004). Brain cholesterol: long secret life behind a barrier. Arterioscler. Thromb. Vasc. Biol. 24, 806–815. 10.1161/01.ATV.0000120374.59826.1b 14764421

[B35] Bondesson M. Hao R. Lin C. Y. Williams C. Gustafsson J. Å. (2015). Estrogen receptor signaling during vertebrate development. Biochim. Biophys. Acta 1849, 142–151. 10.1016/j.bbagrm.2014.06.005 24954179 PMC4269570

[B36] Borg B. Andersson E. Mayer I. Lambert J. G. (1989). Aromatase activity in the brain of the three-spined stickleback, *Gasterosteus aculeatus*. III. Effects of castration under different conditions and of replacement with different androgens. Exp. Biol. 48, 149–152. 2721645

[B37] Brandt N. Vierk R. Rune G. M. (2013). Sexual dimorphism in estrogen-induced synaptogenesis in the adult hippocampus. Int. J. Dev. Biol. 57, 351–356. 10.1387/ijdb.120217gr 23873366

[B38] Brännvall K. Bogdanovic N. Korhonen L. Lindholm D. (2005). 19-Nortestosterone influences neural stem cell proliferation and neurogenesis in the rat brain. Eur. J. Neurosci. 21, 871–878. 10.1111/j.1460-9568.2005.03942.x 15787693

[B39] Brännvall K. Korhonen L. Lindholm D. (2002). Estrogen-receptor-dependent regulation of neural stem cell proliferation and differentiation. Mol. Cell. Neurosci. 21, 512–520. 10.1006/mcne.2002.1194 12498791

[B40] Brinton R. D. Thompson R. F. Foy M. R. Baudry M. Wang J. Finch C. E.. (2008). Progesterone receptors: form and function in brain. Front. Neuroendocrinol. 29, 313–339. 10.1016/j.yfrne.2008.02.001 18374402 PMC2398769

[B41] Brock O. Keller M. Veyrac A. Douhard Q. Bakker J. (2010). Short term treatment with estradiol decreases the rate of newly generated cells in the subventricular zone and main olfactory bulb of adult female mice. Neuroscience 166, 368–376. 10.1016/j.neuroscience.2009.12.050 20045446

[B42] Brown R. C. Cascio C. Papadopoulos V. (2000). Pathways of neurosteroid biosynthesis in cell lines from human brain: regulation of dehydroepiandrosterone formation by oxidative stress and beta-amyloid peptide. J. Neurochem. 74, 847–859. 10.1046/j.1471-4159.2000.740847.x 10646538

[B43] Buckingham J. C. Döhler K. D. Wilson C. A. (1978). Activity of the pituitary-adrenocortical system and thyroid gland during the oestrous cycle of the rat. J. Endocrinol. 78, 359–366. 10.1677/joe.0.0780359 213520

[B44] Celec P. Ostatníková D. Hodosy J. (2015). On the effects of testosterone on brain behavioral functions. Front. Neurosci. 9:12. 10.3389/fnins.2015.00012 25741229 PMC4330791

[B45] Celeghin A. Benato F. Pikulkaew S. Rabbane M. G. Colombo L. Dalla Valle L. (2011). The knockdown of the maternal estrogen receptor 2a (esr2a) mRNA affects embryo transcript contents and larval development in zebrafish. Gen. Comp. Endocrinol. 172, 120–129. 10.1016/j.ygcen.2010.12.020 21199655

[B46] Chakraborty T. Shibata Y. Zhou L. Y. Katsu Y. Iguchi T. Nagahama Y. (2011). Differential expression of three estrogen receptor subtype mRNAs in gonads and liver from embryos to adults of the medaka, *Oryzias latipes*. Mol. Cell. Endocrinol. 333, 47–54. 10.1016/j.mce.2010.12.002 21146584

[B47] Charalampopoulos I. Remboutsika E. Margioris A. N. Gravanis A. (2008). Neurosteroids as modulators of neurogenesis and neuronal survival. Trends Endocrinol. Metab. 19, 300–307. 10.1016/j.tem.2008.07.004 18771935

[B48] Charlier T. D. Ball G. F. Balthazart J. (2008). Rapid action on neuroplasticity precedes behavioral activation by testosterone. Horm. Behav. 54, 488–495. 10.1016/j.yhbeh.2008.03.001 18452920 PMC2628423

[B49] Charlier T. D. Harada N. Balthazart J. Cornil C. A. (2011a). Human and quail aromatase activity is rapidly and reversibly inhibited by phosphorylating conditions. Endocrinology 152, 4199–4210. 10.1210/en.2011-0119 21914772 PMC3199011

[B50] Charlier T. D. Newman A. E. Heimovics S. A. Po K. W. Saldanha C. J. Soma K. K. (2011b). Rapid effects of aggressive interactions on aromatase activity and oestradiol in discrete brain regions of wild male white-crowned sparrows. J. Neuroendocrinol. 23, 742–753. 10.1111/j.1365-2826.2011.02170.x 21623961 PMC3135698

[B51] Chen C. F. Wen H. S. Wang Z. P. He F. Zhang J. R. Chen X. Y.. (2010b). Cloning and expression of P450c17-I (17alpha-hydroxylase/17,20-lyase) in brain and ovary during gonad development in *Cynoglossus semilaevis*. Fish Physiol. Biochem. 36, 1001–1012. 10.1007/s10695-009-9378-7 20069358

[B52] Chen C. Kuo J. Wong A. Micevych P. (2014). Estradiol modulates translocator protein (TSPO) and steroid acute regulatory protein (StAR) via protein kinase A (PKA) signaling in hypothalamic astrocytes. Endocrinology 155, 2976–2985. 10.1210/en.2013-1844 24877623 PMC4097996

[B53] Chen S. X. Bogerd J. García-López A. de Jonge H. de Waal P. P. Hong W. S.. (2010a). Molecular cloning and functional characterization of a zebrafish nuclear progesterone receptor. Biol. Reprod. 82, 171–181. 10.1095/biolreprod.109.077644 19741208 PMC2802120

[B54] Chin E. H. Shah A. H. Schmidt K. L. Sheldon L. D. Love O. P. Soma K. K. (2008). Sex differences in DHEA and estradiol during development in a wild songbird: Jugular versus brachial plasma. Horm. Behav. 54, 194–202. 10.1016/j.yhbeh.2008.02.014 18423637

[B55] Choi C. Y. Habibi H. R. (2003). Molecular cloning of estrogen receptor alpha and expression pattern of estrogen receptor subtypes in male and female goldfish. Mol. Cell. Endocrinol. 204, 169–177. 10.1016/S0303-7207(02)00182-X 12850291

[B56] Christensen L. W. Clemens L. G. (1974). Intrahypothalamic implants of testosterone or estradiol and resumption of masculine sexual behavior in long-term castrated male rats. Endocrinology 95, 984–990. 10.1210/endo-95-4-984 4479146

[B57] Christensen L. W. Clemens L. G. (1975). Blockade of testosterone-induced mounting behavior in the male rat with intracranial application of the aromatization inhibitor, androst-1,4,6,-triene-3,17-dione. Endocrinology 97, 1545–1551. 10.1210/endo-97-6-1545 1204576

[B58] Clemens L. G. (2013). The aromatization hypothesis, 1970–1990, in Brain Aromatase, Estrogens, and Behavior, eds Balthazart J. Ball G. F. (New York, NY; Oxford UP), 155–168.

[B59] Commins D. Yahr P. (1985). Autoradiographic localization of estrogen and androgen receptors in the sexually dimorphic area and other regions of the gerbil brain. J. Comp. Neurol. 231, 473–489. 10.1002/cne.902310406 3968250

[B60] Compagnone N. A. Mellon S. H. (2000). Neurosteroids: biosynthesis and function of these novel neuromodulators. Front. Neuroendocrinol. 21, 1–56. 10.1006/frne.1999.0188 10662535

[B61] Cornil C. A. (2017). On the role of brain aromatase in females: why are estrogens produced locally when they are available systemically? J. Comp. Physiol. A Neuroethol. Sens. Neural Behav. Physiol. 204, 31–49. 10.1007/s00359-017-1224-2 29086012 PMC5792365

[B62] Cornil C. A. Dalla C. Papadopoulou-Daifoti Z. Baillien M. Dejace C. Ball G. F.. (2005). Rapid decreases in preoptic aromatase activity and brain monoamine concentrations after engaging in male sexual behavior. Endocrinology 146, 3809–3820. 10.1210/en.2005-0441 15932925 PMC3909742

[B63] Cornil C. A. Leung C. H. Pletcher E. R. Naranjo K. C. Blauman S. J. Saldanha C. J. (2012). Acute and specific modulation of presynaptic aromatization in the vertebrate brain. Endocrinology 153, 2562–2567. 10.1210/en.2011-2159 22508515 PMC3359600

[B64] Cornil C. A. Taziaux M. Baillien M. Ball G. F. Balthazart J. (2006). Rapid effects of aromatase inhibition on male reproductive behaviors in Japanese quail. Horm. Behav. 49, 45–67. 10.1016/j.yhbeh.2005.05.003 15963995 PMC3515763

[B65] Coumailleau P. Pellegrini E. Adrio F. Diotel N. Cano-Nicolau J. Nasri A.. (2015). Aromatase, estrogen receptors and brain development in fish and amphibians. Biochim. Biophys. Acta 1849, 152–162. 10.1016/j.bbagrm.2014.07.002 25038582

[B66] Cross E. Roselli C. E. (1999). 17beta-estradiol rapidly facilitates chemoinvestigation and mounting in castrated male rats. Am. J. Physiol. 276, R1346–R1350. 10233026 10.1152/ajpregu.1999.276.5.R1346

[B67] Da Fonte D. F. Martyniuk C. J. Xing L. Pelin A. Corradi N. Hu W.. (2017). Secretoneurin A regulates neurogenic and inflammatory transcriptional networks in goldfish (*Carassius auratus*) radial glia. Sci. Rep. 7:14930. 10.1038/s41598-017-14930-8 29097753 PMC5668316

[B68] Darabi M. Guillas-Baudouin I. Le Goff W. Chapman M. J. Kontush A. (2016). Therapeutic applications of reconstituted HDL: when structure meets function. Pharmacol. Ther. 157, 28–42. 10.1016/j.pharmthera.2015.10.010 26546991

[B69] Davis D. P. Douglas D. J. Smith W. Sise M. J. Vilke G. M. Holbrook T. L.. (2006). Traumatic brain injury outcomes in pre- and post- menopausal females versus age-matched males. J. Neurotrauma 23, 140–148. 10.1089/neu.2006.23.140 16503798

[B70] de Bournonville C. Ball G. F. Balthazart J. Cornil C. A. (2017a). Rapid changes in brain aromatase activity in the female quail brain following expression of sexual behavior. J. Neuroendocrinol. 29:e12542 10.1111/jne.12542 28990707

[B71] de Bournonville C. Balthazart J. Ball G. F. Cornil C. A. (2016). Non-ovarian aromatization is required to activate female sexual motivation in testosterone-treated ovariectomized quail. Horm. Behav. 83, 45–59. 10.1016/j.yhbeh.2016.05.011 27189762 PMC4916015

[B72] de Bournonville C. Dickens M. J. Ball G. F. Balthazart J. Cornil C. A. (2013). Dynamic changes in brain aromatase activity following sexual interactions in males: where, when and why? Psychoneuroendocrinology 38, 789–799. 10.1016/j.psyneuen.2012.09.001 22999655 PMC3534822

[B73] de Bournonville C. Smolders I. Van Eeckhaut A. Ball G. F. Balthazart J. Cornil C. A. (2017b). Glutamate released in the preoptic area during sexual behavior controls local estrogen synthesis in male quail. Psychoneuroendocrinology 79, 49–58. 10.1016/j.psyneuen.2017.02.002 28259043 PMC5432736

[B74] de Waal P. P. Wang D. S. Nijenhuis W. A. Schulz R. W. Bogerd J. (2008). Functional characterization and expression analysis of the androgen receptor in zebrafish (*Danio rerio*) testis. Reproduction 136, 225–234. 10.1530/REP-08-0055 18469035

[B75] Devoogd T. J. Nixdorf B. Nottebohm F. (1985). Synaptogenesis and changes in synaptic morphology related to acquisition of a new behavior. Brain Res. 329, 304–308. 10.1016/0006-8993(85)90539-6 3978452

[B76] Dickens M. J. Balthazart J. Cornil C. A. (2012). Brain aromatase and circulating corticosterone are rapidly regulated by combined acute stress and sexual interaction in a sex-specific manner. J. Neuroendocrinol. 24, 1322–1334. 10.1111/j.1365-2826.2012.02340.x 22612582 PMC3510384

[B77] Dickens M. J. Cornil C. A. Balthazart J. (2011). Acute stress differentially affects aromatase activity in specific brain nuclei of adult male and female quail. Endocrinology 152, 4242–4251. 10.1210/en.2011-1341 21878510 PMC3199009

[B78] Dickens M. J. Cornil C. A. Balthazart J. (2013). Neurochemical control of rapid stress-induced changes in brain aromatase activity. J. Neuroendocrinol. 25, 329–339. 10.1111/jne.12012 23253172

[B79] Dickens M. J. de Bournonville C. Balthazart J. Cornil C. A. (2014). Relationships between rapid changes in local aromatase activity and estradiol concentrations in male and female quail brain. Horm. Behav. 65, 154–164. 10.1016/j.yhbeh.2013.12.011 24368290 PMC3932376

[B80] Dietschy J. M. Turley S. D. (2004). Thematic review series: brain Lipids. Cholesterol Metabolism in the central nervous system during early development and in the mature animal. J. Lipid Res. 45, 1375–1397. 10.1194/jlr.R400004-JLR200 15254070

[B81] Diotel N. Do Rego J. L. Anglade I. Vaillant C. Pellegrini E. Gueguen M. M.. (2011b). Activity and expression of steroidogenic enzymes in the brain of adult zebrafish. Eur. J. Neurosci. 34, 45–56. 10.1111/j.1460-9568.2011.07731.x 21692878

[B82] Diotel N. Do Rego J. L. Anglade I. Vaillant C. Pellegrini E. Vaudry H.. (2011a). The brain of teleost fish, a source, and a target of sexual steroids. Front. Neurosci. 5:137. 10.3389/fnins.2011.00137 22194715 PMC3242406

[B83] Diotel N. Le Page Y. Mouriec K. Tong S. K. Pellegrini E. Vaillant C.. (2010). Aromatase in the brain of teleost fish: expression, regulation and putative functions. Front. Neuroendocrinol. 31, 172–192. 10.1016/j.yfrne.2010.01.003 20116395

[B84] Diotel N. Servili A. Gueguen M. M. Mironov S. Pellegrini E. Vaillant C.. (2011c). Nuclear progesterone receptors are up-regulated by estrogens in neurons and radial glial progenitors in the brain of zebrafish. PLoS ONE 6:e28375. 10.1371/journal.pone.0028375 22140581 PMC3227669

[B85] Diotel N. Vaillant C. Gabbero C. Mironov S. Fostier A. Gueguen M. M.. (2013). Effects of estradiol in adult neurogenesis and brain repair in zebrafish. Horm. Behav. 63, 193–207. 10.1016/j.yhbeh.2012.04.003 22521210

[B86] Diotel N. Vaillant C. Kah O. Pellegrini E. (2016). Mapping of brain lipid binding protein (Blbp) in the brain of adult zebrafish, co-expression with aromatase B and links with proliferation. Gene Expr. Patterns 20, 42–54. 10.1016/j.gep.2015.11.003 26611722

[B87] Do Rego J. L. Seong J. Y. Burel D. Leprince J. Luu-The V. Tsutsui K.. (2009). Neurosteroid biosynthesis: enzymatic pathways and neuroendocrine regulation by neurotransmitters and neuropeptides. Front. Neuroendocrinol. 30, 259–301. 10.1016/j.yfrne.2009.05.006 19505496

[B88] Duarte-Guterman P. Yagi S. Chow C. Galea L. A. (2015). Hippocampal learning, memory, and neurogenesis: effects of sex and estrogens across the lifespan in adults. Horm. Behav. 74, 37–52. 10.1016/j.yhbeh.2015.05.024 26122299

[B89] Dupont E. Simard J. Luu-The V. Labrie F. Pelletier G. (1994). Localization of 3 beta-hydroxysteroid dehydrogenase in rat brain as studied by *in situ* hybridization. Mol. Cell. Neurosci. 5, 119–123. 10.1006/mcne.1994.1014 8032681

[B90] Ebtehaj S. Gruppen E. G. Parvizi M. U. Tietge U. J. F. Dullaart R. P. F. (2017). The anti-inflammatory function of HDL is impaired in type 2 diabetes: role of hyperglycemia, paraoxonase-1 and low grade inflammation. Cardiovasc. Diabetol. 16, 132. 10.1186/s12933-017-0613-8 29025405 PMC5639738

[B91] Eliceiri B. P. Gonzalez A. M. Baird A. (2011). Zebrafish model of the blood-brain barrier: morphological and permeability studies. Methods Mol. Biol. 686, 371–378. 10.1007/978-1-60761-938-3_18 21082382 PMC4222041

[B92] Escobar S. Felip A. Gueguen M. M. Zanuy S. Carrillo M. Kah O.. (2013). Expression of kisspeptins in the brain and pituitary of the European sea bass (*Dicentrarchus labrax*). J. Comp. Neurol. 521, 933–948. 10.1002/cne.23211 22886357

[B93] Fang L. Liu C. Miller Y. I. (2014). Zebrafish models of dyslipidemia: relevance to atherosclerosis and angiogenesis. Trans. Res. 163, 99–108. 10.1016/j.trsl.2013.09.004 24095954 PMC3946603

[B94] Farinetti A. Tomasi S. Foglio B. Ferraris A. Ponti G. Gotti S.. (2015). Testosterone and estradiol differentially affect cell proliferation in the subventricular zone of young adult gonadectomized male and female rats. Neuroscience 286, 162–170. 10.1016/j.neuroscience.2014.11.050 25481234

[B95] Fellows K. Uher T. Browne R. W. Weinstock-Guttman B. Horakova D. Posova H.. (2015). Protective associations of HDL with blood-brain barrier injury in multiple sclerosis patients. J. Lipid Res. 56, 2010–2018. 10.1194/jlr.M060970 26243484 PMC4583090

[B96] Feng Y. Weijdegård B. Wang T. Egecioglu E. Fernandez-Rodriguez J. Huhtaniemi I.. (2010). Spatiotemporal expression of androgen receptors in the female rat brain during the oestrous cycle and the impact of exogenous androgen administration: a comparison with gonadally intact males. Mol. Cell. Endocrinol. 321, 161–174. 10.1016/j.mce.2010.02.029 20197080

[B97] Ferin M. Tempone A. Zimmering P. E. Van de Wiele R. L. (1969). Effect of antibodies to 17beta-estradiol and progesterone on the estrous cycle of the rat. Endocrinology 85, 1070–1078. 10.1210/endo-85-6-1070 5388410

[B98] Fester L. Prange-Kiel J. Zhou L. Blittersdorf B. V. Bohm J. Jarry H. (2012). Estrogen-regulated synaptogenesis in the hippocampus: sexual dimorphism *in vivo* but not *in vitro*. J. Steroid Biochem. Mol. Biol. 131, 24–29. 10.1016/j.jsbmb.2011.11.010 22138012

[B99] Filby A. L. Tyler C. R. (2005). Molecular characterization of estrogen receptors 1, 2a, and 2b and their tissue and ontogenic expression profiles in fathead minnow (*Pimephales promelas*). Biol. Reprod. 73, 648–662. 10.1095/biolreprod.105.039701 15930325

[B100] Fisher C. R. Graves K. H. Parlow A. F. Simpson E. R. (1998). Characterization of mice deficient in aromatase (ArKO) because of targeted disruption of the cyp19 gene. Proc. Natl. Acad. Sci. U.S.A. 95, 6965–6970. 10.1073/pnas.95.12.6965 9618522 PMC22703

[B101] Fleming A. Diekmann H. Goldsmith P. (2013). Functional characterisation of the maturation of the blood-brain barrier in larval zebrafish. PLoS ONE 8:e77548. 10.1371/journal.pone.0077548 24147021 PMC3797749

[B102] Foidart A. Lakaye B. Grisar T. Ball G. F. Balthazart J. (1999). Estrogen receptor-beta in quail: cloning, tissue expression and neuroanatomical distribution. J. Neurobiol. 40, 327–342. 10.1002/(SICI)1097-4695(19990905)40:3<327::AID-NEU5>3.0.CO;2-L 10440733

[B103] Forlano P. M. Bass A. H. (2005). Seasonal plasticity of brain aromatase mRNA expression in glia: divergence across sex and vocal phenotypes. J. Neurobiol. 65, 37–49. 10.1002/neu.20179 16003720

[B104] Forlano P. M. Deitcher D. L. Bass A. H. (2005). Distribution of estrogen receptor alpha mRNA in the brain and inner ear of a vocal fish with comparisons to sites of aromatase expression. J. Comp. Neurol. 483, 91–113. 10.1002/cne.20397 15672394

[B105] Forlano P. M. Deitcher D. L. Myers D. A. Bass A. H. (2001). Anatomical distribution and cellular basis for high levels of aromatase activity in the brain of teleost fish: aromatase enzyme and mRNA expression identify glia as source. J. Neurosci. 21, 8943–8955. 11698605 10.1523/JNEUROSCI.21-22-08943.2001 PMC6762278

[B106] Forlano P. M. Marchaterre M. Deitcher D. L. Bass A. H. (2010). Distribution of androgen receptor mRNA expression in vocal, auditory, and neuroendocrine circuits in a teleost fish. J. Comp. Neurol. 518, 493–512. 10.1002/cne.22233 20020540 PMC2976675

[B107] Frick K. M. Kim J. Tuscher J. J. Fortress A. M. (2015). Sex steroid hormones matter for learning and memory: estrogenic regulation of hippocampal function in male and female rodents. Learn. Mem. 22, 472–493. 10.1101/lm.037267.114 26286657 PMC4561402

[B108] Froehlicher M. Liedtke A. Groh K. López-Schier H. Neuhauss S. C. Segner H.. (2009). Estrogen receptor subtype beta2 is involved in neuromast development in zebrafish (*Danio rerio*) larvae. Dev. Biol. 330, 32–43. 10.1016/j.ydbio.2009.03.005 19289112

[B109] Frye C. A. (2011). Novel substrates for, and sources of, progestogens for reproduction. J. Neuroendocrinol. 23, 961–973. 10.1111/j.1365-2826.2011.02180.x 21696472 PMC3221002

[B110] Frye C. A. Paris J. J. (2011). Progesterone turnover to its 5alpha-reduced metabolites in the ventral tegmental area of the midbrain is essential for initiating social and affective behavior and progesterone metabolism in female rats. J. Endocrinol. Invest. 34, e188–e199. 10.3275/7334 21060252 PMC3376830

[B111] Frye C. A. Vongher J. M. (1999). 3alpha,5alpha-THP in the midbrain ventral tegmental area of rats and hamsters is increased in exogenous hormonal states associated with estrous cyclicity and sexual receptivity. J. Endocrinol. Invest. 22, 455–464. 10.1007/BF03343590 10435856

[B112] Frye C. A. Walf A. A. (2008). Progesterone enhances performance of aged mice in cortical or hippocampal tasks. Neurosci. Lett. 437, 116–120. 10.1016/j.neulet.2008.04.004 18439758 PMC2573385

[B113] Frye C. A. Walf A. A. (2010). Progesterone enhances learning and memory of aged wildtype and progestin receptor knockout mice. Neurosci. Lett. 472, 38–42. 10.1016/j.neulet.2010.01.051 20117174 PMC3609424

[B114] Frye C. A. Edinger K. Sumida K. (2008). Androgen administration to aged male mice increases anti-anxiety behavior and enhances cognitive performance. Neuropsychopharmacology 33, 1049–1061. 10.1038/sj.npp.1301498 17625503 PMC2572829

[B115] Frye C. A. Koonce C. J. Walf A. A. Rusconi J. C. (2013). Motivated behaviors and levels of 3alpha,5alpha-THP in the midbrain are attenuated by knocking down expression of pregnane xenobiotic receptor in the midbrain ventral tegmental area of proestrous rats. J. Sex. Med. 10, 1692–1706. 10.1111/jsm.12173 23634744 PMC3700579

[B116] Fusani L. Donaldson Z. London S. E. Fuxjager M. J. Schlinger B. A. (2014). Expression of androgen receptor in the brain of a sub-oscine bird with an elaborate courtship display. Neurosci. Lett. 578, 61–65. 10.1016/j.neulet.2014.06.028 24954076 PMC4359618

[B117] Gahr M. (2001). Distribution of sex steroid hormone receptors in the avian brain: functional implications for neural sex differences and sexual behaviors. Microsc. Res. Tech. 55, 1–11. 10.1002/jemt.1151 11596145

[B118] Galea L. A. Spritzer M. D. Barker J. M. Pawluski J. L. (2006). Gonadal hormone modulation of hippocampal neurogenesis in the adult. Hippocampus 16, 225–232. 10.1002/hipo.20154 16411182

[B119] Garcia-Segura L. M. (2008). Aromatase in the brain: not just for reproduction anymore. J. Neuroendocrinol. 20, 705–712. 10.1111/j.1365-2826.2008.01713.x 18601693

[B120] Garcia-Segura L. M. Veiga S. Sierra A. Melcangi R. C. Azcoitia I. (2003). Aromatase: a neuroprotective enzyme. Prog. Neurobiol. 71, 31–41. 10.1016/j.pneurobio.2003.09.005 14611865

[B121] Garcia-Segura L. M. Wozniak A. Azcoitia I. Rodriguez J. R. Hutchison R. E. Hutchison J. B. (1999). Aromatase expression by astrocytes after brain injury: implications for local estrogen formation in brain repair. Neuroscience 89, 567–578. 10.1016/S0306-4522(98)00340-6 10077336

[B122] Gelinas D. Callard G. V. (1993). Immunocytochemical and biochemical evidence for aromatase in neurons of the retina, optic tectum and retinotectal pathways in goldfish. J. Neuroendocrinol. 5, 635–641. 10.1111/j.1365-2826.1993.tb00533.x 8680435

[B123] Gelinas D. Callard G. V. (1997). Immunolocalization of aromatase- and androgen receptor-positive neurons in the goldfish brain. Gen. Comp. Endocrinol. 106, 155–168. 10.1006/gcen.1997.6891 9169111

[B124] Gibson C. L. Gray L. J. Murphy S. P. Bath P. M. (2006). Estrogens and experimental ischemic stroke: a systematic review. J. Cereb. Blood Flow Metab. 26, 1103–1113. 10.1038/sj.jcbfm.9600270 16437060

[B125] Girijala R. L. Sohrabji F. Bush R. L. (2017). Sex differences in stroke: review of current knowledge and evidence. Vasc. Med. 22, 135–145. 10.1177/1358863X16668263 27815349

[B126] Gonzales R. J. (2013). Androgens and the cerebrovasculature: modulation of vascular function during normal and pathophysiological conditions. Pflugers Archiv. 465, 627–642. 10.1007/s00424-013-1267-3 23605065

[B127] Gonzalez A. Piferrer F. (2003). Aromatase activity in the European sea bass (*Dicentrarchus labrax* L.) brain. Distribution and changes in relation to age, sex, and the annual reproductive cycle. Gen. Comp. Endocrinol. 132, 223–230. 10.1016/S0016-6480(03)00086-8 12812769

[B128] González M. Cabrera-Socorro A. Pérez-García C. G. Fraser J. D. López F. J. Alonso R.. (2007). Distribution patterns of estrogen receptor alpha and beta in the human cortex and hippocampus during development and adulthood. J. Comp. Neurol. 503, 790–802. 10.1002/cne.21419 17570500

[B129] Goodson J. L. Saldanha C. J. Hahn T. P. Soma K. K. (2005). Recent advances in behavioral neuroendocrinology: insights from studies on birds. Horm. Behav. 48, 461–473. 10.1016/j.yhbeh.2005.04.005 15896792 PMC2570788

[B130] Gorelick D. A. Watson W. Halpern M. E. (2008). Androgen receptor gene expression in the developing and adult zebrafish brain. Dev. Dyn. 237, 2987–2995. 10.1002/dvdy.21700 18816841

[B131] Goto-Kazeto R. Kight K. E. Zohar Y. Place A. R. Trant J. M. (2004). Localization and expression of aromatase mRNA in adult zebrafish. Gen. Comp. Endocrinol. 139, 72–84. 10.1016/j.ygcen.2004.07.003 15474538

[B132] Griffin L. B. January K. E. Ho K. W. Cotter K. A. Callard G. V. (2013). Morpholino-mediated knockdown of ERalpha, ERbetaa, and ERbetab mRNAs in zebrafish (*Danio rerio*) embryos reveals differential regulation of estrogen-inducible genes. Endocrinology 154, 4158–4169. 10.1210/en.2013-1446 23928376 PMC3800766

[B133] Grupp L. Wolburg H. Mack A. F. (2010). Astroglial structures in the zebrafish brain. J. Comp. Neurol. 518, 4277–4287. 10.1002/cne.22481 20853506

[B134] Guennoun R. Fiddes R. J. Gouézou M. Lombès M. Baulieu E. E. (1995). A key enzyme in the biosynthesis of neurosteroids, 3 beta-hydroxysteroid dehydrogenase/delta 5-delta 4-isomerase (3 beta-HSD), is expressed in rat brain. Brain Res. Mol. Brain Res. 30, 287–300. 10.1016/0169-328X(95)00016-L 7637579

[B135] Guerra-Araiza C. Villamar-Cruz O. González-Arenas A. Chavira R. Camacho-Arroyo I. (2003). Changes in progesterone receptor isoforms content in the rat brain during the oestrous cycle and after oestradiol and progesterone treatments. J. Neuroendocrinol. 15, 984–990. 10.1046/j.1365-2826.2003.01088.x 12969244

[B136] Hagihara K. Hirata S. Osada T. Hirai M. Kato J. (1992a). Distribution of cells containing progesterone receptor mRNA in the female rat di- and telencephalon: an *in situ* hybridization study. Brain Res. Mol. Brain Res. 14, 239–249. 10.1016/0169-328X(92)90179-F 1331652

[B137] Hagihara K. Hirata S. Osada T. Hirai M. Kato J. (1992b). Expression of progesterone receptor in the neonatal rat brain cortex: detection of its mRNA using reverse transcription-polymerase chain reaction. J. Steroid Biochem. Mol. Biol. 41, 637–640. 10.1016/0960-0760(92)90396-Z 1373302

[B138] Hajszan T. Milner T. A. Leranth C. (2007). Sex steroids and the dentate gyrus. Prog. Brain Res. 163, 399–415. 10.1016/S0079-6123(07)63023-4 17765731 PMC1964752

[B139] Halm S. Martínez-Rodríguez G. Rodríguez L. Prat F. Mylonas C. C. Carrillo M.. (2004). Cloning, characterisation, and expression of three oestrogen receptors (ERalpha, ERbeta1 and ERbeta2) in the European sea bass, *Dicentrarchus labrax*. Mol. Cell. Endocrinol. 223, 63–75. 10.1016/j.mce.2004.05.009 15279912

[B140] Hammond G. L. (2011). Diverse roles for sex hormone-binding globulin in reproduction. Biol. Reprod. 85, 431–441. 10.1095/biolreprod.111.092593 21613632 PMC4480437

[B141] Hammond G. L. (2016). Plasma steroid-binding proteins: primary gatekeepers of steroid hormone action. J. Endocrinol. 230, R13–R25. 10.1530/JOE-16-0070 27113851 PMC5064763

[B142] Hamson D. K. Wainwright S. R. Taylor J. R. Jones B. A. Watson N. V. Galea L. A. (2013). Androgens increase survival of adult-born neurons in the dentate gyrus by an androgen receptor-dependent mechanism in male rats. Endocrinology 154, 3294–3304. 10.1210/en.2013-1129 23782943

[B143] Hanna R. N. Zhu Y. (2009). Expression of membrane progestin receptors in zebrafish (*Danio rerio*) oocytes, testis and pituitary. Gen. Comp. Endocrinol. 161, 153–157. 10.1016/j.ygcen.2008.10.006 18957293

[B144] Hanna R. N. Daly S. C. Pang Y. Anglade I. Kah O. Thomas P.. (2010). Characterization and expression of the nuclear progestin receptor in zebrafish gonads and brain. Biol. Reprod. 82, 112–122. 10.1095/biolreprod.109.078527 19741205 PMC2802116

[B145] Harbott L. K. Burmeister S. S. White R. B. Vagell M. Fernald R. D. (2007). Androgen receptors in a cichlid fish, *Astatotilapia burtoni*: structure, localization, and expression levels. J. Comp. Neurol. 504, 57–73. 10.1002/cne.21435 17614300 PMC2743600

[B146] Hawkins M. B. Godwin J. Crews D. Thomas P. (2005). The distributions of the duplicate oestrogen receptors ER-beta a and ER-beta b in the forebrain of the Atlantic croaker (*Micropogonias undulatus*): evidence for subfunctionalization after gene duplication. Proc. Biol. Sci. 272, 633–641. 10.1098/rspb.2004.3008 15817438 PMC1564083

[B147] Hawkins M. B. Thornton J. W. Crews D. Skipper J. K. Dotte A. Thomas P. (2000). Identification of a third distinct estrogen receptor and reclassification of estrogen receptors in teleosts. Proc. Natl. Acad. Sci. U.S.A. 97, 10751–10756. 10.1073/pnas.97.20.10751 11005855 PMC27095

[B148] Heberden C. (2017). Sex steroids and neurogenesis. Biochem. Pharmacol. 141, 56–62. 10.1016/j.bcp.2017.05.019 28571999

[B149] Herz J. Bock H. H. (2002). Lipoprotein receptors in the nervous system. Annu. Rev. Biochem. 71, 405–434. 10.1146/annurev.biochem.71.110601.135342 12045102

[B150] Hiraki T. Nakasone K. Hosono K. Kawabata Y. Nagahama Y. Okubo K. (2014). Neuropeptide B is female-specifically expressed in the telencephalic and preoptic nuclei of the medaka brain. Endocrinology 155, 1021–1032. 10.1210/en.2013-1806 24424038

[B151] Höckerstedt A. Jauhiainen M. Tikkanen M. J. (2004). Lecithin/cholesterol acyltransferase induces estradiol esterification in high-density lipoprotein, increasing its antioxidant potential. J. Clin. Endocrinol. Metab. 89, 5088–5093. 10.1210/jc.2004-0141 15472210

[B152] Hojo Y. Hattori T. A. Enami T. Furukawa A. Suzuki K. Ishii H. T.. (2004). Adult male rat hippocampus synthesizes estradiol from pregnenolone by cytochromes P45017alpha and P450 aromatase localized in neurons. Proc. Natl. Acad. Sci. U.S.A. 101, 865–870. 10.1073/pnas.2630225100 14694190 PMC321772

[B153] Holloway C. C. Clayton D. F. (2001). Estrogen synthesis in the male brain triggers development of the avian song control pathway *in vitro*. Nat. Neurosci. 4, 170–175. 10.1038/84001 11175878

[B154] Honda S. Harada N. Ito S. Takagi Y. Maeda S. (1998). Disruption of sexual behavior in male aromatase-deficient mice lacking exons 1 and 2 of the cyp19 gene. Biochem. Biophys. Res. Commun. 252, 445–449. 10.1006/bbrc.1998.9672 9826549

[B155] Hossain M. S. Larsson A. Scherbak N. Olsson P. E. Orban L. (2008). Zebrafish androgen receptor: isolation, molecular, and biochemical characterization. Biol. Reprod. 78, 361–369. 10.1095/biolreprod.107.062018 17942797

[B156] Ibanez C. Guennoun R. Liere P. Eychenne B. Pianos A. El-Etr M.. (2003). Developmental expression of genes involved in neurosteroidogenesis: 3beta-hydroxysteroid dehydrogenase/delta5-delta4 isomerase in the rat brain. Endocrinology 144, 2902–2911. 10.1210/en.2002-0073 12810545

[B157] Ikeuchi T. Todo T. Kobayashi T. Nagahama Y. (1999). cDNA cloning of a novel androgen receptor subtype. J. Biol. Chem. 274, 25205–25209. 10.1074/jbc.274.36.25205 10464240

[B158] Ikeuchi T. Todo T. Kobayashi T. Nagahama Y. (2002). A novel progestogen receptor subtype in the Japanese eel, *Anguilla japonica*. FEBS Lett. 510, 77–82. 10.1016/S0014-5793(01)03220-3 11755535

[B159] Ishrat T. Sayeed I. Atif F. Hua F. Stein D. G. (2010). Progesterone and allopregnanolone attenuate blood-brain barrier dysfunction following permanent focal ischemia by regulating the expression of matrix metalloproteinases. Exp. Neurol. 226, 183–190. 10.1016/j.expneurol.2010.08.023 20816826 PMC2963070

[B160] Jacobs E. C. Arnold A. P. Campagnoni A. T. (1999). Developmental regulation of the distribution of aromatase- and estrogen-receptor- mRNA-expressing cells in the zebra finch brain. Dev. Neurosci. 21, 453–472. 10.1159/000017413 10640864

[B161] Jayasinghe B. S. Volz D. C. (2012). Aberrant ligand-induced activation of G protein-coupled estrogen receptor 1 (GPER) results in developmental malformations during vertebrate embryogenesis. Toxicol. Sci. 125, 262–273. 10.1093/toxsci/kfr269 21984484

[B162] Jeong J. Y. Kwon H. B. Ahn J. C. Kang D. Kwon S. H. Park J. A.. (2008). Functional and developmental analysis of the blood-brain barrier in zebrafish. Brain Res. Bull. 75, 619–628. 10.1016/j.brainresbull.2007.10.043 18355638

[B163] Jiang C. Zuo F. Wang Y. Lu H. Yang Q. Wang J. (2016). Progesterone changes VEGF and BDNF expression and promotes neurogenesis after ischemic stroke. Mol. Neurobiol. 54, 571–581. 10.1007/s12035-015-9651-y PMC4938789 26746666

[B164] Jorgensen A. Andersen O. Bjerregaard P. Rasmussen L. J. (2007). Identification and characterisation of an androgen receptor from zebrafish *Danio rerio*. Comp. Biochem. Physiol. C. Toxicol. Pharmacol. 146, 561–568. 10.1016/j.cbpc.2007.07.002 17698417

[B165] Kah O. Pellegrini E. Mouriec K. Diotel N. Anglade I. Vaillant C.. (2009). [Oestrogens and neurogenesis: new functions for an old hormone. Lessons from the zebrafish.]. J. Soc. Biol, 203, 29–38. 10.1051/jbio:2009007 19358809

[B166] Kallivretaki E. Eggen R. I. Neuhauss S. C. Kah O. Segner H. (2007). The zebrafish, brain-specific, aromatase cyp19a2 is neither expressed nor distributed in a sexually dimorphic manner during sexual differentiation. Dev. Dyn. 236, 3155–3166. 10.1002/dvdy.21344 17937394

[B167] Katsu Y. Lange A. Miyagawa S. Urushitani H. Tatarazako N. Kawashima Y.. (2013). Cloning, expression and functional characterization of carp, *Cyprinus carpio*, estrogen receptors and their differential activations by estrogens. J. Appl. Toxicol. 33, 41–49. 10.1002/jat.170 21721020

[B168] Kawahara T. Okada H. Yamashita I. (2000). Cloning and expression of genomic and complementary DNAs Encoding an estrogen receptor in the medaka fish, *Oryzias latipes*. Zool. Sci. 17, 643–649. 10.2108/zsj.17.643 18517300

[B169] Kawai H. Arata N. Nakayasu H. (2001). Three-dimensional distribution of astrocytes in zebrafish spinal cord. Glia 36, 406–413. 10.1002/glia.1126 11746776

[B170] Kazeto Y. Goto-Kazeto R. Thomas P. Trant J. M. (2005). Molecular characterization of three forms of putative membrane-bound progestin receptors and their tissue-distribution in channel catfish, *Ictalurus punctatus*. J. Mol. Endocrinol. 34, 781–791. 10.1677/jme.1.01721 15956347

[B171] Kim S. J. Ogasawara K. Park J. G. Takemura A. Nakamura M. (2002). Sequence and expression of androgen receptor and estrogen receptor gene in the sex types of protogynous wrasse, *Halichoeres trimaculatus*. Gen. Comp. Endocrinol. 127, 165–173. 10.1016/S0016-6480(02)00020-5 12383444

[B172] Kimoto T. Tsurugizawa T. Ohta Y. Makino J. Tamura H. Hojo Y.. (2001). Neurosteroid synthesis by cytochrome p450-containing systems localized in the rat brain hippocampal neurons: N-methyl-D-aspartate and calcium-dependent synthesis. Endocrinology 142, 3578–3589. 10.1210/endo.142.8.8327 11459806

[B173] Kishi Y. Takahashi J. Koyanagi M. Morizane A. Okamoto Y. Horiguchi S.. (2005). Estrogen promotes differentiation and survival of dopaminergic neurons derived from human neural stem cells. J. Neurosci. Res. 79, 279–286. 10.1002/jnr.20362 15614791

[B174] Kizil C. Kaslin J. Kroehne V. Brand M. (2012). Adult neurogenesis and brain regeneration in zebrafish. Dev. Neurobiol. 72, 429–461. 10.1002/dneu.20918 21595047

[B175] Koch S. Donarski N. Goetze K. Kreckel M. Stuerenburg H. J. Buhmann C.. (2001). Characterization of four lipoprotein classes in human cerebrospinal fluid. J. Lipid Res. 42 1143–1151. 11441143

[B176] Koenig H. L. Schumacher M. Ferzaz B. Thi A. N. Ressouches A. Guennoun R.. (1995). Progesterone synthesis and myelin formation by Schwann cells. Science 268, 1500–1503. 10.1126/science.7770777 7770777

[B177] Krause D. N. Duckles S. P. Gonzales R. J. (2011). Local oestrogenic/androgenic balance in the cerebral vasculature. Acta Physiol. 203, 181–186. 10.1111/j.1748-1716.2011.02323.x 21535417 PMC3156882

[B178] Kriegstein A. Alvarez-Buylla A. (2009). The glial nature of embryonic and adult neural stem cells. Annu. Rev. Neurosci. 32, 149–184. 10.1146/annurev.neuro.051508.135600 19555289 PMC3086722

[B179] Kuo J. Hamid N. Bondar G. Prossnitz E. R. Micevych P. (2010). Membrane estrogen receptors stimulate intracellular calcium release and progesterone synthesis in hypothalamic astrocytes. J. Neurosci. 30, 12950–12957. 10.1523/JNEUROSCI.1158-10.2010 20881113 PMC2957903

[B180] Kusuhara H. Sugiyama Y. (2005). Active efflux across the blood-brain barrier: role of the solute carrier family. NeuroRx 2, 73–85. 10.1602/neurorx.2.1.73 15717059 PMC539323

[B181] Ladu M. J. Reardon C. Van Eldik L. Fagan A. M. Bu G. Holtzman D.. (2000). Lipoproteins in the central nervous system. Ann. N. Y. Acad. Sci. 903, 167–175. 10.1111/j.1749-6632.2000.tb06365.x 10818504

[B182] Lapergue B. Dang B. Q. Desilles J. P. Ortiz-Munoz G. Delbosc S. Loyau S.. (2013). High-density lipoprotein-based therapy reduces the hemorrhagic complications associated with tissue plasminogen activator treatment in experimental stroke. Stroke 44, 699–707. 10.1161/STROKEAHA.112.667832 23422087

[B183] Lapergue B. Moreno J. A. Dang B. Q. Coutard M. Delbosc S. Raphaeli G.. (2010). Protective effect of high-density lipoprotein-based therapy in a model of embolic stroke. Stroke 41, 1536–1542. 10.1161/STROKEAHA.110.581512 20522814

[B184] Lassiter C. S. Kelley B. Linney E. (2002). Genomic structure and embryonic expression of estrogen receptor beta a (ERbetaa) in zebrafish (*Danio rerio*). Gene 299, 141–151. 10.1016/S0378-1119(02)01050-8 12459262

[B185] Lavenex P. Steele M. A. Jacobs L. F. (2000). The seasonal pattern of cell proliferation and neuron number in the dentate gyrus of wild adult eastern grey squirrels. Eur. J. Neurosci. 12, 643–648. 10.1046/j.1460-9568.2000.00949.x 10712644

[B186] Le Goascogne C. Robel P. Gouézou M. Sananès N. Baulieu E. E. Waterman M. (1987). Neurosteroids: cytochrome P-450scc in rat brain. Science 237, 1212–1215. 10.1126/science.3306919 3306919

[B187] Lephart E. D. (1996). A review of brain aromatase cytochrome P450. Brain Res. Brain Res. Rev. 22, 1–26. 10.1016/0165-0173(96)00002-1 8871783

[B188] Lephart E. D. Simpson E. R. McPhaul M. J. Kilgore M. W. Wilson J. D. Ojeda S. R. (1992). Brain aromatase cytochrome P-450 messenger RNA levels and enzyme activity during prenatal and perinatal development in the rat. Brain Res. Mol. Brain Res. 16, 187–192. 10.1016/0169-328X(92)90224-Y 1337928

[B189] Leszczynski D. E. Schafer R. M. (1991). Metabolic conversion of six steroid hormones by human plasma high-density lipoprotein. Biochim. Biophys. Acta 1083, 18–28. 10.1016/0005-2760(91)90120-7 2031935

[B190] Li X. Schwartz P. E. Rissman E. F. (1997). Distribution of estrogen receptor-beta-like immunoreactivity in rat forebrain. Neuroendocrinology 66, 63–67. 10.1159/000127221 9263202

[B191] Li Y. Chen T. Miao X. Yi X. Wang X. Zhao H.. (2017). Zebrafish: a promising *in vivo* model for assessing the delivery of natural products, fluorescence dyes and drugs across the blood-brain barrier. Pharmacol. Res. 125, 246–257. 10.1016/j.phrs.2017.08.017 28867638

[B192] Liao S. Chen W. Kuo J. Chen C. (2001). Association of serum estrogen level and ischemic neuroprotection in female rats. Neurosci. Lett. 297, 159–162. 10.1016/S0304-3940(00)01704-3 11137752

[B193] Linard B. Anglade I. Corio M. Navas J. M. Pakdel F. Saligaut C.. (1996). Estrogen receptors are expressed in a subset of tyrosine hydroxylase-positive neurons of the anterior preoptic region in the rainbow trout. Neuroendocrinology 63, 156–165. 10.1159/000126952 9053780

[B194] Linetti A. Fratangeli A. Taverna E. Valnegri P. Francolini M. Cappello V.. (2010). Cholesterol reduction impairs exocytosis of synaptic vesicles. J. Cell Sci. 123, 595–605. 10.1242/jcs.060681 20103534

[B195] Liu C. C. Liu C. C. Kanekiyo T. Xu H. Bu G. (2013). Apolipoprotein E and Alzheimer disease: risk, mechanisms and therapy. Nature reviews. Neurology 9, 106–118. 10.1038/nrneurol.2012.263 23296339 PMC3726719

[B196] Liu J. P. Tang Y. Zhou S. Toh B. H. McLean C. Li H. (2010). Cholesterol involvement in the pathogenesis of neurodegenerative diseases. Mol. Cell. Neurosci. 43, 33–42. 10.1016/j.mcn.2009.07.013 19660552

[B197] Liu X. M. Wu F. H. (2004). [Comparison of animal models of hyperlipidemia]. Zhong Xi Yi Jie He Xue Bao 2, 132–134. 10.3736/jcim20040217 15339477

[B198] Liu X. Zhu P. Sham K. W. Yuen J. M. Xie C. Zhang Y.. (2009). Identification of a membrane estrogen receptor in zebrafish with homology to mammalian GPER and its high expression in early germ cells of the testis. Biol. Reprod. 80, 1253–1261. 10.1095/biolreprod.108.070250 19228597

[B199] London S. E. Schlinger B. A. (2007). Steroidogenic enzymes along the ventricular proliferative zone in the developing songbird brain. J. Comp. Neurol. 502, 507–521. 10.1002/cne.21335 17394140

[B200] London S. E. Monks D. A. Wade J. Schlinger B. A. (2006). Widespread capacity for steroid synthesis in the avian brain and song system. Endocrinology 147, 5975–5987. 10.1210/en.2006-0154 16935847 PMC2903432

[B201] London S. E. Remage-Healey L. Schlinger B. A. (2009). Neurosteroid production in the songbird brain: a re-evaluation of core principles. Front. Neuroendocrinol. 30, 302–314. 10.1016/j.yfrne.2009.05.001 19442685 PMC2724309

[B202] López-Rodríguez A. B. Ávila-Rodríguez M. Vega-Vela N. E. Capani F. González J. García-Segura L. M. (2015). Neuroprotection by exogenous estrogenic compounds following traumatic brain injury, in Estrogen Effects on Traumatic Brain Injury - Mechanisms of Neuroprotection and Repair, ed Duncan Kelli A. (Amsterdam: Academic Press Elsevier), 73–90.

[B203] Lord L. D. Bond J. Thompson R. R. (2009). Rapid steroid influences on visually guided sexual behavior in male goldfish. Horm. Behav. 56, 519–526. 10.1016/j.yhbeh.2009.09.002 19751737 PMC3628673

[B204] Louissaint A. Jr. Rao S. Leventhal C. Goldman S. A. (2002). Coordinated interaction of neurogenesis and angiogenesis in the adult songbird brain. Neuron 34, 945–960. 10.1016/S0896-6273(02)00722-5 12086642

[B205] Lubischer J. L. Arnold A. P. (1990). Autoradiographic localization of progestin-concentrating cells in the brain of the zebra finch. J. Comp. Neurol. 291, 450–456. 10.1002/cne.902910310 2298943

[B206] Ma C. H. Dong K. W. Yu K. L. (2000). cDNA cloning and expression of a novel estrogen receptor beta-subtype in goldfish (*Carassius auratus*). Biochim. Biophys. Acta 1490, 145–152. 10.1016/S0167-4781(99)00235-3 10786629

[B207] Maggioli E. McArthur S. Mauro C. Kieswich J. Kusters D. H. Reutelingsperger C. P.. (2016). Estrogen protects the blood-brain barrier from inflammation-induced disruption and increased lymphocyte trafficking. Brain Behav. Immun. 51, 212–222. 10.1016/j.bbi.2015.08.020 26321046

[B208] Mahesh V. B. Brann D. W. (1992). Interaction between ovarian and adrenal steroids in the regulation of gonadotropin secretion. J. Steroid Biochem. Mol. Biol. 41, 495–513. 10.1016/0960-0760(92)90375-S 1562521

[B209] Mahesh V. B. Brann D. W. (1998). Regulation of the preovulatory gonadotropin surge by endogenous steroids. Steroids 63, 616–629. 10.1016/S0039-128X(98)00075-0 9870258

[B210] Mahley R. W. (2016). Central nervous system lipoproteins: ApoE and regulation of cholesterol metabolism. Arterioscler. Thromb. Vasc. Biol. 36, 1305–1315. 10.1161/ATVBAHA.116.307023 27174096 PMC4942259

[B211] Mangiamele L. A. Gomez J. R. Curtis N. J. Thompson R. R. (2017). GPER/GPR30, a membrane estrogen receptor, is expressed in the brain and retina of a social fish (*Carassius auratus*) and colocalizes with isotocin. J. Comp. Neurol. 525, 252–270. 10.1002/cne.24056 27283982 PMC5138143

[B212] Mani S. K. Blaustein J. D. Allen J. M. Law S. W. O'Malley B. W. Clark J. H. (1994). Inhibition of rat sexual behavior by antisense oligonucleotides to the progesterone receptor. Endocrinology 135, 1409–1414. 10.1210/endo.135.4.7925102 7925102

[B213] Manolides L. S. Baloyannis S. J. (1984). Influence of hydrocortisone, progesterone and testosterone on dendritic growth *in vitro*. Acta Otolaryngol. 97, 509–522. 10.3109/00016488409132929 6331706

[B214] Martínez-Cerdeño V. Noctor S. C. Kriegstein A. R. (2006). Estradiol stimulates progenitor cell division in the ventricular and subventricular zones of the embryonic neocortex. Eur. J. Neurosci. 24, 3475–3488. 10.1111/j.1460-9568.2006.05239.x 17229096

[B215] März M. Chapouton P. Diotel N. Vaillant C. Hesl B. Takamiya M.. (2010). Heterogeneity in progenitor cell subtypes in the ventricular zone of the zebrafish adult telencephalon. Glia 58, 870–888. 10.1002/glia.20971 20155821

[B216] Mathieu M. Mensah-Nyagan A. G. Vallarino M. Do-Régo J. L. Beaujean D. Vaudry D.. (2001). Immunohistochemical localization of 3 beta-hydroxysteroid dehydrogenase and 5 alpha-reductase in the brain of the African lungfish *Protopterus annectens*. J. Comp. Neurol. 438, 123–135. 10.1002/cne.1304 11536183

[B217] Matsumoto T. Honda S. Harada N. (2003). Alteration in sex-specific behaviors in male mice lacking the aromatase gene. Neuroendocrinology 77, 416–424. 10.1159/000071313 12845227

[B218] Matsunaga M. Ukena K. Tsutsui K. (2001). Expression and localization of cytochrome P450 17 alpha-hydroxylase/c17,20-lyase in the avian brain. Brain Res. 899, 112–122. 10.1016/S0006-8993(01)02217-X 11311872

[B219] Mauch D. H. Nägler K. Schumacher S. Göritz C. Müller E. C. Otto A.. (2001). CNS synaptogenesis promoted by glia-derived cholesterol. Science 294, 1354–1357. 10.1126/science.294.5545.1354 11701931

[B220] Mazzucco C. A. Lieblich S. E. Bingham B. I. Williamson M. A. Viau V. Galea L. A. (2006). Both estrogen receptor alpha and estrogen receptor beta agonists enhance cell proliferation in the dentate gyrus of adult female rats. Neuroscience 141, 1793–1800. 10.1016/j.neuroscience.2006.05.032 16797852

[B221] McCarthy M. M. (2011). A lumpers versus splitters approach to sexual differentiation of the brain. Front. Neuroendocrinol. 32, 114–123. 10.1016/j.yfrne.2011.01.004 21296103 PMC3085725

[B222] McCullough L. D. Blizzard K. Simpson E. R. Oz O. K. Hurn P. D. (2003). Aromatase cytochrome P450 and extragonadal estrogen play a role in ischemic neuroprotection. J. Neurosci. 23, 8701–8705. 14507969 10.1523/JNEUROSCI.23-25-08701.2003 PMC6740420

[B223] McEwen B. S. Milner T. A. (2017). Understanding the broad influence of sex hormones and sex differences in the brain. J. Neurosci. Res. 95, 24–39. 10.1002/jnr.23809 27870427 PMC5120618

[B224] McEwen B. S. Woolley C. S. (1994). Estradiol and progesterone regulate neuronal structure and synaptic connectivity in adult as well as developing brain. Exp. Gerontol. 29, 431–436. 10.1016/0531-5565(94)90022-1 7925761

[B225] McEwen B. S. Gould E. Orchinik M. Weiland N. G. Woolley C. S. (1995). Oestrogens and the structural and functional plasticity of neurons: implications for memory, ageing and neurodegenerative processes. Ciba Found Symp, 191, 52–66: discussion 66–73. 8582205 10.1002/9780470514757.ch4

[B226] Meffre D. Delespierre B. Gouézou M. Schumacher M. Stein D. G. Guennoun R. (2007). 3beta-Hydroxysteroid dehydrogenase/5-ene-4-ene isomerase mRNA expression in rat brain: effect of pseudopregnancy and traumatic brain injury. J. Steroid Biochem. Mol. Biol. 104, 293–300. 10.1016/j.jsbmb.2007.03.003 17428656

[B227] Meffre D. Labombarda F. Delespierre B. Chastre A. De Nicola A. F. Stein D. G.. (2013). Distribution of membrane progesterone receptor alpha in the male mouse and rat brain and its regulation after traumatic brain injury. Neuroscience 231, 111–124. 10.1016/j.neuroscience.2012.11.039 23211561

[B228] Mellon S. H. Deschepper C. F. (1993). Neurosteroid biosynthesis: genes for adrenal steroidogenic enzymes are expressed in the brain. Brain Res. 629, 283–292. 10.1016/0006-8993(93)91332-M 8111631

[B229] Menuet A. Anglade I. Le Guevel R. Pellegrini E. Pakdel F. Kah O. (2003). Distribution of aromatase mRNA and protein in the brain and pituitary of female rainbow trout: comparison with estrogen receptor alpha. J. Comp. Neurol. 462, 180–193. 10.1002/cne.10726 12794742

[B230] Menuet A. Pellegrini E. Anglade I. Blaise O. Laudet V. Kah O.. (2002). Molecular characterization of three estrogen receptor forms in zebrafish: binding characteristics, transactivation properties, and tissue distributions. Biol. Reprod. 66, 1881–1892. 10.1095/biolreprod66.6.1881 12021076

[B231] Menuet A. Pellegrini E. Brion F. Gueguen M. M. Anglade I. Pakdel F.. (2005). Expression and estrogen-dependent regulation of the zebrafish brain aromatase gene. J. Comp. Neurol. 485, 304–320. 10.1002/cne.20497 15803511

[B232] Mhaouty-Kodja S. (2017). Role of the androgen receptor in the central nervous system. Mol. Cell. Endocrinol. 10.1016/j.mce.2017.08.001 28826929

[B233] Micevych P. E. Chaban V. Ogi J. Dewing P. Lu J. K. Sinchak K. (2007). Estradiol stimulates progesterone synthesis in hypothalamic astrocyte cultures. Endocrinology 148, 782–789. 10.1210/en.2006-0774 17095591

[B234] Micevych P. Sinchak K. (2008a). Synthesis and function of hypothalamic neuroprogesterone in reproduction. Endocrinology 149, 2739–2742. 10.1210/en.2008-0011 18308840 PMC2408819

[B235] Micevych P. Sinchak K. (2008b). Estradiol regulation of progesterone synthesis in the brain. Mol. Cell. Endocrinol. 290, 44–50. 10.1016/j.mce.2008.04.016 18572304 PMC2603025

[B236] Micevych P. Sinchak K. Mills R. H. Tao L. LaPolt P. Lu J. K. (2003). The luteinizing hormone surge is preceded by an estrogen-induced increase of hypothalamic progesterone in ovariectomized and adrenalectomized rats. Neuroendocrinology 78, 29–35. 10.1159/000071703 12869797

[B237] Mitani Y. Kanda S. Akazome Y. Zempo B. Oka Y. (2010). Hypothalamic Kiss1 but not Kiss2 neurons are involved in estrogen feedback in medaka (*Oryzias latipes*). Endocrinology 151, 1751–1759. 10.1210/en.2009-1174 20207833

[B238] Mitra S. W. Hoskin E. Yudkovitz J. Pear L. Wilkinson H. A. Hayashi S.. (2003). Immunolocalization of estrogen receptor beta in the mouse brain: comparison with estrogen receptor alpha. Endocrinology 144, 2055–2067. 10.1210/en.2002-221069 12697714

[B239] Morali G. Larsson K. Beyer C. (1977). Inhibition of testosterone-induced sexual behavior in the castrated male rat by aromatase blockers. Horm. Behav. 9, 203–213. 10.1016/0018-506X(77)90056-3 611073

[B240] Morini M. Peñaranda D. S. Vílchez M. C. Nourizadeh-Lillabadi R. Lafont A. G. Dufour S.. (2017). Nuclear and membrane progestin receptors in the European eel: characterization and expression *in vivo* through spermatogenesis. Comp. Biochem. Physiol. Part A Mol. Integr. Physiol. 207, 79–92. 10.1016/j.cbpa.2017.02.009 28196764

[B241] Mouriec K. Lareyre J. J. Tong S. K. Le Page Y. Vaillant C. Pellegrini E.. (2009). Early regulation of brain aromatase (cyp19a1b) by estrogen receptors during zebrafish development. Dev. Dyn. 238, 2641–2651. 10.1002/dvdy.22069 19718764

[B242] Mouriec K. Pellegrini E. Anglade I. Menuet A. Adrio F. Thieulant M. L.. (2008). Synthesis of estrogens in progenitor cells of adult fish brain: evolutive novelty or exaggeration of a more general mechanism implicating estrogens in neurogenesis? Brain Res. Bull. 75, 274–280. 10.1016/j.brainresbull.2007.10.030 18331884

[B243] Mourot B. Nguyen T. Fostier A. Bobe J. (2006). Two unrelated putative membrane-bound progestin receptors, progesterone membrane receptor component 1 (PGMRC1) and membrane progestin receptor (mPR) beta, are expressed in the rainbow trout oocyte and exhibit similar ovarian expression patterns. Reprod. Biol. Endocrinol. 4:6. 10.1186/1477-7827-4-6 16457725 PMC1373632

[B244] Mukai H. Kimoto T. Hojo Y. Kawato S. Murakami G. Higo S.. (2010). Modulation of synaptic plasticity by brain estrogen in the hippocampus. Biochim. Biophys. Acta 1800, 1030–1044. 10.1016/j.bbagen.2009.11.002 19909788

[B245] Muñoz-Cueto J. A. Burzawa-Gérard E. Kah O. Valotaire Y. Pakdel F. (1999). Cloning and sequencing of the gilthead sea bream estrogen receptor cDNA. DNA Seq. 10, 75–84. 10376207 10.3109/10425179909008421

[B246] Murashov A. K. Pak E. S. Hendricks W. A. Tatko L. M. (2004). 17beta-Estradiol enhances neuronal differentiation of mouse embryonic stem cells. FEBS Lett. 569, 165–168. 10.1016/j.febslet.2004.05.042 15225627

[B247] Na W. Lee J. Y. Kim W. S. Yune T. Y. Ju B. G. (2015). 17beta-estradiol ameliorates tight junction disruption via repression of MMP transcription. Mol. Endocrinol. 29, 1347–1361. 10.1210/ME.2015-1124 26168035 PMC5414681

[B248] Nagler J. J. Cavileer T. Sullivan J. Cyr D. G. Rexroad C. III. (2007). The complete nuclear estrogen receptor family in the rainbow trout: discovery of the novel ERalpha2 and both ERbeta isoforms. Gene 392, 164–173. 10.1016/j.gene.2006.12.030 17307310 PMC1868691

[B249] Navas J. M. Anglade I. Bailhache T. Pakdel F. Breton B. Jego P.. (1995). Do gonadotrophin-releasing hormone neurons express estrogen receptors in the rainbow trout? A double immunohistochemical study. J. Comp. Neurol. 363, 461–474. 10.1002/cne.903630309 8847411

[B250] Nico B. Frigeri A. Nicchia G. P. Quondamatteo F. Herken R. Errede M.. (2001). Role of aquaporin-4 water channel in the development and integrity of the blood-brain barrier. J. Cell Sci. 114, 1297–1307. 11256996 10.1242/jcs.114.7.1297

[B251] Noctor S. C. Flint A. C. Weissman T. A. Dammerman R. S. Kriegstein A. R. (2001). Neurons derived from radial glial cells establish radial units in neocortex. Nature 409, 714–720. 10.1038/35055553 11217860

[B252] Okada M. Makino A. Nakajima M. Okuyama S. Furukawa S. Furukawa Y. (2010). Estrogen stimulates proliferation and differentiation of neural stem/progenitor cells through different signal transduction pathways. Int. J. Mol. Sci. 11, 4114–4123. 10.3390/ijms11104114 21152324 PMC2996786

[B253] Olsson P. E. Berg A. H. von Hofsten J. Grahn B. Hellqvist A. Larsson A.. (2005). Molecular cloning and characterization of a nuclear androgen receptor activated by 11-ketotestosterone. Reprod. Biol. Endocrinol. 3:37. 10.1186/1477-7827-3-37 16107211 PMC1192819

[B254] Ormerod B. K. Galea L. A. (2001). Reproductive status influences cell proliferation and cell survival in the dentate gyrus of adult female meadow voles: a possible regulatory role for estradiol. Neuroscience 102, 369–379. 10.1016/S0306-4522(00)00474-7 11166123

[B255] Ormerod B. K. Galea L. A. (2003). Reproductive status influences the survival of new cells in the dentate gyrus of adult male meadow voles. Neurosci. Lett. 346, 25–28. 10.1016/S0304-3940(03)00546-9 12850539

[B256] Ortiz-Muñoz G. Couret D. Lapergue B. Bruckert E. Meseguer E. Amarenco P.. (2016). Dysfunctional HDL in acute stroke. Atherosclerosis 253, 75–80. 10.1016/j.atherosclerosis.2016.08.035 27591364

[B257] Pakdel F. Le Gac F. Le Goff P. Valotaire Y. (1990). Full-length sequence and *in vitro* expression of rainbow trout estrogen receptor cDN. Mol. Cell. Endocrinol. 71, 195–204. 10.1016/0303-7207(90)90025-4 2210031

[B258] Pang Y. Thomas P. (2009). Involvement of estradiol-17beta and its membrane receptor, G protein coupled receptor 30 (GPR30) in regulation of oocyte maturation in zebrafish, *Danio rario*. Gen. Comp. Endocrinol. 161, 58–61. 10.1016/j.ygcen.2008.10.003 18952087 PMC2754812

[B259] Pang Y. Dong J. Thomas P. (2008). Estrogen signaling characteristics of Atlantic croaker G protein-coupled receptor 30 (GPR30) and evidence it is involved in maintenance of oocyte meiotic arrest. Endocrinology 149, 3410–3426. 10.1210/en.2007-1663 18420744 PMC2453078

[B260] Pang Y. Dong J. Thomas P. (2013). Characterization, neurosteroid binding and brain distribution of human membrane progesterone receptors delta and {epsilon} (mPRdelta and mPR{epsilon}) and mPRdelta involvement in neurosteroid inhibition of apoptosis. Endocrinology 154, 283–295. 10.1210/en.2012-1772 23161870 PMC3529379

[B261] Panzica G. C. Castagna C. Viglietti-Panzica C. Russo C. Tlemcani O. Balthazart J. (1998). Organizational effects of estrogens on brain vasotocin and sexual behavior in quail. J. Neurobiol. 37, 684–699. 10.1002/(SICI)1097-4695(199812)37:4<684::AID-NEU15>3.0.CO;2-U 9858268

[B262] Pardridge W. M. Mietus L. J. (1979). Transport of steroid hormones through the rat blood-brain barrier. Primary role of albumin-bound hormone. J. Clin. Investig. 64, 145–154. 10.1172/JCI109433 447850 PMC372100

[B263] Parimisetty A. Dorsemans A. C. Awada R. Ravanan P. Diotel N. Lefebvre d'Hellencourt C. (2016). Secret talk between adipose tissue and central nervous system via secreted factors-an emerging frontier in the neurodegenerative research. J. Neuroinflammation 13, 67. 10.1186/s12974-016-0530-x 27012931 PMC4806498

[B264] Pasmanik M. Callard G. V. (1985). Aromatase and 5 alpha-reductase in the teleost brain, spinal cord, and pituitary gland. Gen. Comp. Endocrinol. 60, 244–251. 10.1016/0016-6480(85)90320-X 4065533

[B265] Pasmanik M. Callard G. V. (1988). Changes in brain aromatase and 5 alpha-reductase activities correlate significantly with seasonal reproductive cycles in goldfish (*Carassius auratus*). Endocrinology 122, 1349–1356. 10.1210/endo-122-4-1349 3345716

[B266] Pawlisch B. A. Remage-Healey L. (2015). Neuroestrogen signaling in the songbird auditory cortex propagates into a sensorimotor network via an ‘interface’ nucleus. Neuroscience 284, 522–535. 10.1016/j.neuroscience.2014.10.023 25453773 PMC4268063

[B267] Pellegrini E. Coumailleau P. Kah O. Diotel N. (2015). Aromatase and estrogens: involvement in constitutive and regenerative neurogenesis in adult zebrafish, in Estrogen Effects on Traumatic Brain Injury - Mechanisms of Neuroprotection and Repair, ed Duncan Kelli A. (Amsterdam: Academic Press Elsevier), 51–71.

[B268] Pellegrini E. Menuet A. Lethimonier C. Adrio F. Gueguen M. M. Tascon C.. (2005). Relationships between aromatase and estrogen receptors in the brain of teleost fish. Gen. Comp. Endocrinol. 142, 60–66. 10.1016/j.ygcen.2004.12.003 15862549

[B269] Pellegrini E. Mouriec K. Anglade I. Menuet A. Le Page Y. Gueguen M. M.. (2007). Identification of aromatase-positive radial glial cells as progenitor cells in the ventricular layer of the forebrain in zebrafish. J. Comp. Neurol. 501, 150–167. 10.1002/cne.21222 17206614

[B270] Pellegrini E. Vaillant C. Diotel N. Benquet P. Brion F. Kah O. (2013). Expression, regulation and potential functions of aromatase in radial glial cells of the fish brain, in Brain Aromatase, Estrogens, and Behavior, eds Balthazart Ball J. Gregory F. (Oxford; New York, NY: University Press), 2013.

[B271] Pelletier G. (2010). Steroidogenic enzymes in the brain: morphological aspects. Prog. Brain Res. 181, 193–207. 10.1016/S0079-6123(08)81011-4 20478439

[B272] Perkins A. Roselli C. E. (2007). The ram as a model for behavioral neuroendocrinology. Horm. Behav. 52, 70–77. 10.1016/j.yhbeh.2007.03.016 17482616 PMC2150593

[B273] Petersen S. L. Intlekofer K. A. Moura-Conlon P. J. Brewer D. N. Del Pino Sans J. Lopez J. A. (2013). Novel progesterone receptors: neural localization and possible functions. Front. Neurosci. 7:164. 10.3389/fnins.2013.00164 24065878 PMC3776953

[B274] Peterson R. S. Lee D. W. Fernando G. Schlinger B. A. (2004). Radial glia express aromatase in the injured zebra finch brain. J. Comp. Neurol. 475, 261–269. 10.1002/cne.20157 15211466

[B275] Peterson R. S. Saldanha C. J. Schlinger B. A. (2001). Rapid upregulation of aromatase mRNA and protein following neural injury in the zebra finch (*Taeniopygia guttata*). J. Neuroendocrinol. 13, 317–323. 10.1046/j.1365-2826.2001.00647.x 11264718

[B276] Peterson R. S. Yarram L. Schlinger B. A. Saldanha C. J. (2005). Aromatase is pre-synaptic and sexually dimorphic in the adult zebra finch brain. Proc. Biol. Sci. 272, 2089–2096. 10.1098/rspb.2005.3181 16191621 PMC1559905

[B277] Petralia S. M. Frye C. A. (2005). In the ventral tegmental area picrotoxin blocks FGIN 1-27-induced increases in sexual behavior of rats and hamsters. Psychopharmacology 178, 174–182. 10.1007/s00213-004-2001-9 15338106

[B278] Petrone A. B. Rudy C. C. Barr T. L. Simpkins J. W. Reed M. N. (2015). Neuroprotective effects of estrogen following neural injury, in Estrogen Effects on Traumatic Brain Injury - Mechanisms of Neuroprotection and Repair, ed Duncan Kelli A. (Amsterdam: Academic Press Elsevier), 91–111.

[B279] Phan A. Gabor C. S. Favaro K. J. Kaschack S. Armstrong J. N. MacLusky N. J.. (2012). Low doses of 17beta-estradiol rapidly improve learning and increase hippocampal dendritic spines. Neuropsychopharmacology 37, 2299–2309. 10.1038/npp.2012.82 22669167 PMC3422494

[B280] Pouso P. Quintana L. Bolatto C. Silva A. C. (2010). Brain androgen receptor expression correlates with seasonal changes in the behavior of a weakly electric fish, *Brachyhypopomus gauderio*. Horm. Behav. 58, 729–736. 10.1016/j.yhbeh.2010.07.005 20688071

[B281] Putnam C. D. Brann D. W. Mahesh V. B. (1991). Acute activation of the adrenocorticotropin-adrenal axis: effect on gonadotropin and prolactin secretion in the female rat. Endocrinology 128, 2558–2566. 10.1210/endo-128-5-2558 1850356

[B282] Quan G. Xie C. Dietschy J. M. Turley S. D. (2003). Ontogenesis and regulation of cholesterol metabolism in the central nervous system of the mouse. Brain Res. Dev. Brain Res. 146, 87–98. 10.1016/j.devbrainres.2003.09.015 14643015

[B283] Ravi V. Venkatesh B. (2008). Rapidly evolving fish genomes and teleost diversity. Curr. Opin. Genet. Dev. 18, 544–550. 10.1016/j.gde.2008.11.001 19095434

[B284] Roncali L. Nico B. Ribatti D. Bertossi M. Mancini L. (1986). Microscopical and ultrastructural investigations on the development of the blood-brain barrier in the chick embryo optic tectum. Acta Neuropathol. 70, 193–201. 10.1007/BF00686072 3766122

[B285] Roof R. L. Hall E. D. (2000). Gender differences in acute CNS trauma and stroke: neuroprotective effects of estrogen and progesterone. J. Neurotrauma 17, 367–388. 10.1089/neu.2000.17.367 10833057

[B286] Roselli C. E. Cross E. Poonyagariyagorn H. K. Stadelman H. L. (2003). Role of aromatization in anticipatory and consummatory aspects of sexual behavior in male rats. Horm. Behav. 44, 146–151. 10.1016/S0018-506X(03)00123-5 13129487

[B287] Roselli C. F. (2007). Brain aromatase: roles in reproduction and neuroprotection. J. Steroid Biochem. Mol. Biol. 106, 143–150. 10.1016/j.jsbmb.2007.05.014 17643294 PMC2002474

[B288] Rosner W. (2015). Free estradiol and sex hormone-binding globulin. Steroids 99, 113–116. 10.1016/j.steroids.2014.08.005 25453337

[B289] Rossetti M. F. Cambiasso M. J. Holschbach M. A. Cabrera R. (2016). Oestrogens and progestagens: synthesis and action in the brain. J. Neuroendocrinol. 28. 10.1111/jne.12402 27306650

[B290] Rothenaigner I. Krecsmarik M. Hayes J. A. Bahn B. Lepier A. Fortin G.. (2011). Clonal analysis by distinct viral vectors identifies bona fide neural stem cells in the adult zebrafish telencephalon and characterizes their division properties and fate. Development 138, 1459–1469. 10.1242/dev.058156 21367818

[B291] Roy S. R. Wang J. Rana M. R. Nakashima M. Tokumoto T. (2017). Characterization of membrane progestin receptor alpha (mPRalpha) of the medaka and role in the induction of oocyte maturation. Biomed. Res. 38, 79–87. 10.2220/biomedres.38.79 28239035

[B292] Sabo-Attwood T. Kroll K. J. Denslow N. D. (2004). Differential expression of largemouth bass (*Micropterus salmoides*) estrogen receptor isotypes alpha, beta, and gamma by estradiol. Mol. Cell. Endocrinol. 218, 107–118. 10.1016/j.mce.2003.12.007 15130515

[B293] Sager T. Kashon M. Krajnak K. (2017). Estrogen and environmental enrichment differentially affect neurogenesis, dendritic spine immunolabeling and synaptogenesis in the hippocampus of young and reproductively senescent female rats. Neuroendocrinology. 10.1159/000479699 28738393

[B294] Sakamoto H. Ukena K. Tsutsui K. (2001a). Activity and localization of 3beta-hydroxysteroid dehydrogenase/ Delta5-Delta4-isomerase in the zebrafish central nervous system. J. Comp. Neurol. 439, 291–305. 10.1002/cne.1351 11596055

[B295] Sakamoto H. Ukena K. Tsutsui K. (2001b). Effects of progesterone synthesized *de novo* in the developing Purkinje cell on its dendritic growth and synaptogenesis. J. Neurosci. 21, 6221–6232. 11487645 10.1523/JNEUROSCI.21-16-06221.2001 PMC6763166

[B296] Sakamoto H. Ukena K. Tsutsui K. (2002). Dendritic spine formation in response to progesterone synthesized de novo in the developing Purkinje cell in rats. Neurosci. Lett. 322, 111–115. 10.1016/S0304-3940(02)00077-0 11958856

[B297] Salbert G. Bonnec G. Le Goff P. Boujard D. Valotaire Y. Jego P. (1991). Localization of the estradiol receptor mRNA in the forebrain of the rainbow trout. Mol. Cell. Endocrinol. 76, 173–180. 10.1016/0303-7207(91)90271-S 1820972

[B298] Saldanha C. J. Remage-Healey L. Schlinger B. A. (2011). Synaptocrine signaling: steroid synthesis and action at the synapse. Endocr. Rev. 32, 532–549. 10.1210/er.2011-0004 21622487 PMC3369574

[B299] Sar M. Lubahn D. B. French F. S. Wilson E. M. (1990). Immunohistochemical localization of the androgen receptor in rat and human tissues. Endocrinology 127, 3180–3186. 10.1210/endo-127-6-3180 1701137

[B300] Sasano H. Takashashi K. Satoh F. Nagura H. Harada N. (1998). Aromatase in the human central nervous system. Clin. Endocrinol. 48, 325–329. 10.1046/j.1365-2265.1998.00390.x 9578823

[B301] Schlinger B. A. (1997). The activity and expression of aromatase in songbirds. Brain Res. Bull. 44, 359–364. 10.1016/S0361-9230(97)00215-3 9370200

[B302] Schlinger B. A. Remage-Healey L. (2012). Neurosteroidogenesis: insights from studies of songbirds. J. Neuroendocrinol. 24, 16–21. 10.1111/j.1365-2826.2011.02150.x 21535249 PMC3197953

[B303] Schlinger B. A. Pradhan D. S. Soma K. K. (2008). 3beta-HSD activates DHEA in the songbird brain. Neurochem. Int. 52, 611–620. 10.1016/j.neuint.2007.05.003 17643555 PMC2441539

[B304] Schlinger B. A. Soma K. K. London S. E. (2001). Neurosteroids and brain sexual differentiation. Trends Neurosci. 24, 429–431. 10.1016/S0166-2236(00)01855-5 11476868

[B305] Schmidt K. L. Pradhan D. S. Shah A. H. Charlier T. D. Chin E. H. Soma K. K. (2008). Neurosteroids, immunosteroids, and the Balkanization of endocrinology. Gen. Comp. Endocrinol. 157, 266–274. 10.1016/j.ygcen.2008.03.025 18486132

[B306] Schumacher M. Guennoun R. Stein D. G. De Nicola A. F. (2007). Progesterone: therapeutic opportunities for neuroprotection and myelin repair. Pharmacol. Ther. 116, 77–106. 10.1016/j.pharmthera.2007.06.001 17659348

[B307] Scordalakes E. M. Imwalle D. B. Rissman E. F. (2002). Oestrogen's masculine side: mediation of mating in male mice. Reproduction 124, 331–338. 10.1530/rep.0.1240331 12201806

[B308] Sellers K. J. Erli F. Raval P. Watson I. A. Chen D. Srivastava D. P. (2015). Rapid modulation of synaptogenesis and spinogenesis by 17beta-estradiol in primary cortical neurons. Front. Cell. Neurosci. 9:137. 10.3389/fncel.2015.00137 25926772 PMC4396386

[B309] Seredynski A. L. Balthazart J. Ball G. F. Cornil C. A. (2015). Estrogen receptor beta activation rapidly modulates male sexual motivation through the transactivation of metabotropic glutamate receptor 1a. J. Neurosci. 35, 13110–13123. 10.1523/JNEUROSCI.2056-15.2015 26400941 PMC4579376

[B310] Seredynski A. L. Balthazart J. Christophe V. J. Ball G. F. Cornil C. A. (2013). Neuroestrogens rapidly regulate sexual motivation but not performance. J. Neurosci. 33, 164–174. 10.1523/JNEUROSCI.2557-12.2013 23283331 PMC3710137

[B311] Shah A. H. Chin E. H. Schmidt K. L. Soma K. K. (2011). DHEA and estradiol levels in brain, gonads, adrenal glands, and plasma of developing male and female European starlings. J. Comp. Physiol. A Neuroethol. Sens. Neural Behav. Physiol. 197, 949–958. 10.1007/s00359-011-0655-4 21691747

[B312] Shahrokhi N. Haddad M. K. Joukar S. Shabani M. Keshavarzi Z. Shahozehi B. (2012). Neuroprotective antioxidant effect of sex steroid hormones in traumatic brain injury. Pak. J. Pharm. Sci. 25, 219–225. 22186333

[B313] Shi Y. Liu X. Zhu P. Li J. Sham K. W. Cheng S. H.. (2013). G-protein-coupled estrogen receptor 1 is involved in brain development during zebrafish (*Danio rerio*) embryogenesis. Biochem. Biophys. Res. Commun. 435, 21–27. 10.1016/j.bbrc.2013.03.130 23583372

[B314] Shughrue P. J. Lane M. V. Merchenthaler I. (1997). Comparative distribution of estrogen receptor-alpha and -beta mRNA in the rat central nervous system. J. Comp. Neurol. 388, 507–525. 10.1002/(SICI)1096-9861(19971201)388:4<507::AID-CNE1>3.0.CO;2-6 9388012

[B315] Si D. Li J. Liu J. Wang X. Wei Z. Tian Q.. (2014). Progesterone protects blood-brain barrier function and improves neurological outcome following traumatic brain injury in rats. Exp. Ther. Med. 8, 1010–1014. 10.3892/etm.2014.1840 25120639 PMC4113529

[B316] Sinchak K. Mills R. H. Tao L. LaPolt P. Lu J. K. Micevych P. (2003). Estrogen induces *de novo* progesterone synthesis in astrocytes. Dev. Neurosci. 25, 343–348. 10.1159/000073511 14614261

[B317] Slewa-Younan S. Green A. M. Baguley I. J. Gurka J. A. Marosszeky J. E. (2004). Sex differences in injury severity and outcome measures after traumatic brain injury. Arch. Phys. Med. Rehabil. 85, 376–379. 10.1016/j.apmr.2003.05.007 15031820

[B318] Socorro S. Power D. M. Olsson P. E. Canario A. V. (2000). Two estrogen receptors expressed in the teleost fish, *Sparus aurata*: cDNA cloning, characterization and tissue distribution. J. Endocrinol. 166, 293–306. 10.1677/joe.0.1660293 10927619

[B319] Södersten P. (2013). A historical and personal perspective on the aromatization revolution, in Brain Aromatase, Estrogens, and Behavior, eds Balthazart J. Ball G. F. (New York, NY; Oxford UP), 281–314.

[B320] Sohrabji F. (2015). Cerebrovascular stroke: sex differences and the impact of Estrogens, in Estrogen Effects on Traumatic Brain Injury - Mechanisms of Neuroprotection and Repair, ed Duncan Kelli A. (Amsterdam: Academic Press Elsevier), 125–141.

[B321] Soma K. K. Alday N. A. Hau M. Schlinger B. A. (2004). Dehydroepiandrosterone metabolism by 3beta-hydroxysteroid dehydrogenase/Delta5-Delta4 isomerase in adult zebra finch brain: sex difference and rapid effect of stress. Endocrinology 145, 1668–1677. 10.1210/en.2003-0883 14670998

[B322] Soma K. K. Sinchak K. Lakhter A. Schlinger B. A. Micevych P. E. (2005). Neurosteroids and female reproduction: estrogen increases 3beta-HSD mRNA and activity in rat hypothalamus. Endocrinology 146, 4386–4390. 10.1210/en.2005-0569 16020475 PMC2877701

[B323] Sperry T. S. Thomas P. (1999a). Characterization of two nuclear androgen receptors in Atlantic croaker: comparison of their biochemical properties and binding specificities. Endocrinology 140, 1602–1611. 10.1210/endo.140.4.6631 10098494

[B324] Sperry T. S. Thomas P. (1999b). Identification of two nuclear androgen receptors in kelp bass (*Paralabrax clathratus*) and their binding affinities for xenobiotics: comparison with Atlantic croaker (*Micropogonias undulatus*) androgen receptors. Biol. Reprod. 61, 1152–1161. 10.1095/biolreprod61.4.1152 10491657

[B325] Spritzer M. D. Galea L. A. (2007). Testosterone and dihydrotestosterone, but not estradiol, enhance survival of new hippocampal neurons in adult male rats. Dev. Neurobiol. 67, 1321–1333. 10.1002/dneu.20457 17638384

[B326] Stephens S. B. Tolson K. P. Rouse M. L. Jr. Poling M. C. Hashimoto-Partyka M. K. Mellon P. L.. (2015). Absent progesterone signaling in kisspeptin neurons disrupts the LH surge and impairs fertility in female mice. Endocrinology 156, 3091–3097. 10.1210/en.2015-1300 26076042 PMC4541622

[B327] Sterling R. J. Gasc J. M. Sharp P. J. Renoir J. M. Tuohimaa P. Baulieu E. E. (1987). The distribution of nuclear progesterone receptor in the hypothalamus and forebrain of the domestic hen. Cell Tissue Res. 248, 201–205. 10.1007/BF01239981 3552237

[B328] Stewart P. A. Wiley M. J. (1981). Structural and histochemical features of the avian blood-brain barrier. J. Comp. Neurol. 202, 157–167. 10.1002/cne.902020203 7298896

[B329] Stone D. J. Rozovsky I. Morgan T. E. Anderson C. P. Hajian H. Finch C. E. (1997). Astrocytes and microglia respond to estrogen with increased apoE mRNA *in vivo* and *in vitro*. Exp. Neurol. 143, 313–318. 10.1006/exnr.1996.6360 9056393

[B330] Strobl-Mazzulla P. H. Lethimonier C. Gueguen M. M. Karube M. Fernandino J. I. Yoshizaki G.. (2008). Brain aromatase (Cyp19A2) and estrogen receptors, in larvae and adult pejerrey fish *Odontesthes bonariensis*: neuroanatomical and functional relations. Gen. Comp. Endocrinol. 158, 191–201. 10.1016/j.ygcen.2008.07.006 18691594

[B331] Strobl-Mazzulla P. H. Moncaut N. P. López G. C. Miranda L. A. Canario A. V. Somoza G. M. (2005). Brain aromatase from pejerrey fish (*Odontesthes bonariensis*): cDNA cloning, tissue expression, and immunohistochemical localization. Gen. Comp. Endocrinol. 143, 21–32. 10.1016/j.ygcen.2005.02.026 15993101

[B332] Strobl-Mazzulla P. H. Nuñez A. Pellegrini E. Gueguen M. M. Kah O. Somoza G. M. (2010). Progenitor radial cells and neurogenesis in pejerrey fish forebrain. Brain Behav. Evol. 76, 20–31. 10.1159/000316022 20798479

[B333] Strömstedt M. Waterman M. R. (1995). Messenger RNAs encoding steroidogenic enzymes are expressed in rodent brain. Brain Res. Mol. Brain Res. 34, 75–88. 10.1016/0169-328X(95)00140-N 8750863

[B334] Sugiyama D. Kusuhara H. Shitara Y. Abe T. Meier P. J. Sekine T.. (2001). Characterization of the efflux transport of 17beta-estradiol-D-17beta-glucuronide from the brain across the blood-brain barrier. J. Pharmacol. Exp. Ther. 298, 316–322. 11408557

[B335] Sugiyama N. Andersson S. Lathe R. Fan X. Alonso-Magdalena P. Schwend T.. (2009). Spatiotemporal dynamics of the expression of estrogen receptors in the postnatal mouse brain. Mol. Psychiatry 14, 223–232. 10.1038/mp.2008.118 18982005

[B336] Suzuki S. Gerhold L. M. Böttner M. Rau S. W. Dela Cruz C. Yang E.. (2007). Estradiol enhances neurogenesis following ischemic stroke through estrogen receptors alpha and beta. J. Comp. Neurol. 500, 1064–1075. 10.1002/cne.21240 17183542

[B337] Takeo J. Yamashita S. (1999). Two distinct isoforms of cDNA encoding rainbow trout androgen receptors. J. Biol. Chem. 274, 5674–5680. 10026186 10.1074/jbc.274.9.5674

[B338] Takeo J. Yamashita S. (2000). Rainbow trout androgen receptor-alpha fails to distinguish between any of the natural androgens tested in transactivation assay, not just 11-ketotestosterone and testosterone. Gen. Comp. Endocrinol. 117, 200–206. 10.1006/gcen.1999.7398 10642442

[B339] Tan N. S. Lam T. J. Ding J. L. (1995). The hormone-binding domain of *Oreochromis aureus* estrogen receptor gene: homology comparison with other steroid binding receptors. DNA Seq. 5, 371–379. 8777316 10.3109/10425179509020868

[B340] Tanapat P. Hastings N. B. Gould E. (2005). Ovarian steroids influence cell proliferation in the dentate gyrus of the adult female rat in a dose- and time-dependent manner. J. Comp. Neurol. 481, 252–265. 10.1002/cne.20385 15593136

[B341] Tanapat P. Hastings N. B. Reeves A. J. Gould E. (1999). Estrogen stimulates a transient increase in the number of new neurons in the dentate gyrus of the adult female rat. J. Neurosci. 19, 5792–5801. 10407020 10.1523/JNEUROSCI.19-14-05792.1999 PMC6783062

[B342] Tang H. Liu Y. Li J. Yin Y. Li G. Chen Y.. (2016). Gene knockout of nuclear progesterone receptor provides insights into the regulation of ovulation by LH signaling in zebrafish. Sci. Rep. 6:28545. 10.1038/srep28545 27333837 PMC4917859

[B343] Taziaux M. Keller M. Bakker J. Balthazart J. (2007). Sexual behavior activity tracks rapid changes in brain estrogen concentrations. J. Neurosci. 27, 6563–6572. 10.1523/JNEUROSCI.1797-07.2007 17567817 PMC6672433

[B344] Tchoudakova A. Pathak S. Callard G. V. (1999). Molecular cloning of an estrogen receptor beta subtype from the goldfish, *Carassius auratus*. Gen. Comp. Endocrinol. 113, 388–400. 10.1006/gcen.1998.7217 10068500

[B345] Thomas P. (2008). Characteristics of membrane progestin receptor alpha (mPRalpha) and progesterone membrane receptor component 1 (PGMRC1) and their roles in mediating rapid progestin actions. Front. Neuroendocrinol. 29, 292–312. 10.1016/j.yfrne.2008.01.001 18343488 PMC2600886

[B346] Thompson R. R. George K. Dempsey J. Walton J. C. (2004). Visual sex discrimination in goldfish: seasonal, sexual, and androgenic influences. Horm. Behav. 46, 646–654. 10.1016/j.yhbeh.2004.06.008 15555507

[B347] Thornton J. W. (2001). Evolution of vertebrate steroid receptors from an ancestral estrogen receptor by ligand exploitation and serial genome expansions. Proc. Natl. Acad. Sci. U.S.A. 98, 5671–5676. 10.1073/pnas.091553298 11331759 PMC33271

[B348] Tikkanen M. J. Vihma V. Jauhiainen M. Höckerstedt A. Helisten H. Kaamanen M. (2002). Lipoprotein-associated estrogens. Cardiovasc. Res. 56, 184–188. 10.1016/S0008-6363(02)00535-7 12393088

[B349] Timmers R. J. Lambert J. G. (1987). Measurement of aromatase activity in the brain of the African catfish, *Clarias gariepinus*–a comparison of two assay methods. Comp. Biochem. Physiol. B Comp. Biochem. 88, 453–456. 10.1016/0305-0491(87)90325-7 3427894

[B350] Tokumoto M. Nagahama Y. Thomas P. Tokumoto T. (2006). Cloning and identification of a membrane progestin receptor in goldfish ovaries and evidence it is an intermediary in oocyte meiotic maturation. Gen. Comp. Endocrinol. 145, 101–108. 10.1016/j.ygcen.2005.07.002 16139281

[B351] Tokumoto T. Tokumoto M. Oshima T. Shimizuguchi K. Fukuda T. Sugita E.. (2012). Characterization of multiple membrane progestin receptor (mPR) subtypes from the goldfish ovary and their roles in the induction of oocyte maturation. Gen. Comp. Endocrinol. 177, 168–176. 10.1016/j.ygcen.2012.03.005 22465781

[B352] Tomy S. Wu G. C. Huang H. R. Dufour S. Chang C. F. (2007). Developmental expression of key steroidogenic enzymes in the brain of protandrous black porgy fish, *Acanthopagrus schlegeli*. J. Neuroendocrinol. 19, 643–655. 10.1111/j.1365-2826.2007.01572.x 17620106

[B353] Tong S. K. Mouriec K. Kuo M. W. Pellegrini E. Gueguen M. M. Brion F.. (2009). A cyp19a1b-gfp (aromatase B) transgenic zebrafish line that expresses GFP in radial glial cells. Genesis 47, 67–73. 10.1002/dvg.20459 19101983

[B354] Toran-Allerand C. D. (2005). Estrogen and the brain: beyond ER-alpha, ER-beta, and 17beta-estradiol. Ann. N. Y. Acad. Sci. 1052, 136–144. 10.1196/annals.1347.009 16024756

[B355] Toran-Allerand C. D. Guan X. MacLusky N. J. Horvath T. L. Diano S. Singh M.. (2002). ER-X: a novel, plasma membrane-associated, putative estrogen receptor that is regulated during development and after ischemic brain injury. J. Neurosci. 22, 8391–8401. 12351713 10.1523/JNEUROSCI.22-19-08391.2002 PMC6757764

[B356] Tramontin A. D. Wingfield J. C. Brenowitz E. A. (2003). Androgens and estrogens induce seasonal-like growth of song nuclei in the adult songbird brain. J. Neurobiol. 57, 130–140. 10.1002/neu.10263 14556279

[B357] Tran-Dinh A. Diallo D. Delbosc S. Varela-Perez L. M. Dang Q. B. Lapergue B.. (2013). HDL and endothelial protection. Br. J. Pharmacol. 169, 493–511. 10.1111/bph.12174 23488589 PMC3682699

[B358] Tsutsui K. (2008). Progesterone biosynthesis and action in the developing neuron. Endocrinology 149, 2757–2761. 10.1210/en.2007-1592 18308850

[B359] Tsutsui K. (2011). Neurosteroid biosynthesis and function in the brain of domestic birds. Front. Endocrinol. 2:37. 10.3389/fendo.2011.00037 22645509 PMC3355851

[B360] Tsutsui K. Ukena K. (1999). Neurosteroids in the cerebellar Purkinje neuron and their actions (review). Int. J. Mol. Med. 4, 49–56. 10.3892/ijmm.4.1.49 10373637

[B361] Tsutsui K. Yamazaki T. (1995). Avian neurosteroids. I. Pregnenolone biosynthesis in the quail brain. Brain Res. 678, 1–9. 10.1016/0006-8993(95)00116-8 7620878

[B362] Tsutsui K. Haraguchi S. Inoue K. Miyabara H. Suzuki S. Ogura Y.. (2009). Identification, biosynthesis, and function of 7alpha-hydroxypregnenolone, a new key neurosteroid controlling locomotor activity, in nonmammalian vertebrates. Ann. N. Y. Acad. Sci. 1163, 308–315. 10.1111/j.1749-6632.2009.04426.x 19456352

[B363] Tsutsui K. Matsunaga M. Miyabara H. Ukena K. (2006). Neurosteroid biosynthesis in the quail brain: a review. J. Exp. Zool. A Comp. Exp. Biol. 305, 733–742. 10.1002/jez.a.302 16902960

[B364] Tsutsui K. Ukena K. Takase M. Kohchi C. Lea R. W. (1999). Neurosteroid biosynthesis in vertebrate brains. Comp. Biochem. Physiol. C Pharmacol. Toxicol. Endocrinol. 124, 121–129. 10.1016/S0742-8413(99)00065-1 10622427

[B365] Ukena K. Honda Y. Inai Y. Kohchi C. Lea R. W. Tsutsui K. (1999). Expression and activity of 3beta-hydroxysteroid dehydrogenase/Delta5-Delta4-isomerase in different regions of the avian brain. Brain Res. 818, 536–542. 10.1016/S0006-8993(98)01296-7 10082843

[B366] Vance J. E. Hayashi H. (2010). Formation and function of apolipoprotein E-containing lipoproteins in the nervous system. Biochim. Biophys. Acta 1801, 806–818. 10.1016/j.bbalip.2010.02.007 20170744

[B367] Vitali C. Wellington C. L. Calabresi L. (2014). HDL and cholesterol handling in the brain. Cardiovasc. Res. 103, 405–413. 10.1093/cvr/cvu148 24907980

[B368] Wakai S. Hirokawa N. (1978). Development of the blood-brain barrier to horseradish peroxidase in the chick embryo. Cell Tissue Res. 195, 195–203. 10.1007/BF00236719 737715

[B369] Wang C. Liu D. Chen W. Ge W. Hong W. Zhu Y.. (2016). Progestin increases the expression of gonadotropins in pituitaries of male zebrafish. J. Endocrinol. 230, 143–156. 10.1530/JOE-16-0073 27113852 PMC4938713

[B370] Wang L. Andersson S. Warner M. Gustafsson J. A. (2003). Estrogen receptor (ER)beta knockout mice reveal a role for ERbeta in migration of cortical neurons in the developing brain. Proc. Natl. Acad. Sci. U.S.A. 100, 703–708. 10.1073/pnas.242735799 12515851 PMC141060

[B371] Waters E. M. Thompson L. I. Patel P. Gonzales A. D. Ye H. Z. Filardo E. J.. (2015). G-protein-coupled estrogen receptor 1 is anatomically positioned to modulate synaptic plasticity in the mouse hippocampus. J. Neurosci. 35, 2384–2397. 10.1523/JNEUROSCI.1298-14.2015 25673833 PMC4323523

[B372] Watson J. T. Adkins-Regan E. (1989a). Testosterone implanted in the preoptic area of male Japanese quail must be aromatized to activate copulation. Horm. Behav. 23, 432–447. 10.1016/0018-506X(89)90055-X 2793083

[B373] Watson J. T. Adkins-Regan E. (1989b). Activation of sexual behavior by implantation of testosterone propionate and estradiol benzoate into the preoptic area of the male Japanese quail (*Coturnix japonica*). Horm. Behav. 23, 251–268. 10.1016/0018-506X(89)90065-2 2744741

[B374] Weger M. Diotel N. Weger B. D. Beil T. Zaucker A. Eachus H. L. (accepted). Expression profiling of the steroidogenic enzymes of glucocorticoid biosynthesis the fdx1 co-factors in zebrafish. J. Neuroendocrinol. 10.1111/jne.12586 29486070

[B375] Weidenfeld J. Siegel R. A. Chowers I. (1980). *In vitro* conversion of pregnenolone to progesterone by discrete brain areas of the male rat. J. Steroid Biochem. 13, 961–963. 10.1016/0022-4731(80)90171-5 7464142

[B376] Weissman T. Noctor S. C. Clinton B. K. Honig L. S. Kriegstein A. R. (2003). Neurogenic radial glial cells in reptile, rodent and human: from mitosis to migration. Cereb. Cortex 13, 550–559. 10.1093/cercor/13.6.550 12764028

[B377] Witt K. A. Sandoval K. E. (2014). Steroids and the blood-brain barrier: therapeutic implications. Adv. Pharmacol. 71, 361–390. 10.1016/bs.apha.2014.06.018 25307223

[B378] Xia Z. Patiño R. Gale W. L. Maule A. G. Densmore L. D. (1999). Cloning, *in vitro* expression, and novel phylogenetic classification of a channel catfish estrogen receptor. Gen. Comp. Endocrinol. 113, 360–368. 10.1006/gcen.1999.7196 10068497

[B379] Xiao H. Deng M. Yang B. Hu Z. Tang J. (2017). Pre-treatment of 17beta-estradiol Attenuates cerebral-ischemia-induced blood-brain barrier disruption in aged rats: involvement of antioxidant signaling. Neuroendocrinology. 10.1159/000455866 28196366

[B380] Xing L. Esau C. Trudeau V. L. (2015). Direct regulation of aromatase B expression by 17beta-estradiol and dopamine D1 receptor agonist in adult radial glial cells. Front. Neurosci. 9:504. 10.3389/fnins.2015.00310 26793050 PMC4709857

[B381] Xing L. Goswami M. Trudeau V. L. (2014). Radial glial cell: critical functions and new perspective as a steroid synthetic cell. Gen. Comp. Endocrinol. 203, 181–185. 10.1016/j.ygcen.2014.03.010 24675515

[B382] Xing L. Martyniuk C. J. Esau C. Da Fonte D. F. Trudeau V. L. (2016). Proteomic profiling reveals dopaminergic regulation of progenitor cell functions of goldfish radial glial cells *in vitro*. J. Proteomics 144, 123–132. 10.1016/j.jprot.2016.05.003 27185549

[B383] Yague J. G. Muñoz A. de Monasterio-Schrader P. Defelipe J. Garcia-Segura L. M. Azcoitia I. (2006). Aromatase expression in the human temporal cortex. Neuroscience 138, 389–401. 10.1016/j.neuroscience.2005.11.054 16426763

[B384] Yu L. Romero D. G. Gomez-Sanchez C. E. Gomez-Sanchez E. P. (2002). Steroidogenic enzyme gene expression in the human brain. Mol. Cell. Endocrinol. 190, 9–17. 10.1016/S0303-7207(02)00041-2 11997174

[B385] Zempo B. Kanda S. Okubo K. Akazome Y. Oka Y. (2013). Anatomical distribution of sex steroid hormone receptors in the brain of female medaka. J. Comp. Neurol. 521, 1760–1780. 10.1002/cne.23255 23124931

[B386] Zhang J. Liu Q. (2015). Cholesterol metabolism and homeostasis in the brain. Protein Cell 6, 254–264. 10.1007/s13238-014-0131-3 25682154 PMC4383754

[B387] Zhang P. Xie M. Q. Ding Y. Q. Liao M. Qi S. S. Chen S. X.. (2015). Allopregnanolone enhances the neurogenesis of midbrain dopaminergic neurons in APPswe/PSEN1 mice. Neuroscience 290, 214–226. 10.1016/j.neuroscience.2015.01.019 25637494

[B388] Zhang W. Cheng J. Vagnerova K. Ivashkova Y. Young J. Cornea A.. (2014). Effects of androgens on early post-ischemic neurogenesis in mice. Transl. Stroke Res. 5, 301–311. 10.1007/s12975-013-0298-6 24323721

[B389] Zhang Y. Wang H. Qin F. Liu S. Wu T. Li M.. (2012). Molecular characterization of estrogen receptor genes in loach *Paramisgurnus dabryanus* and their expression upon 17alpha-ethinylestradiol exposure in juveniles. Gen. Comp. Endocrinol. 178, 194–205. 10.1016/j.ygcen.2012.06.004 22705038

[B390] Zhang Z. Yang R. Zhou R. Li L. Sokabe M. Chen L. (2010). Progesterone promotes the survival of newborn neurons in the dentate gyrus of adult male mice. Hippocampus 20, 402–412. 10.1002/hipo.20642 19475650

[B391] Zhao Y. Wang J. Liu C. Jiang C. Zhao C. Zhu Z. (2011). Progesterone influences postischemic synaptogenesis in the CA1 region of the hippocampus in rats. Synapse 65, 880–891. 10.1002/syn.20915 21308798

[B392] Zhu Y. Bond J. Thomas P. (2003). Identification, classification, and partial characterization of genes in humans and other vertebrates homologous to a fish membrane progestin receptor. Proc. Natl. Acad. Sci. U.S.A. 100, 2237–2242. 10.1073/pnas.0436133100 12601167 PMC151324

[B393] Zhu Y. Liu D. Shaner Z. C. Chen S. Hong W. Stellwag E. J. (2015). Nuclear progestin receptor (pgr) knockouts in zebrafish demonstrate role for pgr in ovulation but not in rapid non-genomic steroid mediated meiosis resumption. Front. Endocrinol. 6:37. 10.3389/fendo.2015.00037 25852646 PMC4365747

[B394] Zuloaga D. G. Yahn S. L. Pang Y. Quihuis A. M. Oyola M. G. Reyna A.. (2012). Distribution and estrogen regulation of membrane progesterone receptor-beta in the female rat brain. Endocrinology 153, 4432–4443. 10.1210/en.2012-1469 22778216 PMC3423618

[B395] Zwain I. H. Yen S. S. (1999). Neurosteroidogenesis in astrocytes, oligodendrocytes, and neurons of cerebral cortex of rat brain. Endocrinology 140, 3843–3852. 10.1210/endo.140.8.6907 10433246

